# Supplementary material for: Australia as a global sink for the genetic diversity of avian influenza A virus
Source: PLoS Pathog. 2022 May 10;18(5):e1010150. doi: 10.1371/journal.ppat.1010150 (PMC9089890; doi:10.1371/journal.ppat.1010150)

To evaluate whether a Ct threshold of 30 was an appropriate cut-off for sequencing of original samples we compared Ct values with genome completeness outcomes. We found that genome completeness is influenced by Ct values (X^2=35.49, df=3, p < 0.001), whereby we found a statistical difference in the Ct values of genomes missing segments and those that were complete (p<0.001). We found no statistical difference in Ct values between genomes that were complete and those with all segments present but in some cases we were unable to recover the entire segment length (Figure S1). Further, using generalised linear models, we found that the likelihood of successfully sequencing a virus with a complete genome increased rapidly with decreasing Ct value, with 50% chance of success for Ct = 28.6, 75% chance of success with Ct = 25.3 and 90% chance of success with Ct = 22 (Figure S1). At a Ct of, we found a 39% probability of rendering a complete genome, 44% probability of rendering a genome with all segments but some partial and a 91% probability of completing at least 1 segment suggesting a cut of Ct 28 may be more appropriate to increase the likelihood of garnering a complete genome.

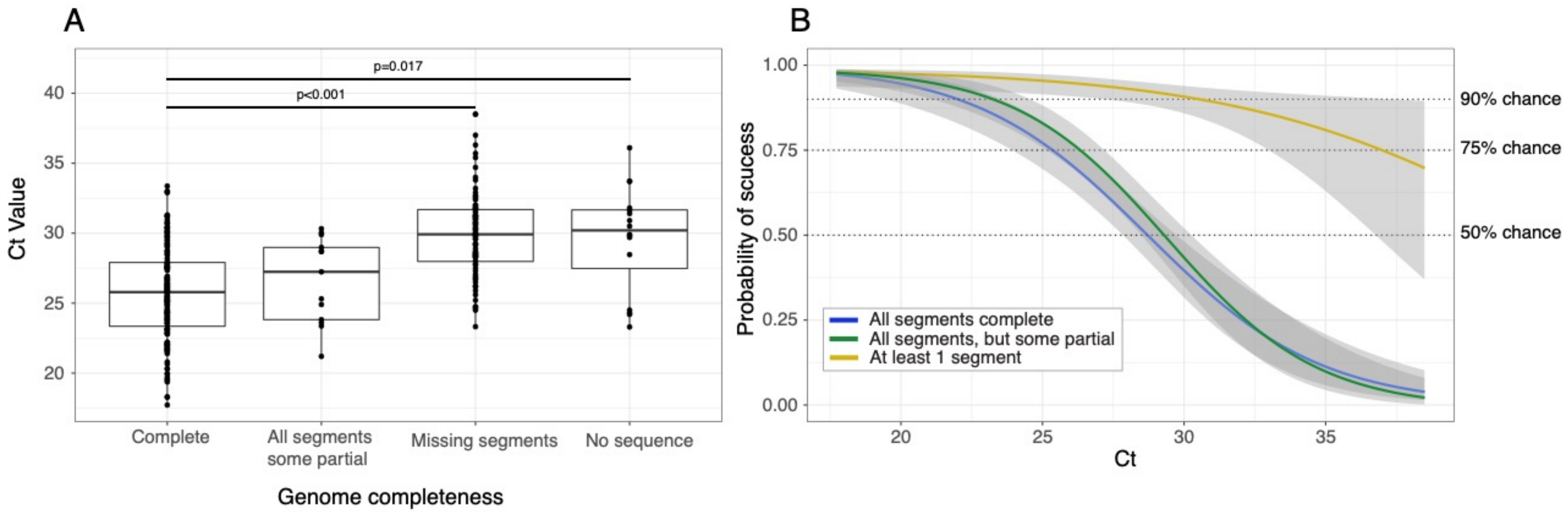

Supplement: S1 Fig — (A) Data are presented as box plots presenting the median Ct value for each category, in addition to the interquartile range. Data points have been plotted as points on top of the boxplots. P-values for statistically significant comparisons are presented. (B) Effect of Ct value on probability of genome completeness. Y value is the probability, from 0 to 1. Lines correspond to generalised linear model and shading is the 95% confidence interval of the model. Dotted lines represent the 50%, 75% and 90% thresholds (PDF) [file ppat.1010150.s001.pdf]
