# Supplementary figures and images for: Australia as a global sink for the genetic diversity of avian influenza A virus
Source: PLoS Pathog. 2022 May 10;18(5):e1010150. doi: 10.1371/journal.ppat.1010150 (PMC9089890; doi:10.1371/journal.ppat.1010150)

A

Number of Genome

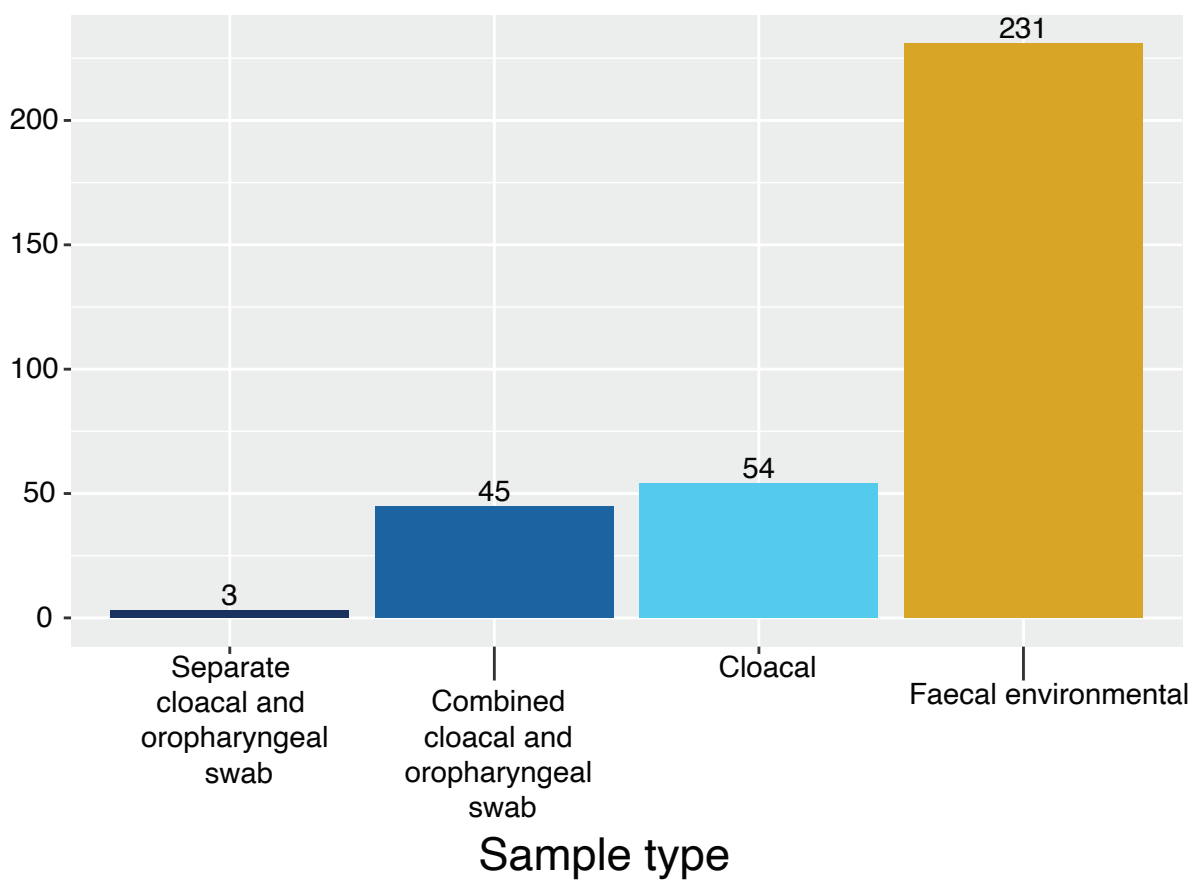

B

Number of Genomes

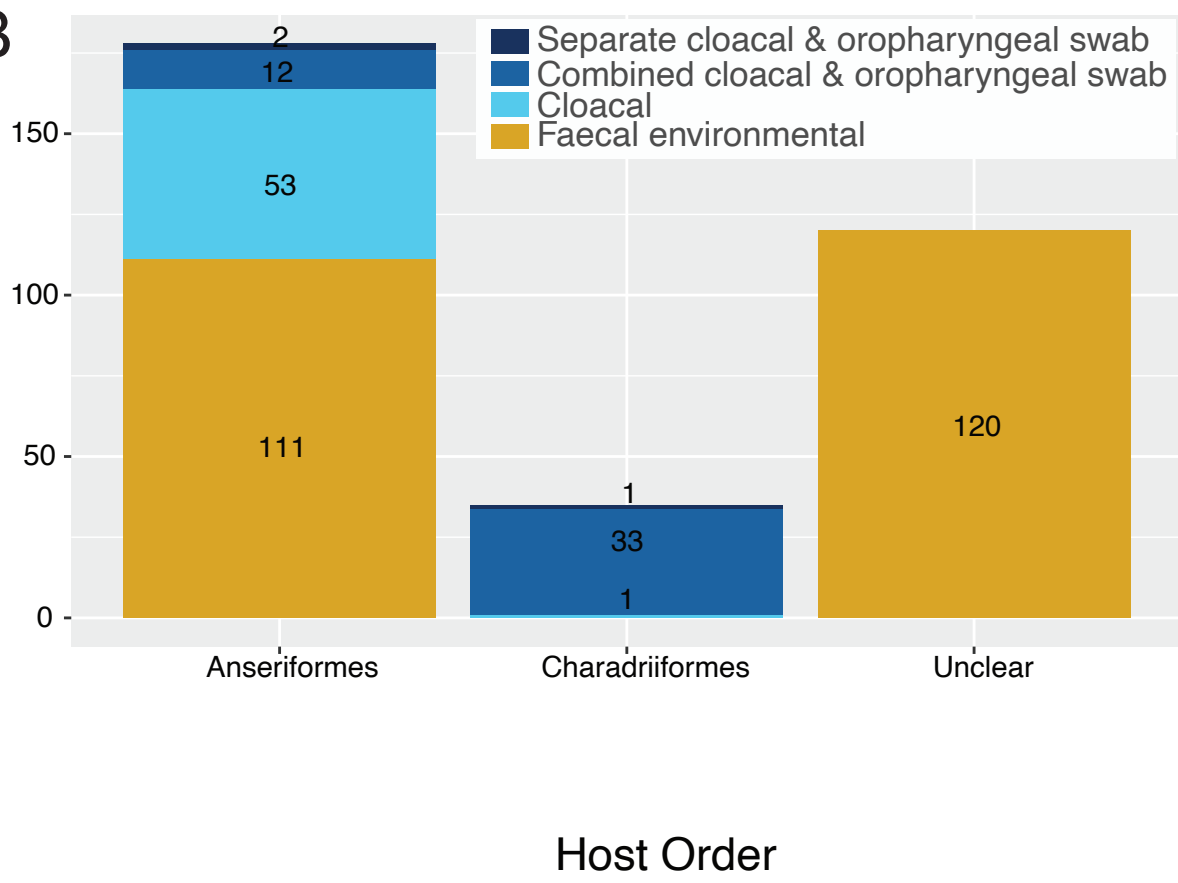

C

Number of Genomes

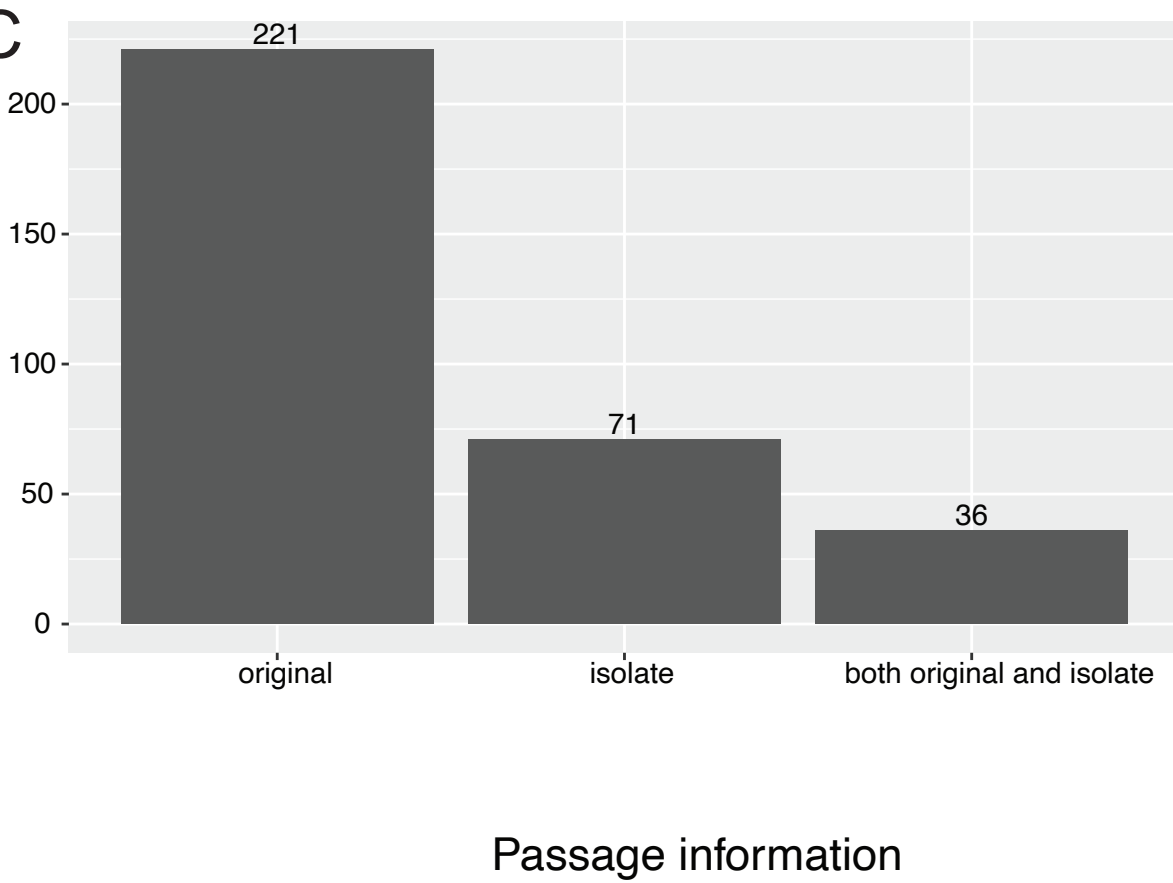

Supplement: S2 Fig — (A) Distribution of genomes by sample type. (B) Distribution of genomes by host type, and coloured by sample type. The colours in panel A and B are the same. In cases where Host Order is “unclear”, samples were collected from mixed flocks of birds or insufficient metadata were provided. (C). Passage information of samples, including “original” wherein the original sample was submitted for sequencing. “isolate” includes any number of passages following egg isolation. A number of samples were sequenced both from original sample and from the isolate. In general, this was done as sequencing the original sample resulted in an incomplete genome, and sequencing the isolate increased the likelihood of getting a full genome. Data here do not include sequences from Hoye et al. 2021 [40] or Bhutta et al. 2020 [41]. The dataset used to build panel A and B comprise no duplicates, and genomes must have at least 1 segment. The dataset used to build panel C includes duplicates, and genomes must have at least 1 segment. (PDF) [file ppat.1010150.s002.pdf]

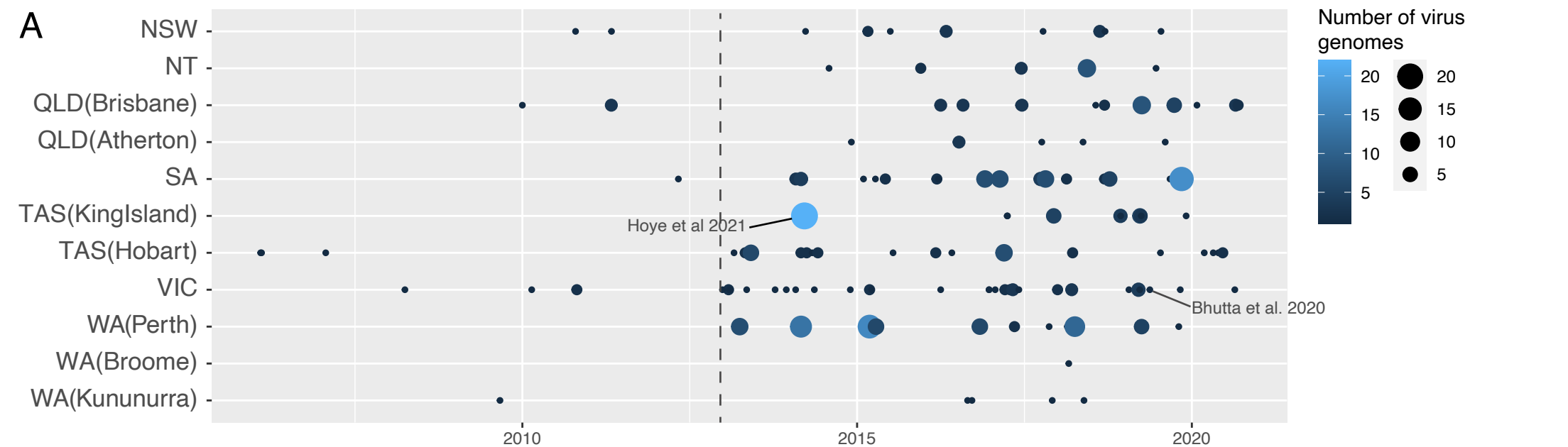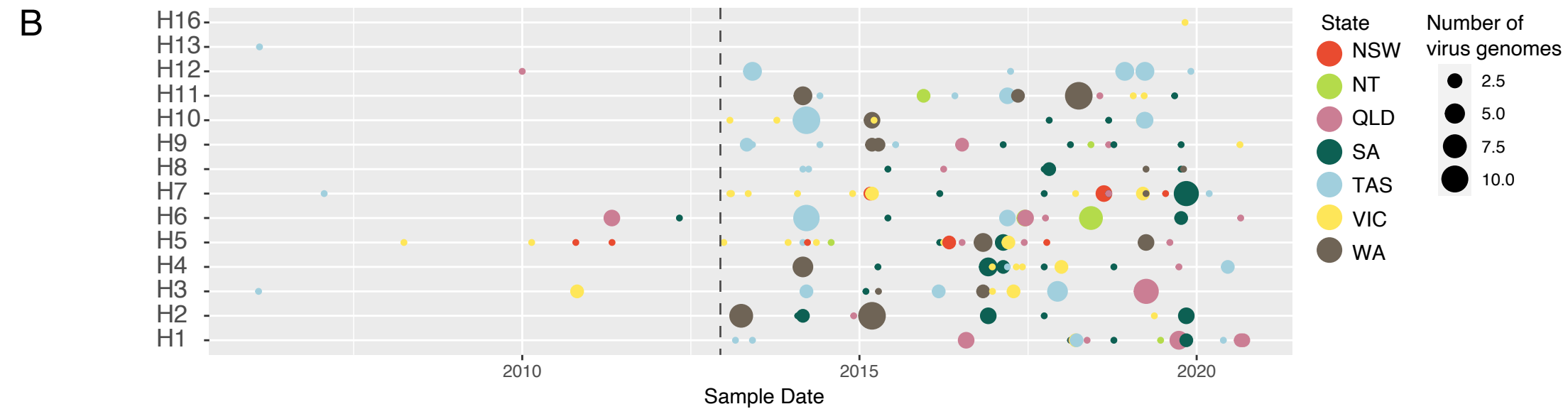

Supplement: S3 Fig — (A) States are plotted on the Y axis, and for states with sampling sites >1000km apart, the sampling areas are indicated. These locations match those from Fig 1A. Data from Hoye et al. 2021 [40] and Bhutta et al. 2020 [41] are indicated. Both circle size and colour refer to number of genomes. (B) HA subtypes are plotted on the Y-axis, and circle colour refer to the Australian state. Each circle corresponds to a sampling event, plotted by date, with circle size refer to the number of genomes from that event. Dashed line indicates when the aim of the NAIWB shifted from primarily H5/H7 detection and characterization to characterisation of all subtypes. These data do not include duplicate genome sequences of individual viruses, and virus genomes must have at least 1 segment sequenced (PDF) [file ppat.1010150.s003.pdf]

A

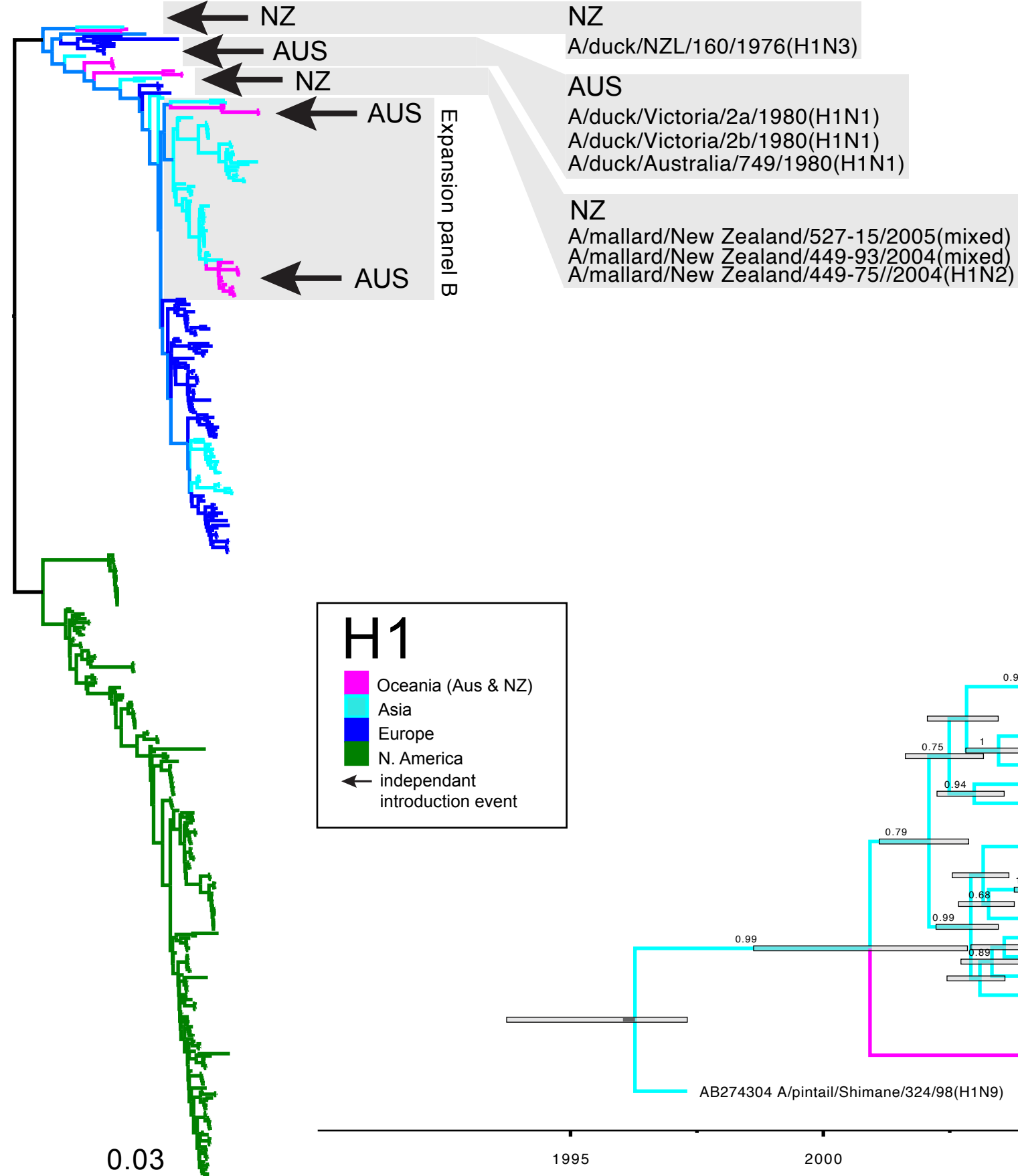

B

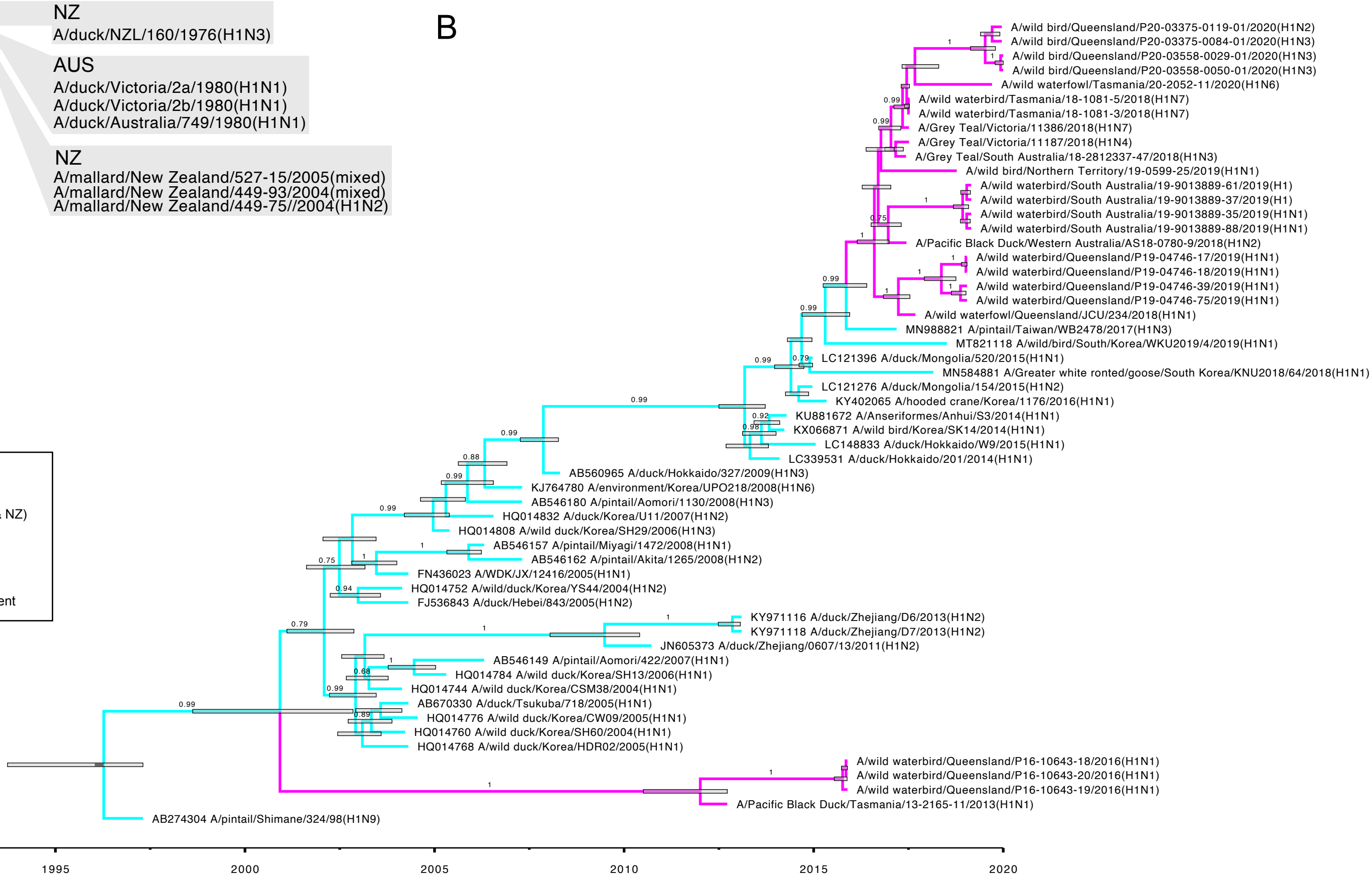

Supplement: S4 Fig — (A) Maximum likelihood tree of the sequences generated in this study, all sequences from Oceania in GenBank and reference sequences from Europe, Asia and North America. Lineages from Oceania are highlighted in grey boxes and virus names are provided. (B) Time structured phylogenetic tree comprising contemporary clades present in Australia. Node bars correspond to the 95% highest posterior density (HDP) of node height. Branches are coloured based on geography as indicated on the legend. (PDF) [file ppat.1010150.s004.pdf]

A

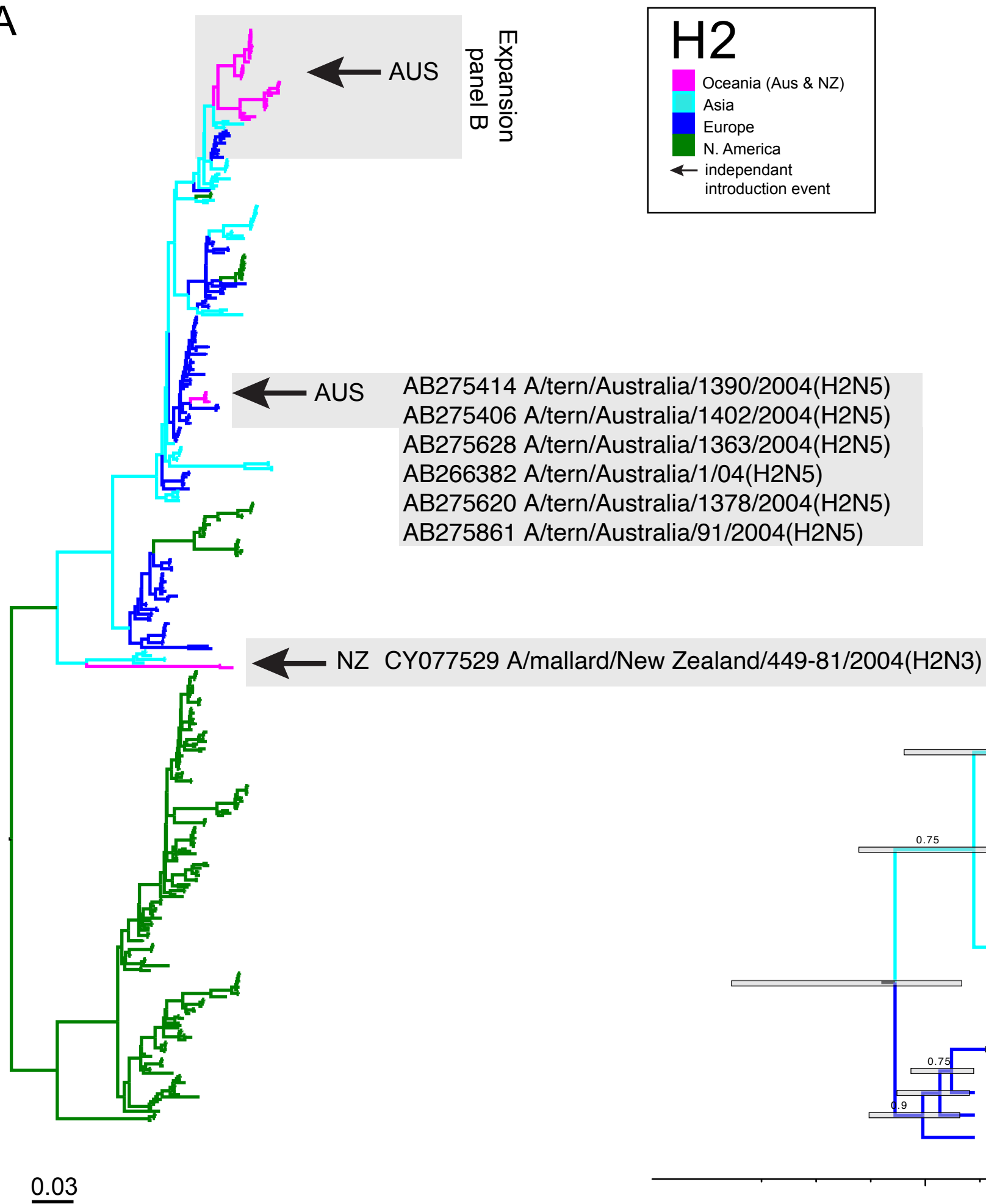

B

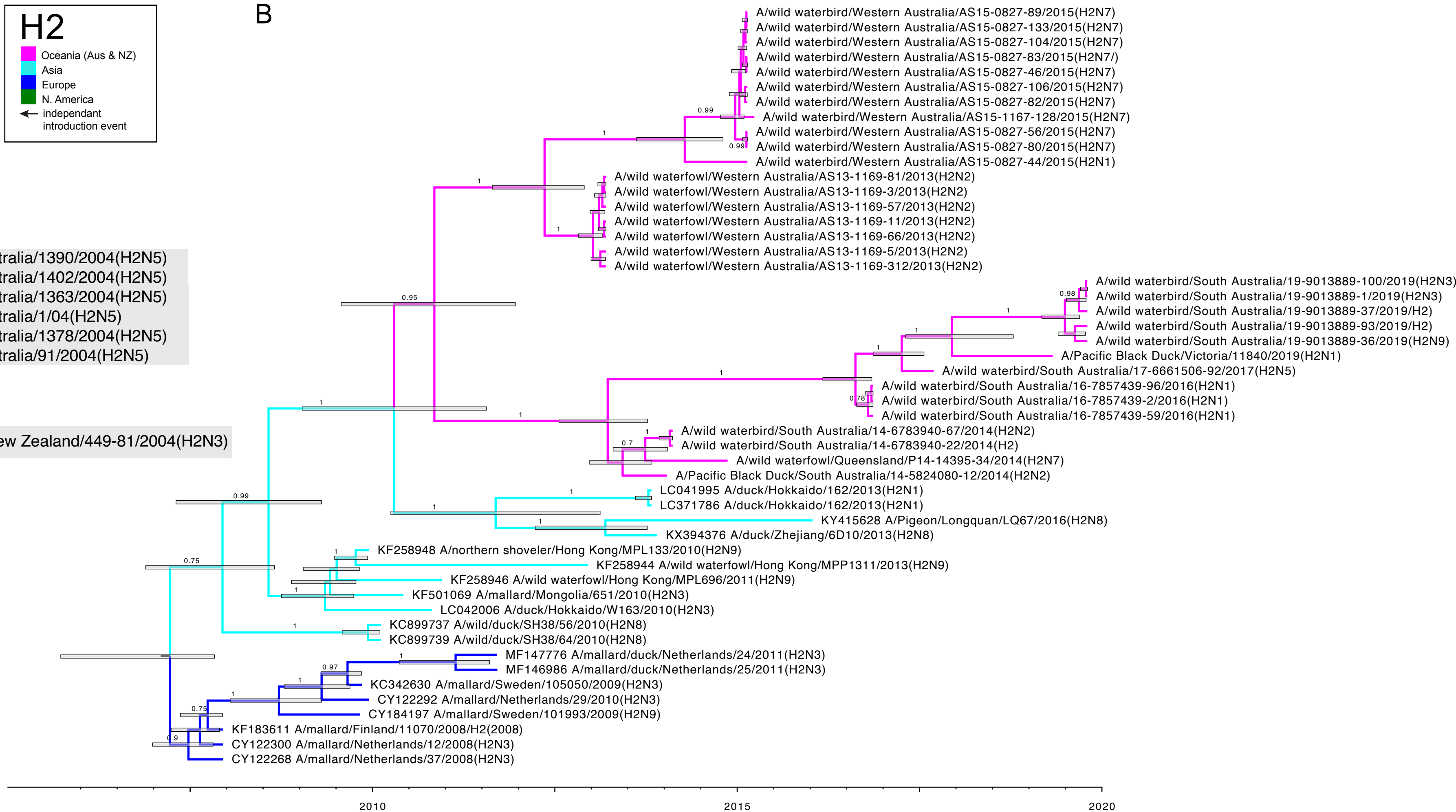

Supplement: S5 Fig — (A) Maximum likelihood tree of the sequences generated in this study, all sequences from Oceania in GenBank and reference sequences from Europe, Asia and North America. Lineages from Oceania are highlighted in grey boxes and virus names are provided. (B) Time structured phylogenetic tree comprising contemporary clades present in Australia. Node bars correspond to the 95% highest posterior density (HDP) of node height. Branches are coloured based on geography as indicated on the legend. (PDF) [file ppat.1010150.s005.pdf]

A

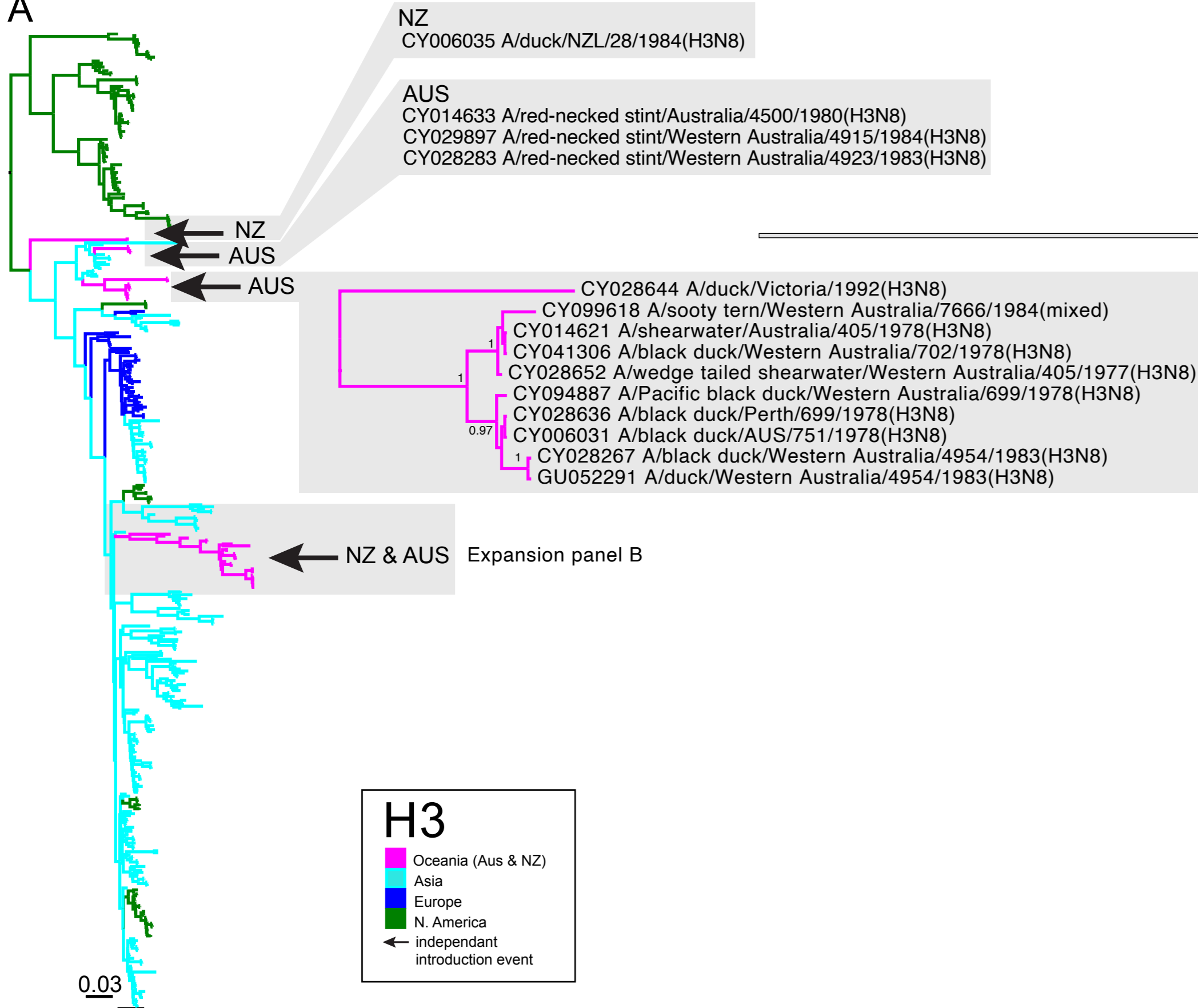

B

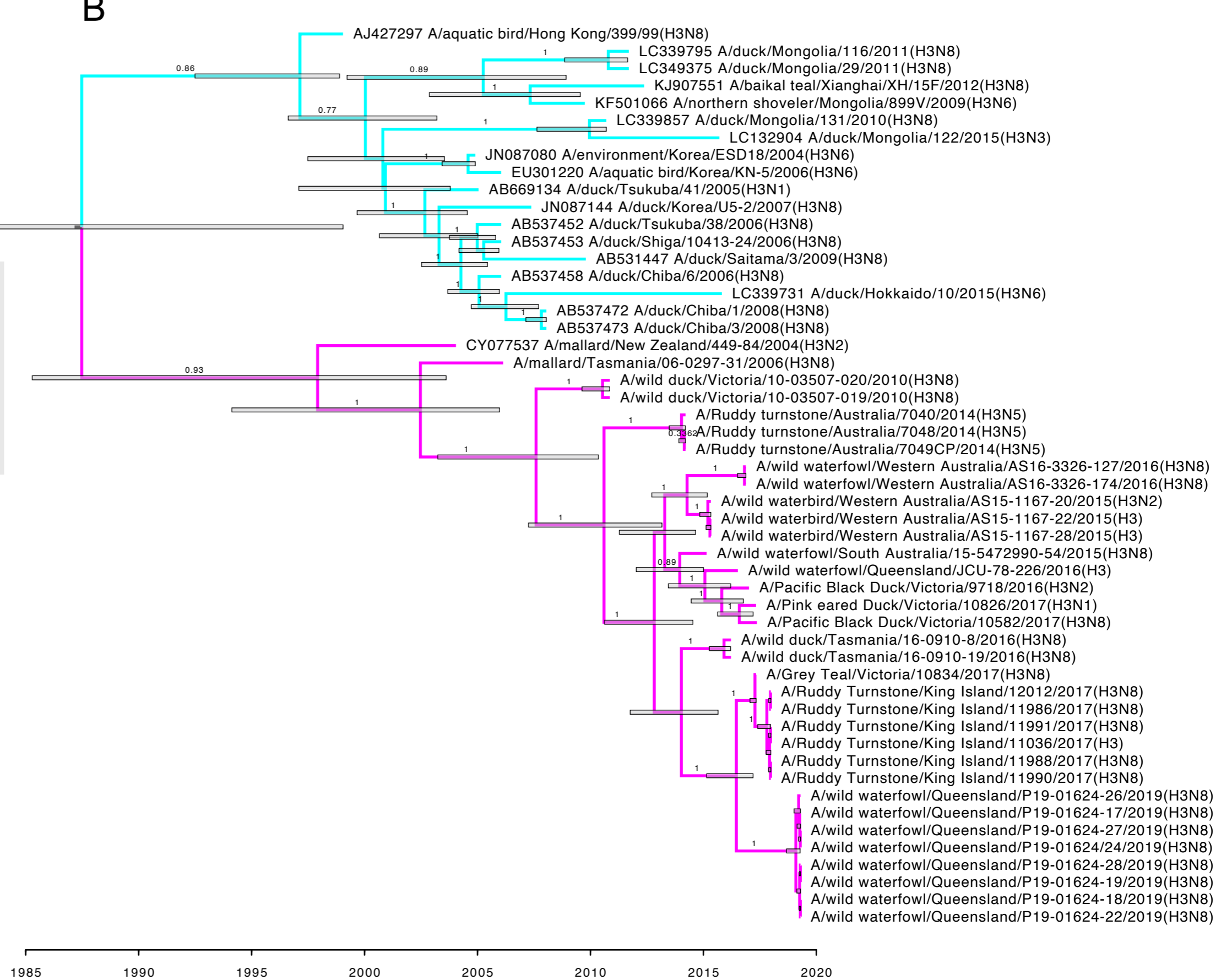

Supplement: S6 Fig — (A) Maximum likelihood tree of the sequences generated in this study, all sequences from Oceania in GenBank and reference sequences from Europe, Asia and North America. Lineages from Oceania are highlighted in grey boxes and virus names are provided. (B) Time structured phylogenetic tree comprising contemporary clades present in Australia. Node bars correspond to the 95% highest posterior density (HDP) of node height. Branches are coloured based on geography as indicated on the legend. (PDF) [file ppat.1010150.s006.pdf]

Expansion panel B

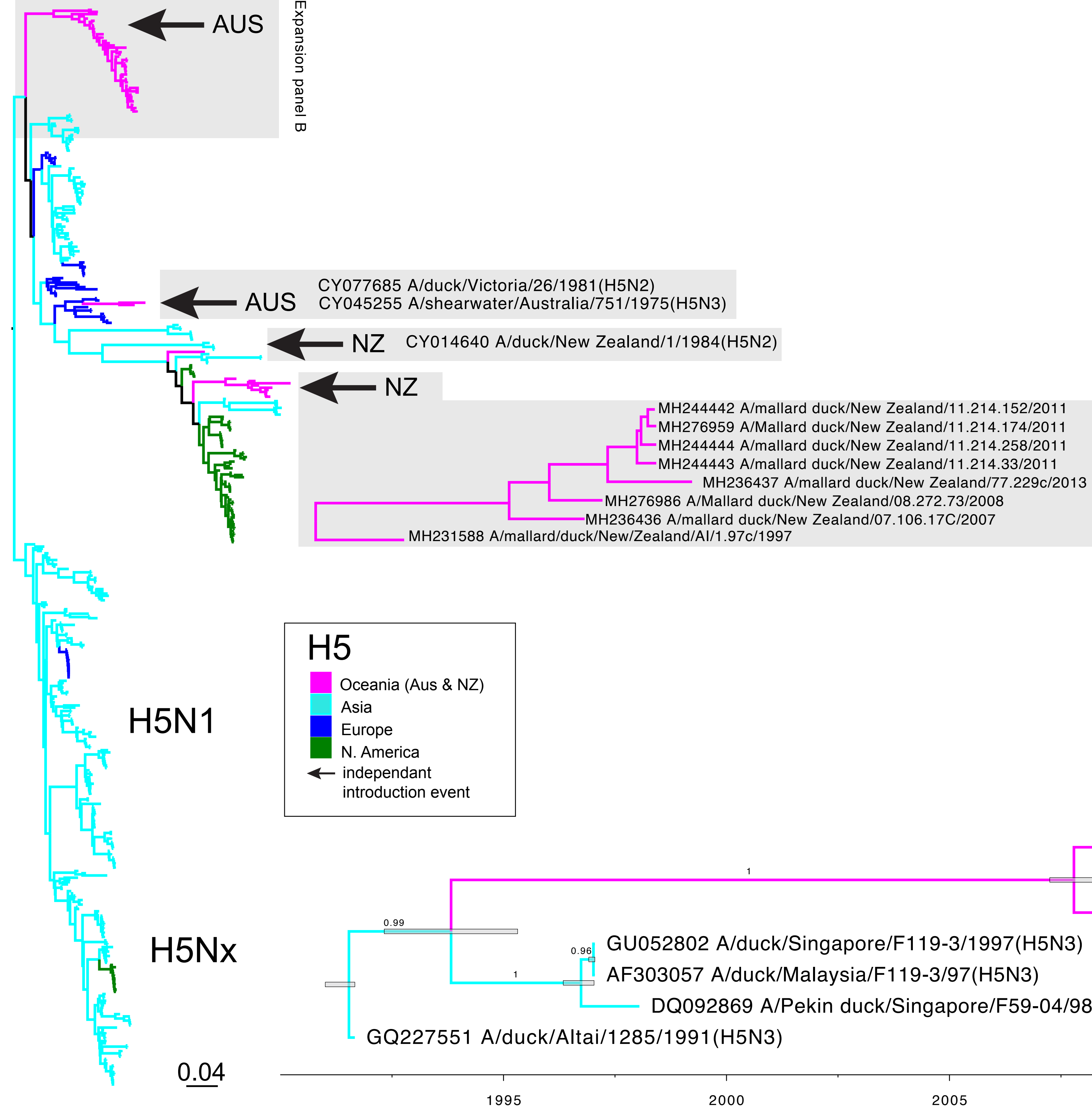

B

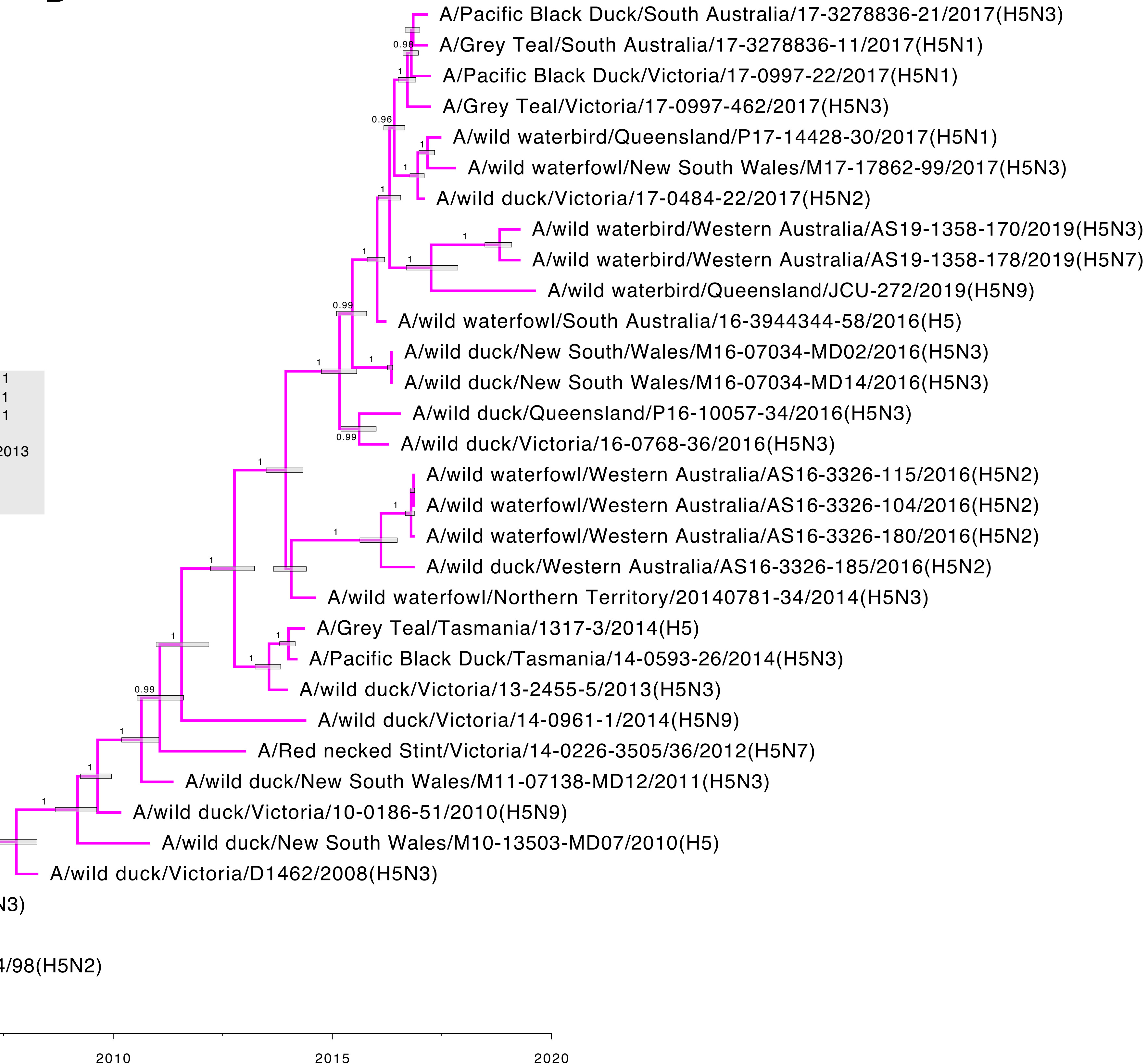

Supplement: S7 Fig — (A) Maximum likelihood tree of the sequences generated in this study, all sequences from Oceania in GenBank and reference sequences from Europe, Asia and North America. Lineages from Oceania are highlighted in grey boxes and virus names are provided. (B) Time structured phylogenetic tree comprising contemporary clades present in Australia. Node bars correspond to the 95% highest posterior density (HDP) of node height. Branches are coloured based on geography as indicated on the legend (PDF) [file ppat.1010150.s007.pdf]

A

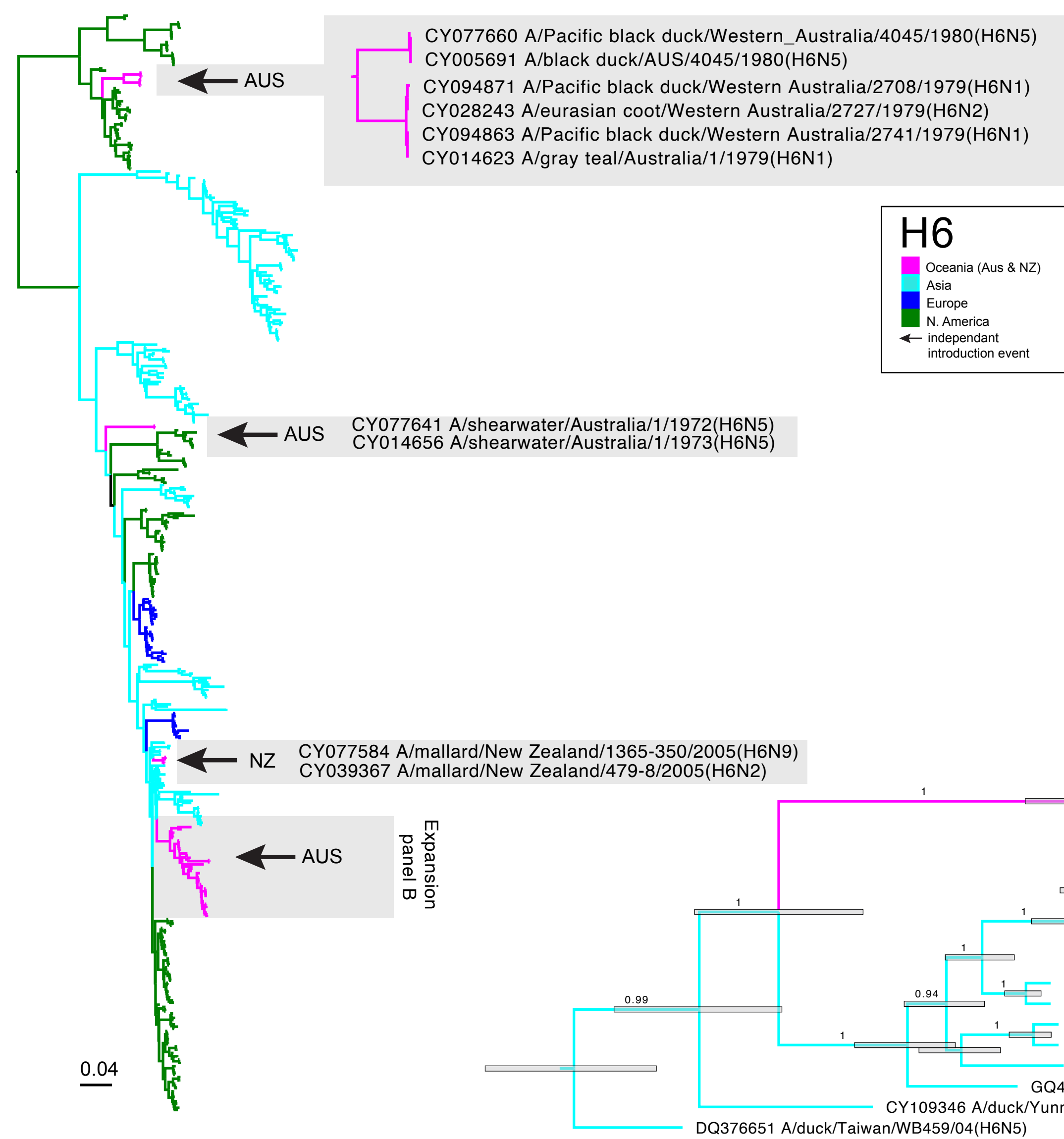

B

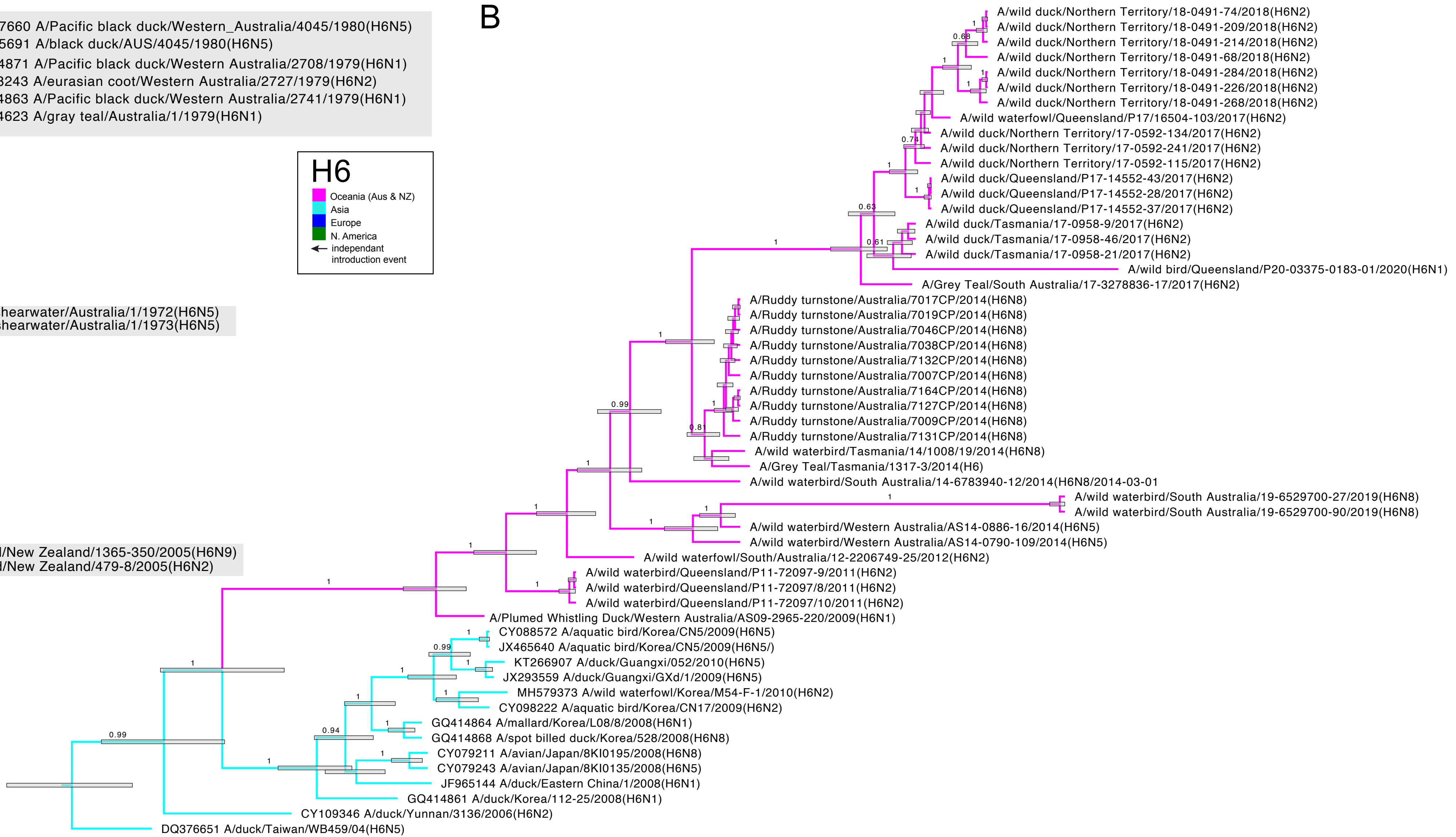

Supplement: S8 Fig — (A) Maximum likelihood tree of the sequences generated in this study, all sequences from Oceania in GenBank and reference sequences from Europe, Asia and North America. Lineages from Oceania are highlighted in grey boxes and virus names are provided. (B) Time structured phylogenetic tree comprising contemporary clades present in Australia. Node bars correspond to the 95% highest posterior density (HDP) of node height. Branches are coloured based on geography as indicated on the legend (PDF) [file ppat.1010150.s008.pdf]

A

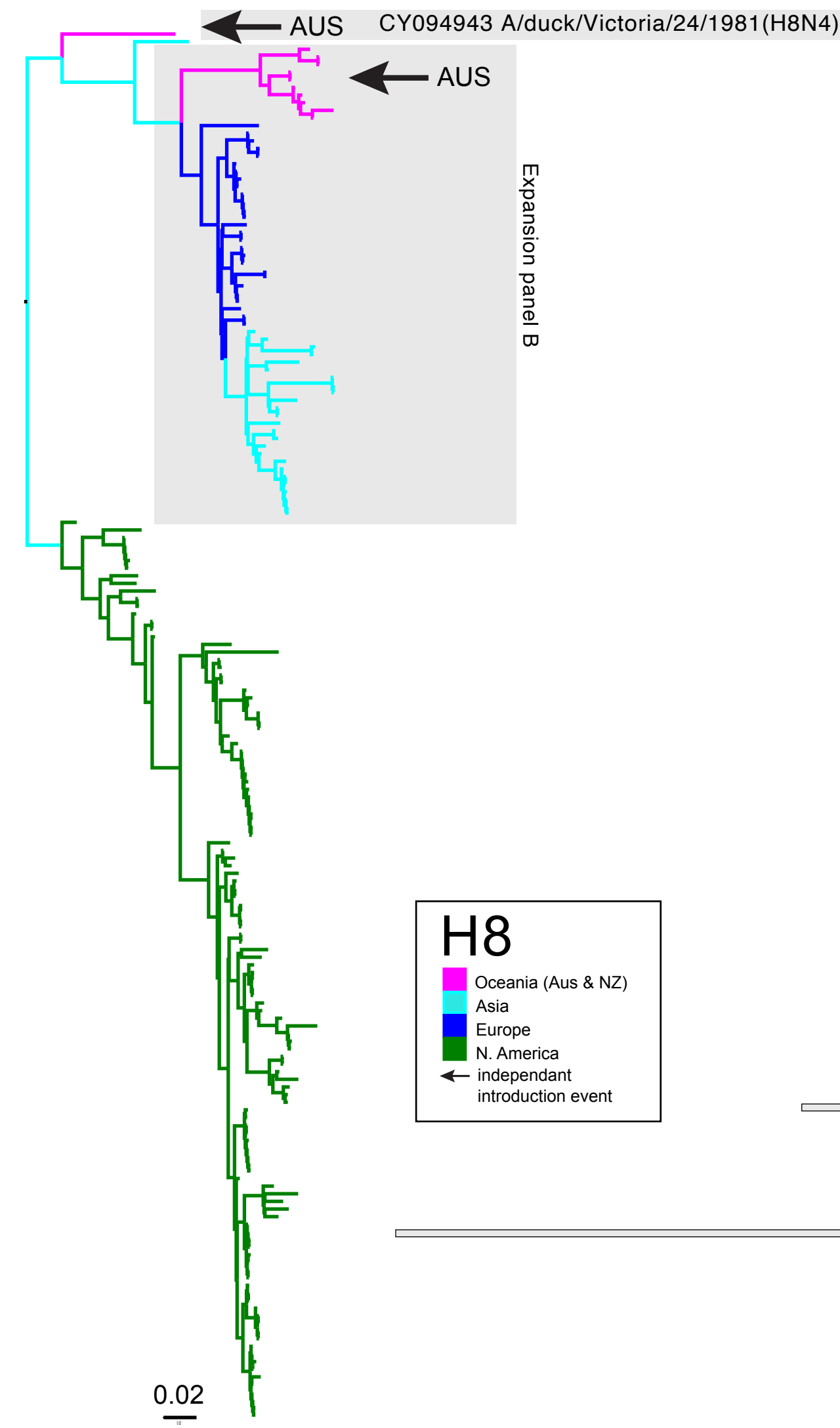

B

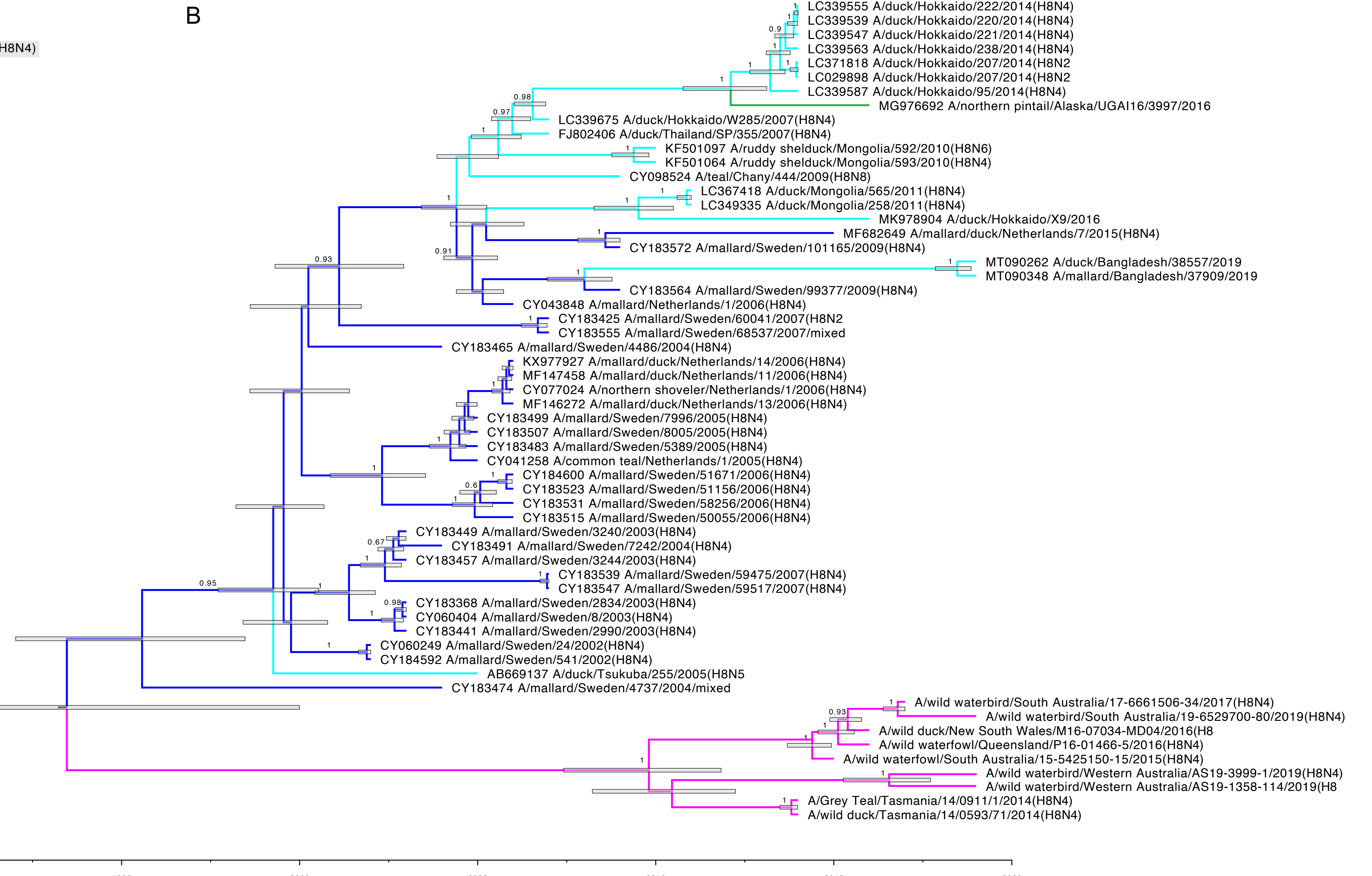

Supplement: S10 Fig — (A) Maximum likelihood tree of the sequences generated in this study, all sequences from Oceania in GenBank and reference sequences from Europe, Asia and North America. Lineages from Oceania are highlighted in grey boxes and virus names are provided. (B) Time structured phylogenetic tree comprising contemporary clades present in Australia. Node bars correspond to the 95% highest posterior density (HDP) of node height. Branches are coloured based on geography as indicated on the legend (PDF) [file ppat.1010150.s010.pdf]

A

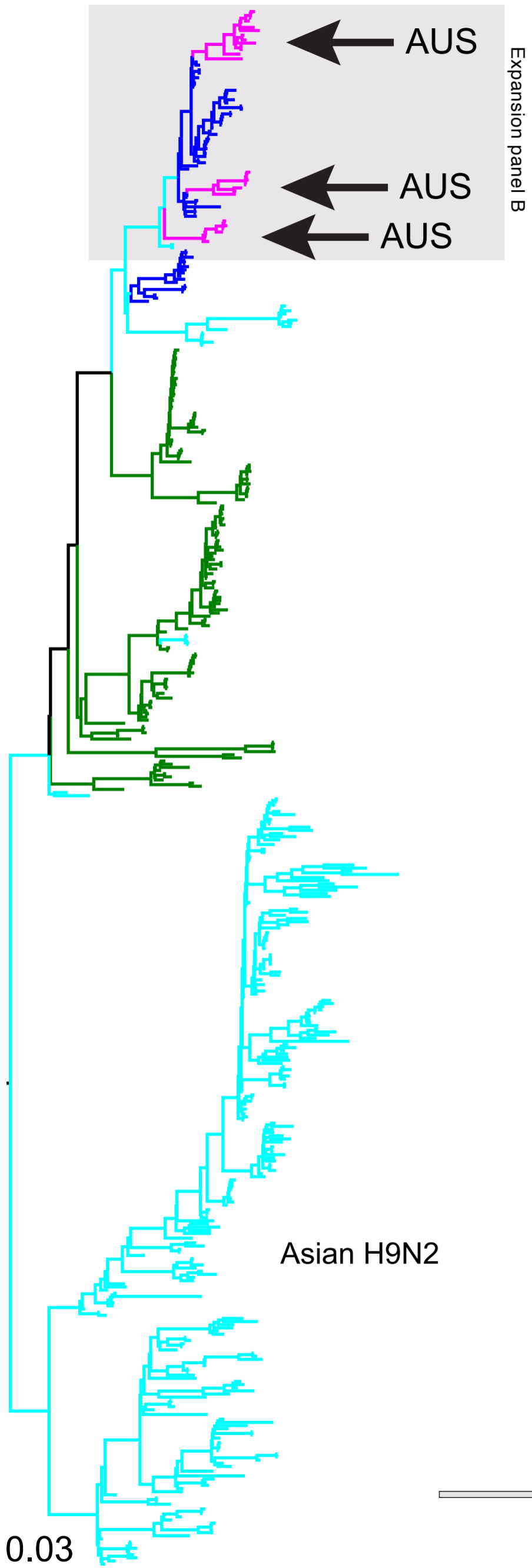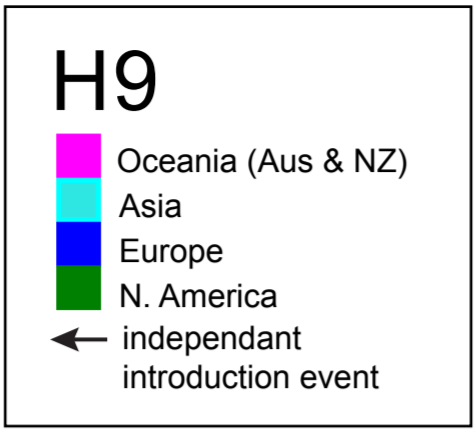

B

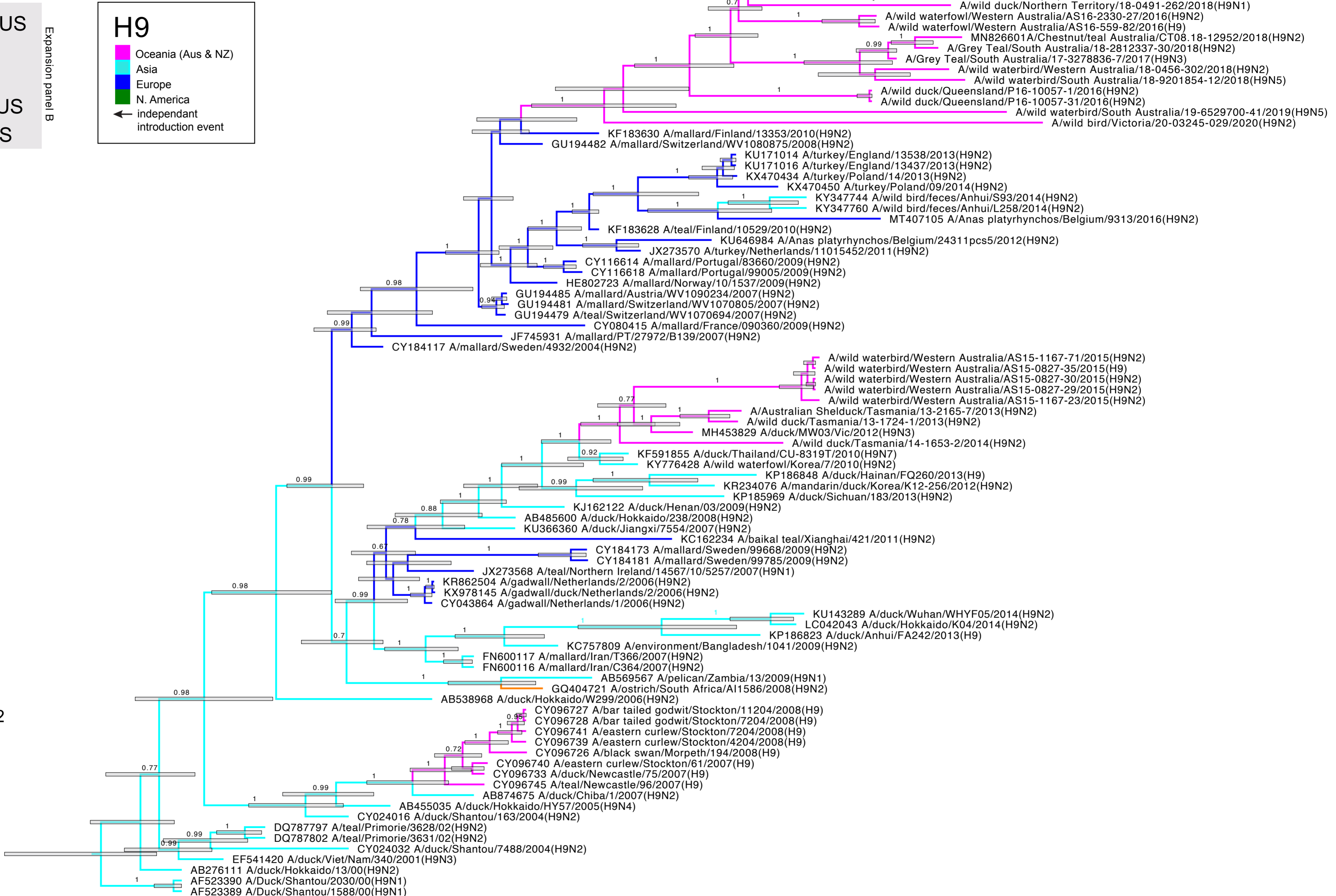

Supplement: S11 Fig — (A) Maximum likelihood tree of the sequences generated in this study, all sequences from Oceania in GenBank and reference sequences from Europe, Asia and North America. Lineages from Oceania are highlighted in grey boxes and virus names are provided. (B) Time structured phylogenetic tree comprising contemporary clades present in Australia. Node bars correspond to the 95% highest posterior density (HDP) of node height. Branches are coloured based on geography as indicated on the legend (PDF) [file ppat.1010150.s011.pdf]

A

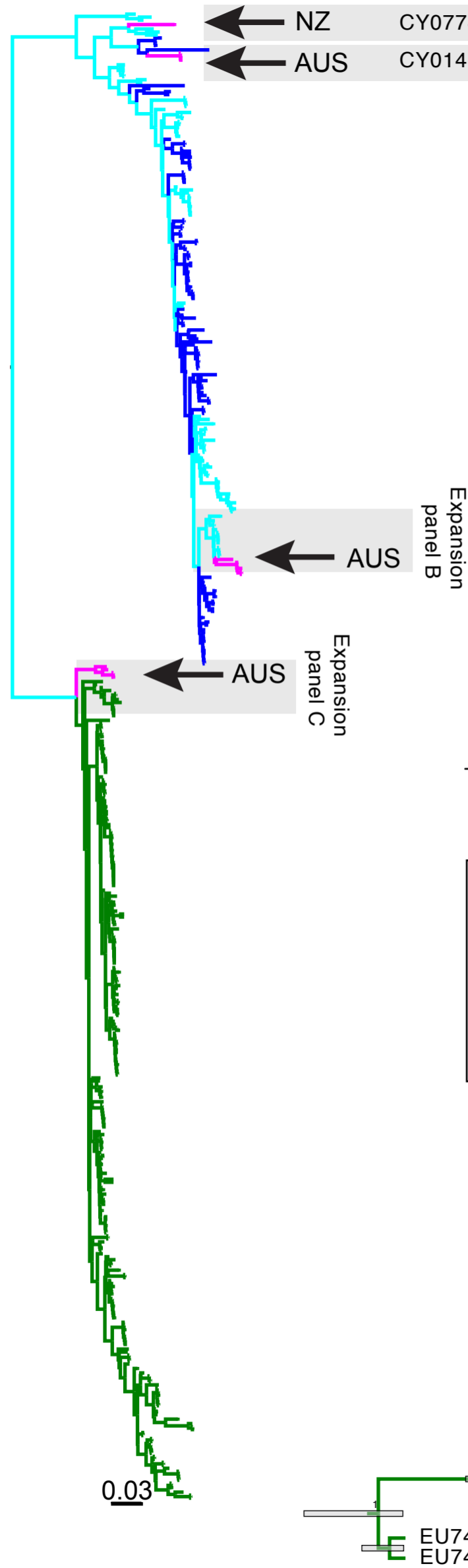

B

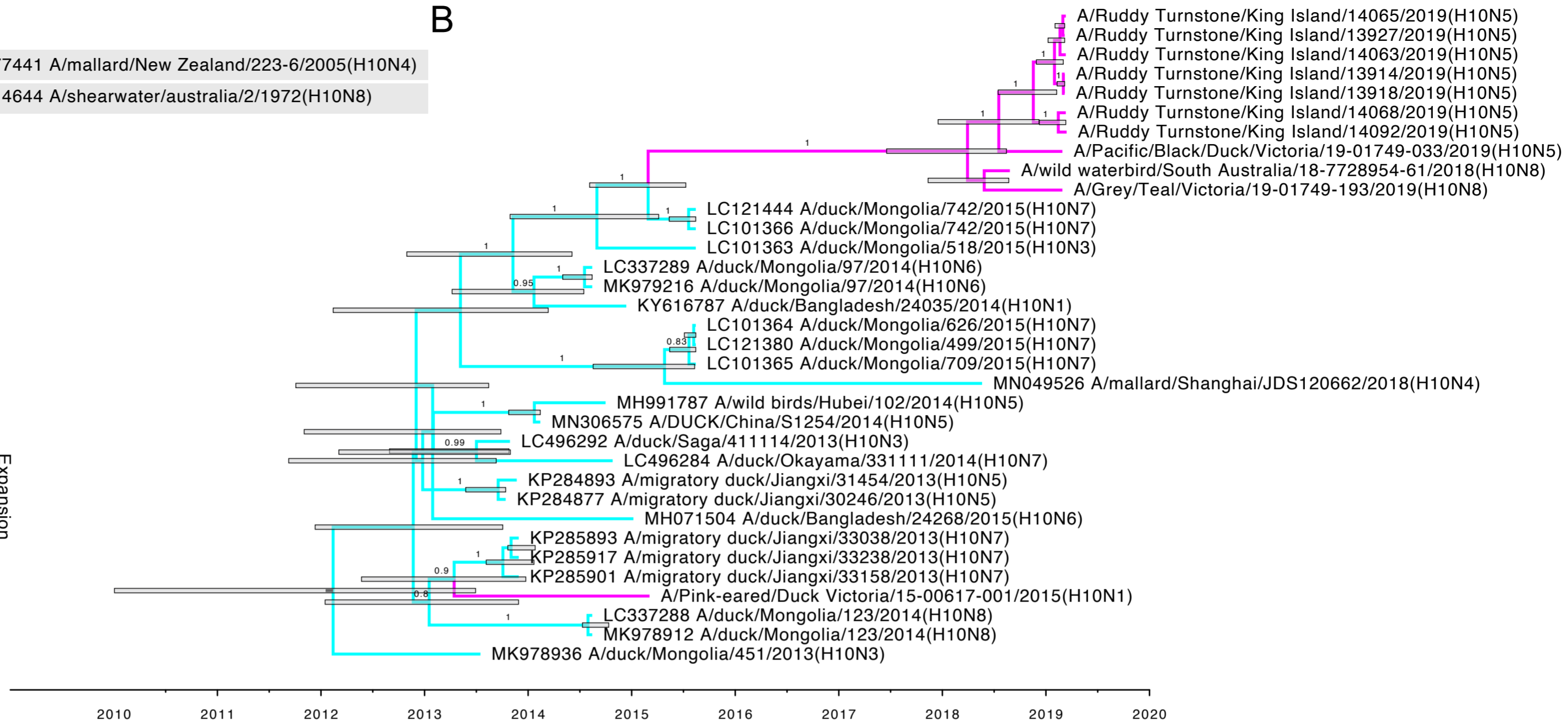

C

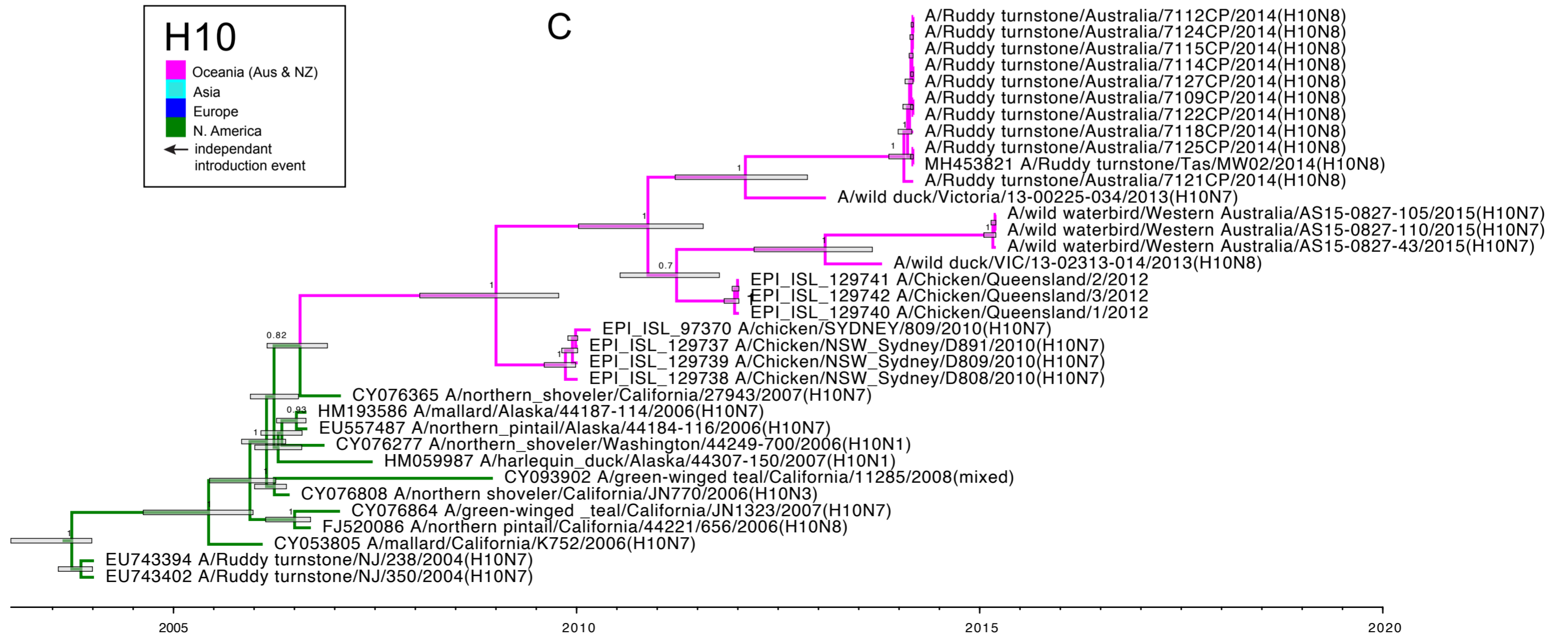

Supplement: S12 Fig — (A) Maximum likelihood tree of the sequences generated in this study, all sequences from Oceania in GenBank and reference sequences from Europe, Asia and North America. Lineages from Oceania are highlighted in grey boxes and virus names are provided. (B,C) Time structured phylogenetic tree comprising contemporary clades present in Australia. Node bars correspond to the 95% highest posterior density (HDP) of node height. Branches are coloured based on geography as indicated on the legend (PDF) [file ppat.1010150.s012.pdf]

A

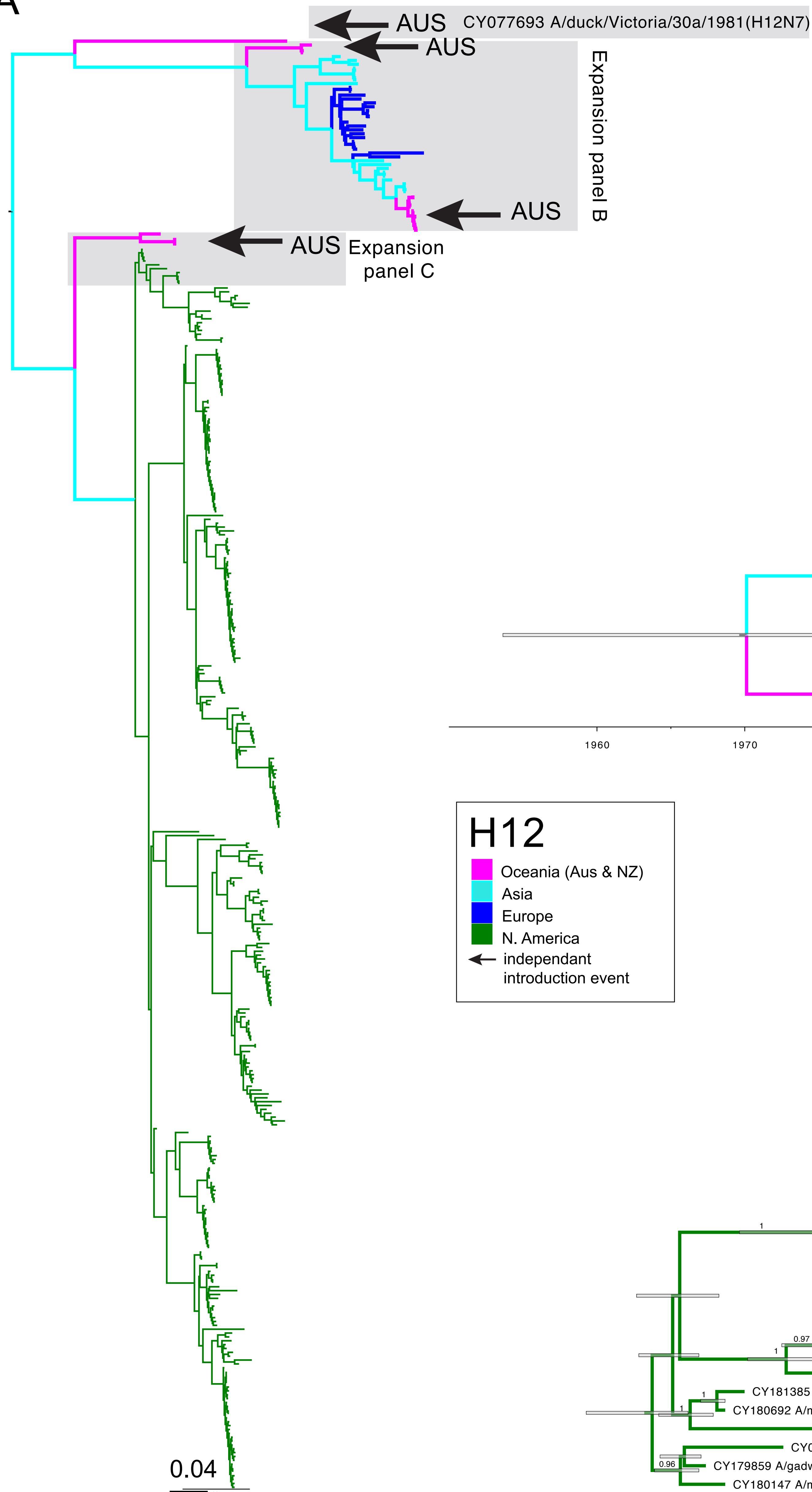

B

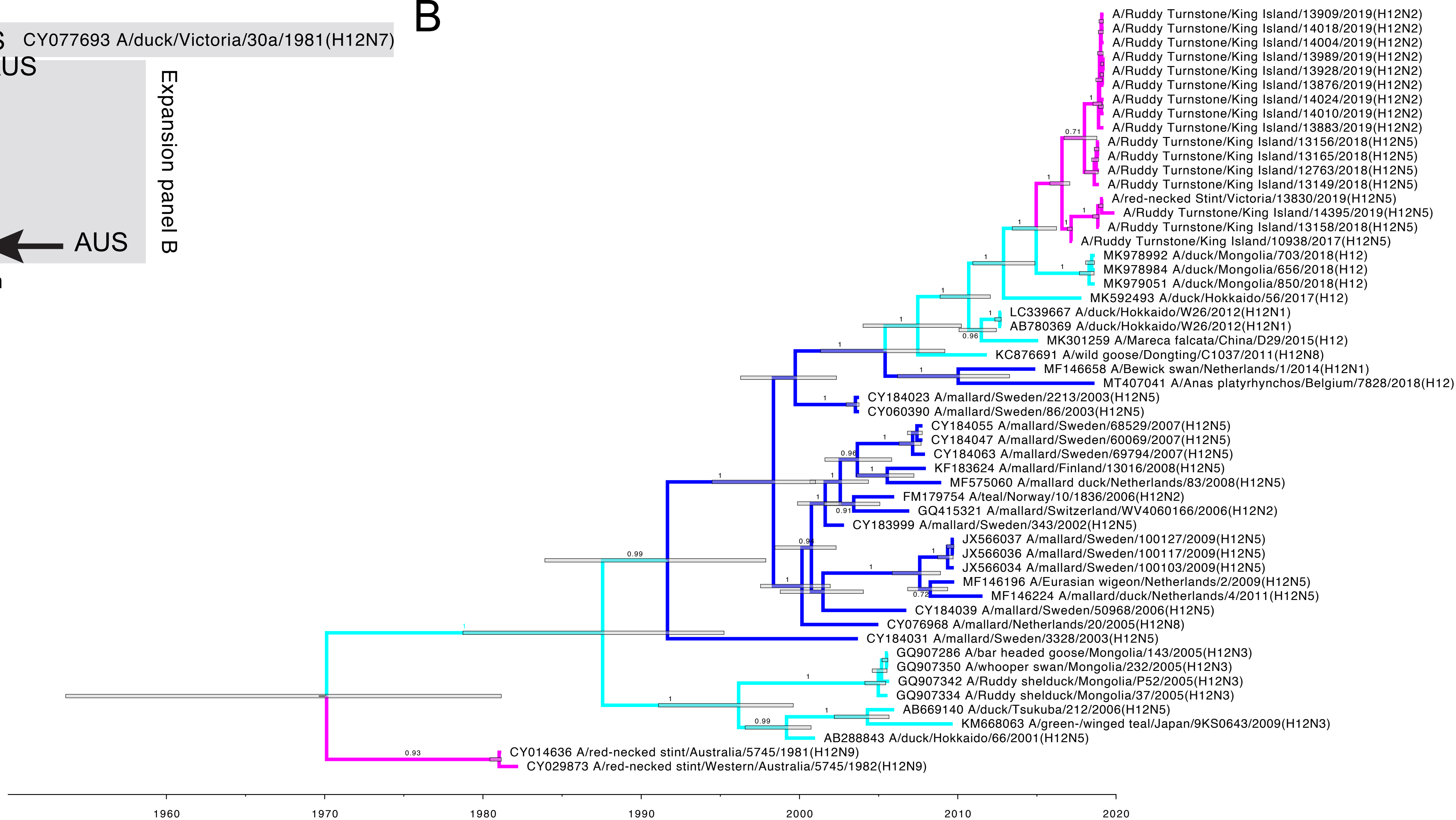

C

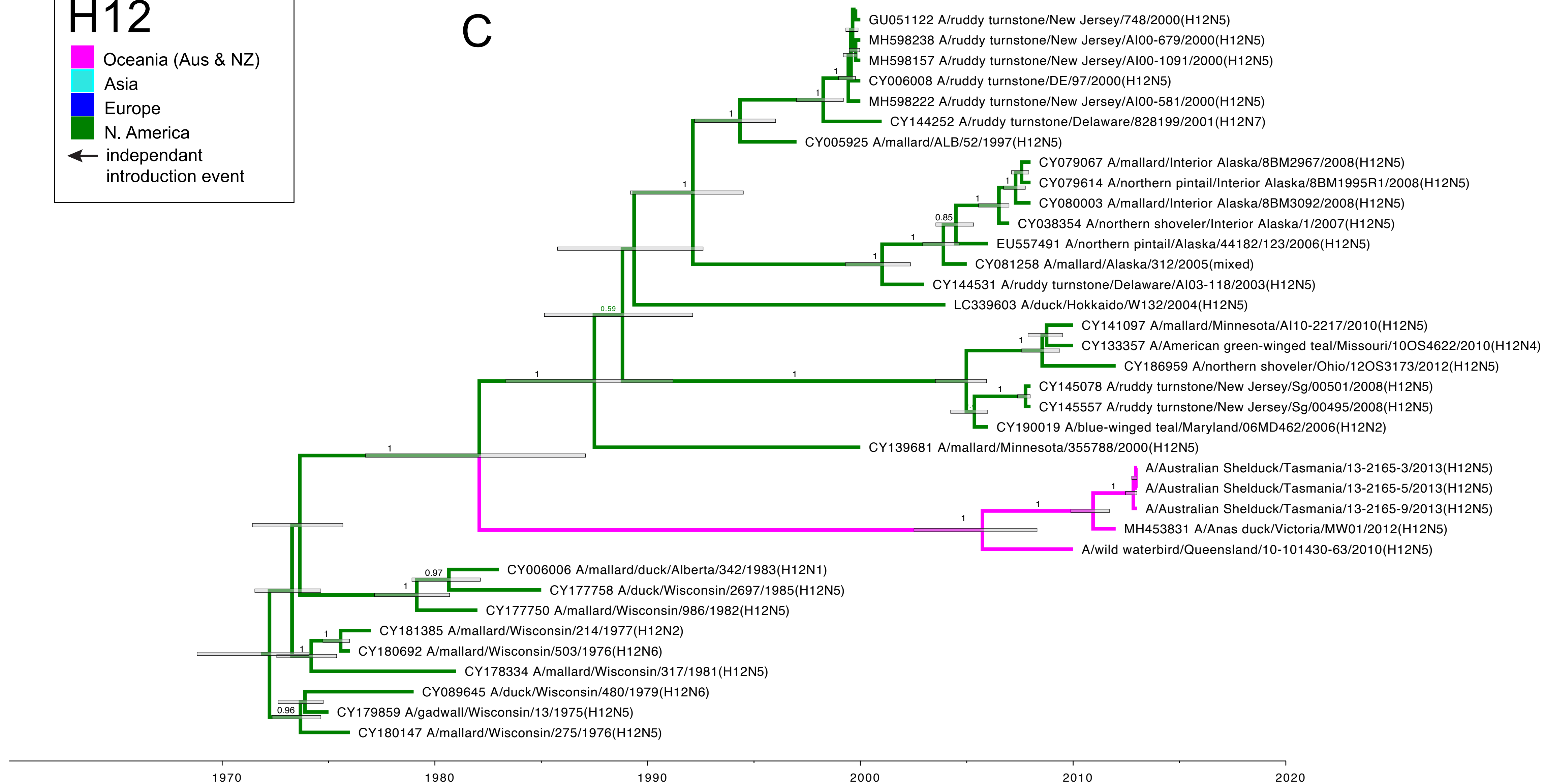

Supplement: S13 Fig — (A) Maximum likelihood tree of the sequences generated in this study, all sequences from Oceania in GenBank and reference sequences from Europe, Asia and North America. Lineages from Oceania are highlighted in grey boxes and virus names are provided. (B,C) Time structured phylogenetic tree comprising contemporary clades present in Australia. Node bars correspond to the 95% highest posterior density (HDP) of node height. Branches are coloured based on geography as indicated on the legend (PDF) [file ppat.1010150.s013.pdf]

A

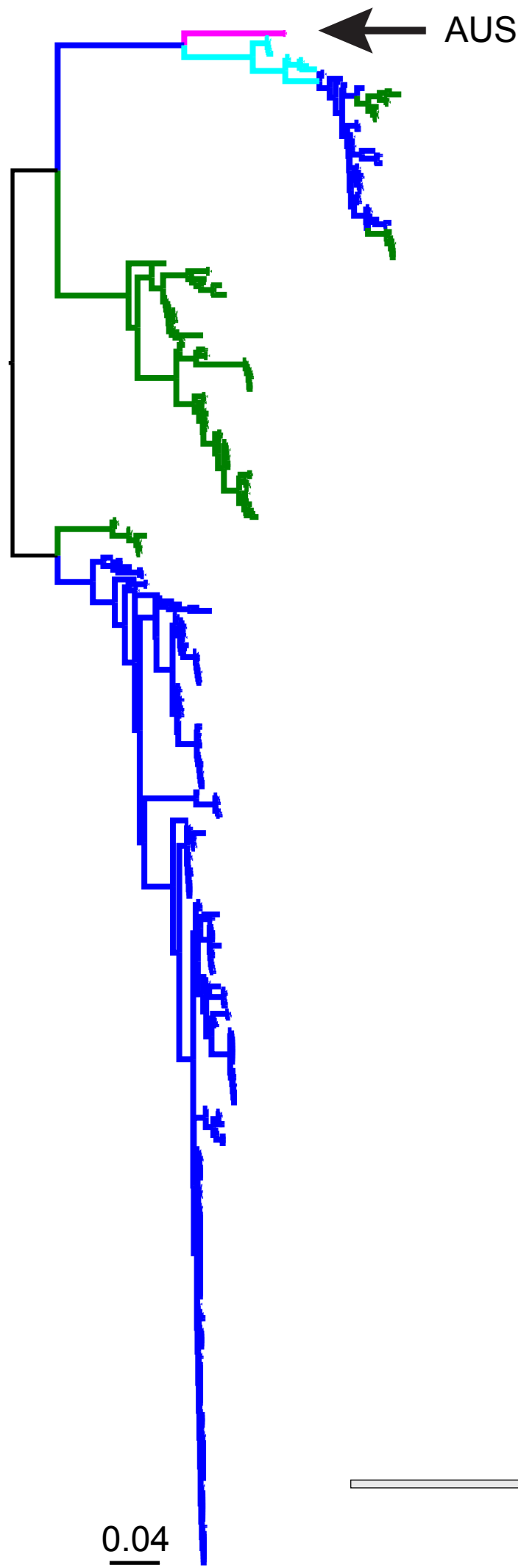

B

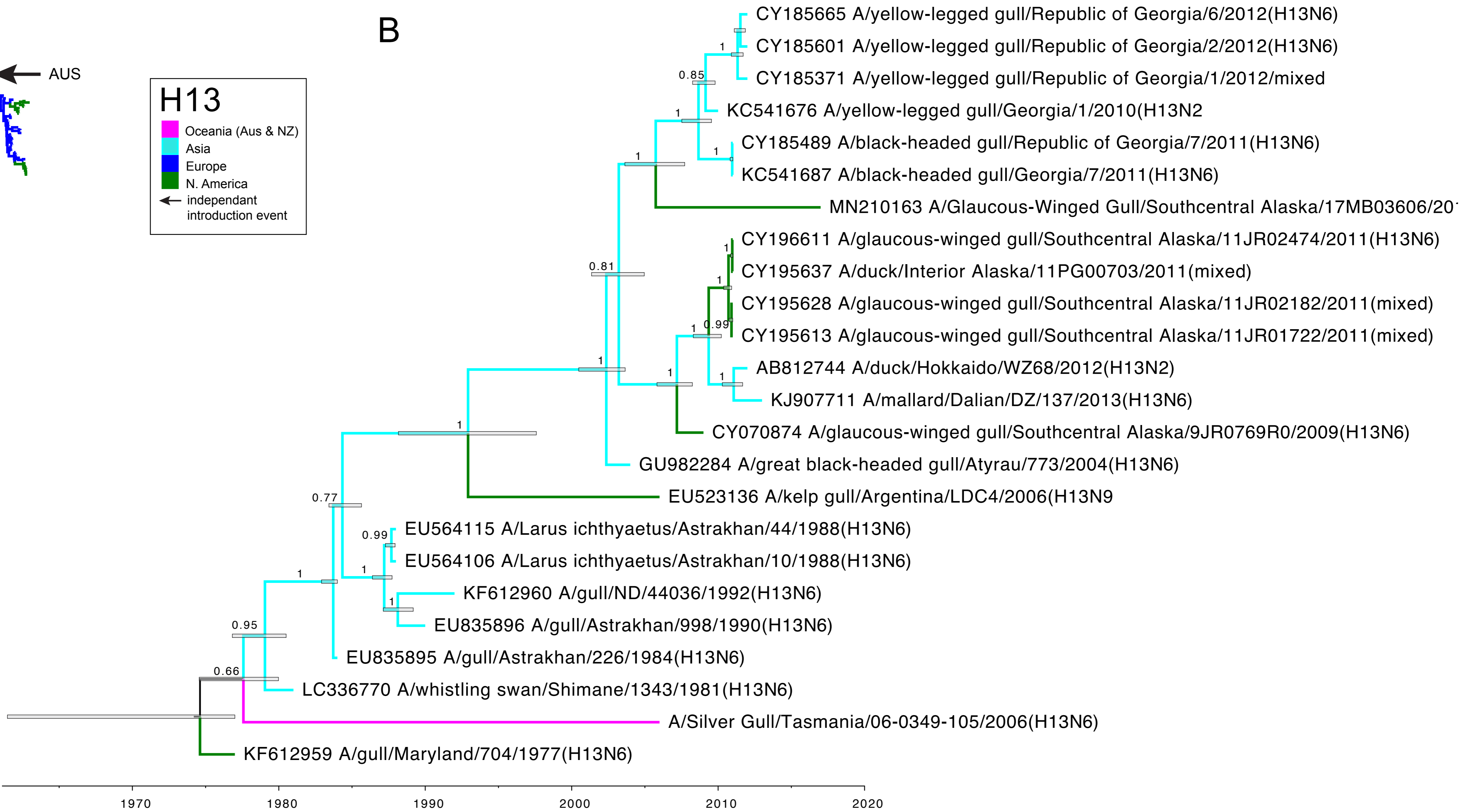

Supplement: S14 Fig — (A) Maximum likelihood tree of the sequences generated in this study, all sequences from Oceania in GenBank and reference sequences from Europe, Asia and North America. Lineages from Oceania are highlighted in grey boxes and virus names are provided. (B) Time structured phylogenetic tree comprising contemporary clades present in Australia. Node bars correspond to the 95% highest posterior density (HDP) of node height. Branches are coloured based on geography as indicated on the legend (PDF) [file ppat.1010150.s014.pdf]

A

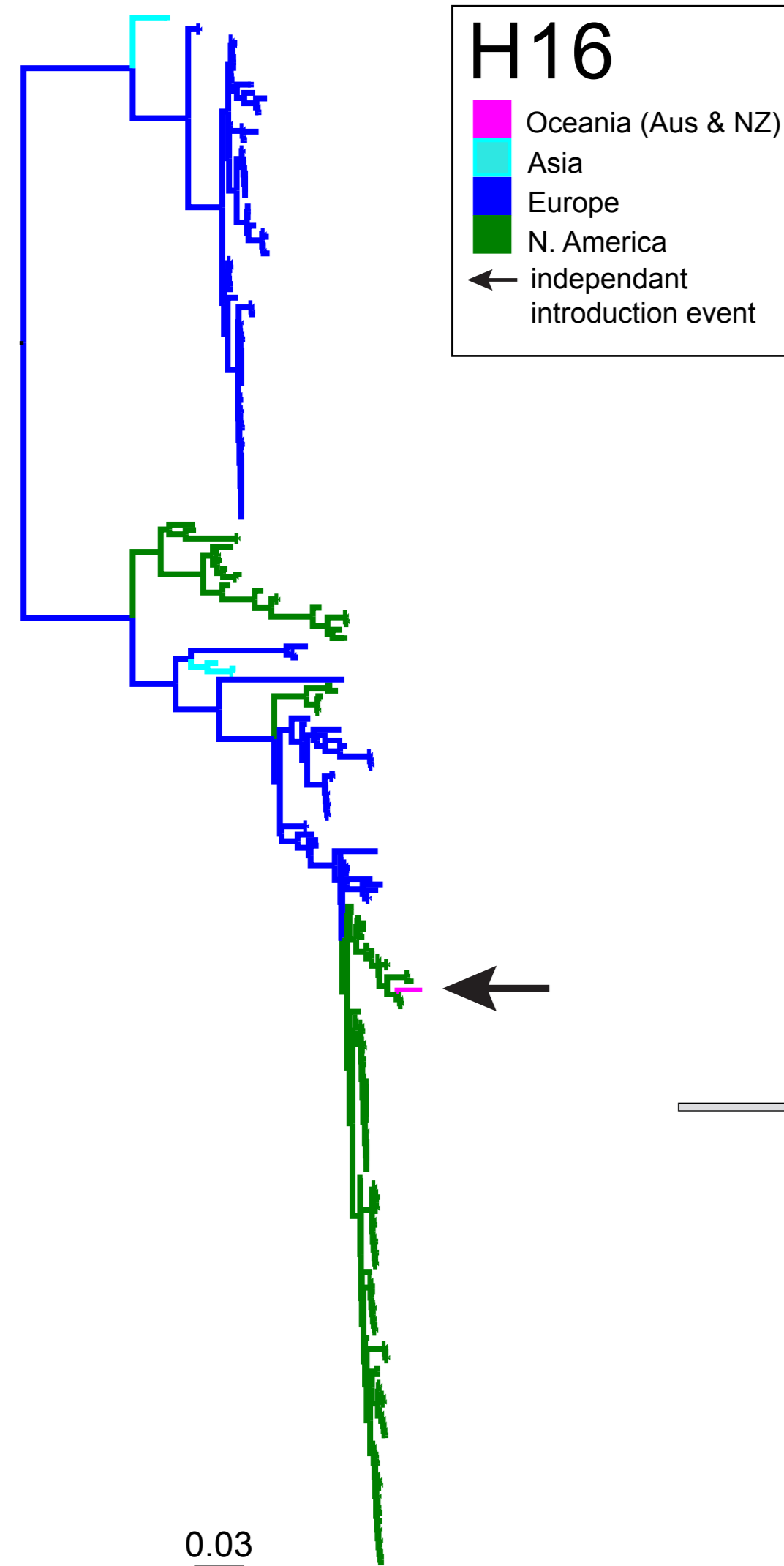

B

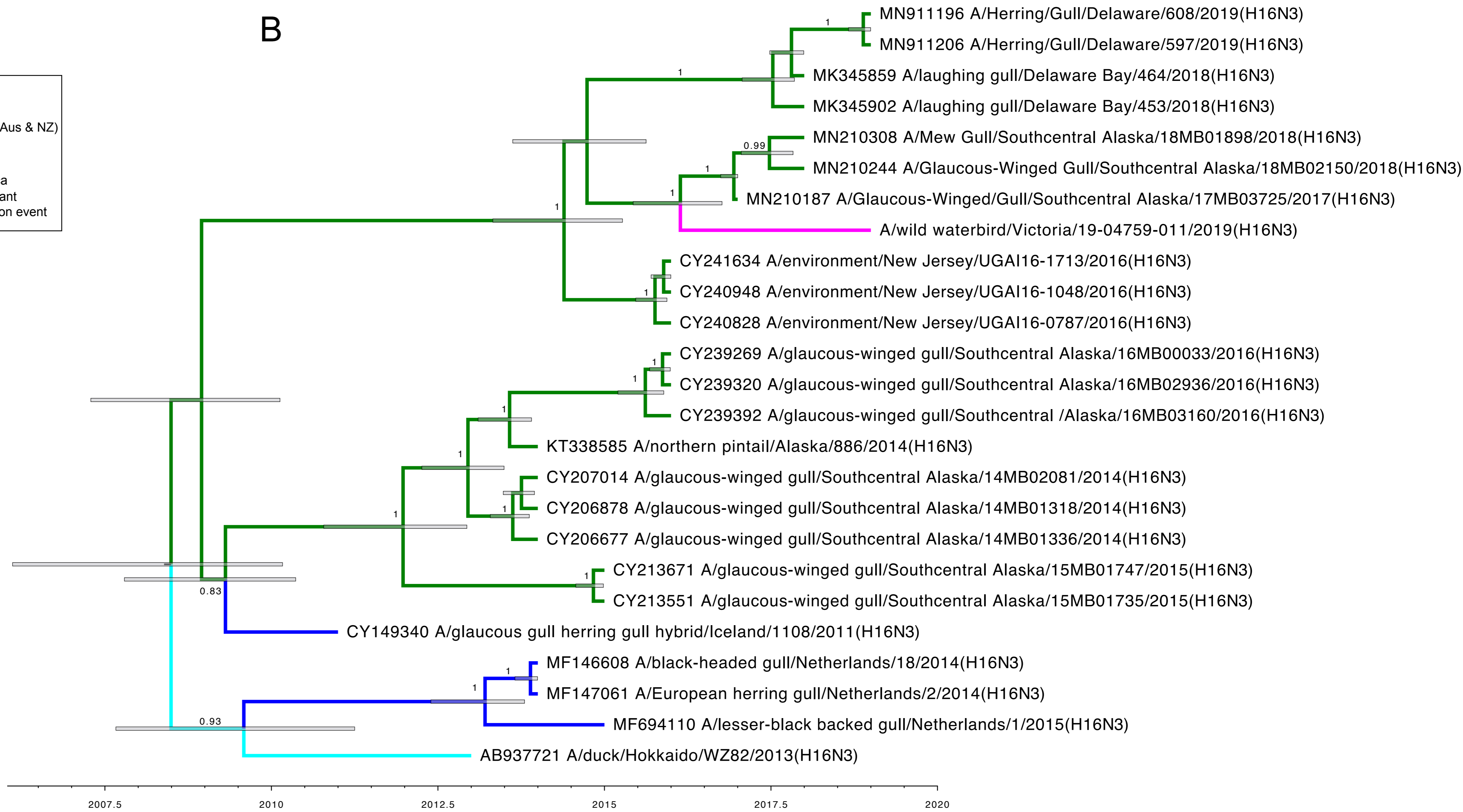

Supplement: S15 Fig — (A) Maximum likelihood tree of the sequences generated in this study, all sequences from Oceania in GenBank and reference sequences from Europe, Asia and North America. Lineages from Oceania are highlighted in grey boxes and virus names are provided. (B) Time structured phylogenetic tree comprising contemporary clades present in Australia. Node bars correspond to the 95% highest posterior density (HDP) of node height. Branches are coloured based on geography as indicated on the legend (PDF) [file ppat.1010150.s015.pdf]

A

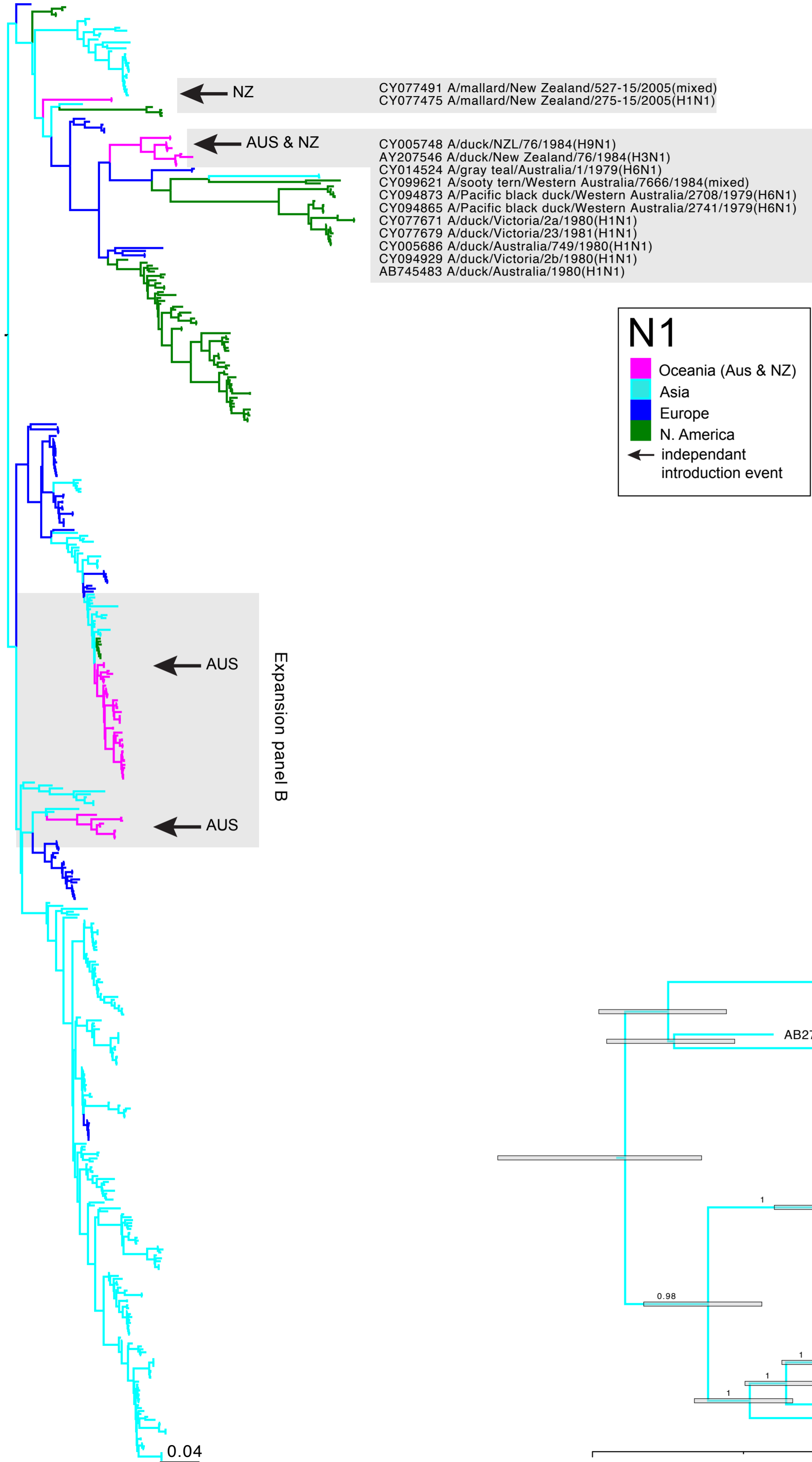

B

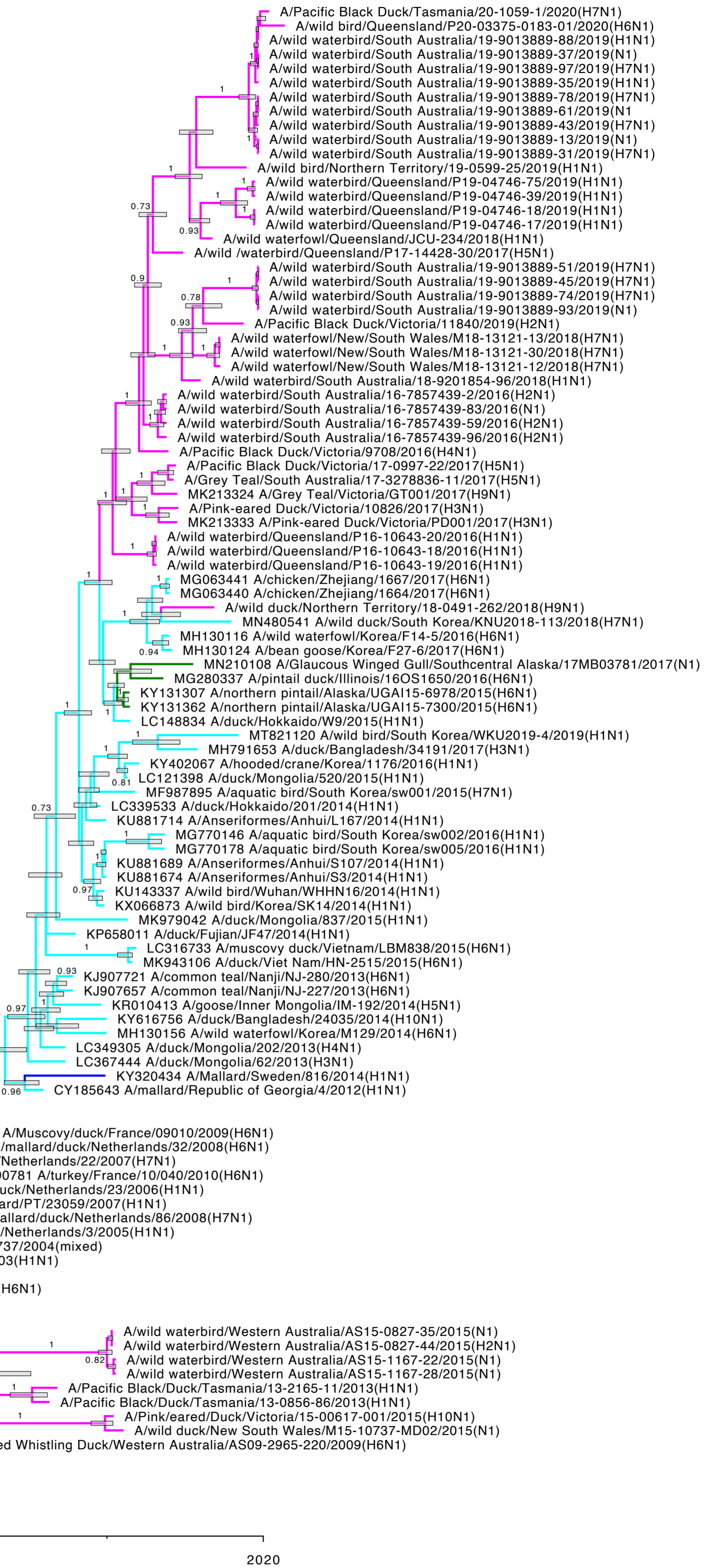

Supplement: S16 Fig — (A) Maximum likelihood tree of the sequences generated in this study, all sequences from Oceania in GenBank and reference sequences from Europe, Asia and North America. Lineages from Oceania are highlighted in grey boxes and virus names are provided. (B) Time structured phylogenetic tree comprising contemporary clades present in Australia. Node bars correspond to the 95% highest posterior density (HDP) of node height. Branches are coloured based on geography as indicated on the legend (PDF) [file ppat.1010150.s016.pdf]

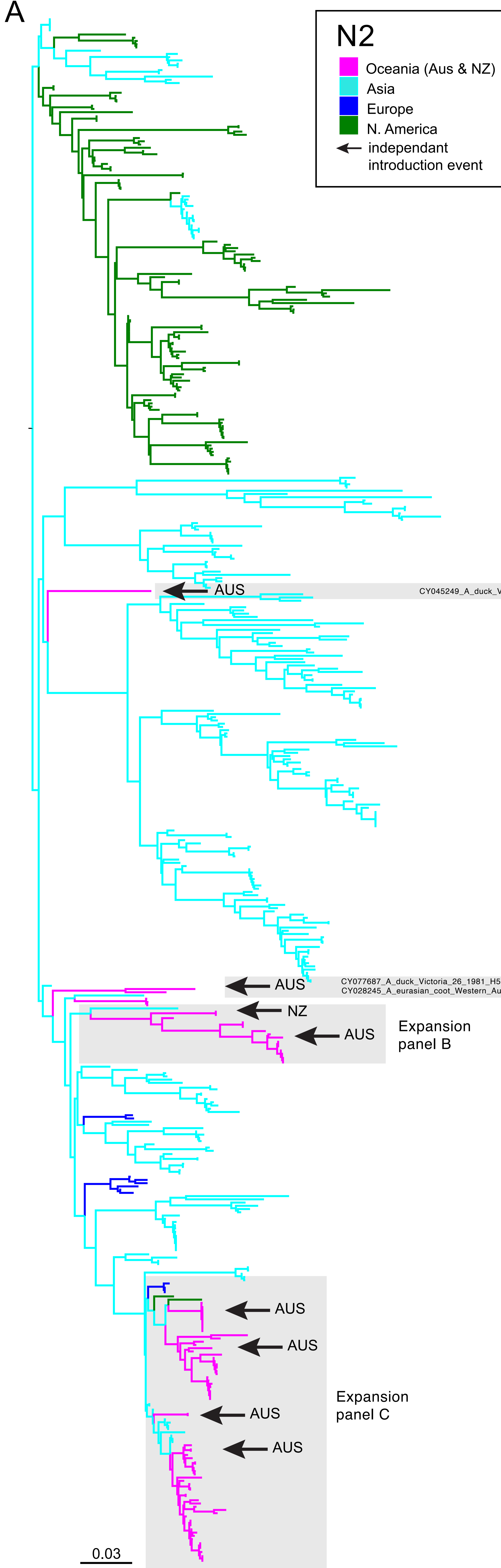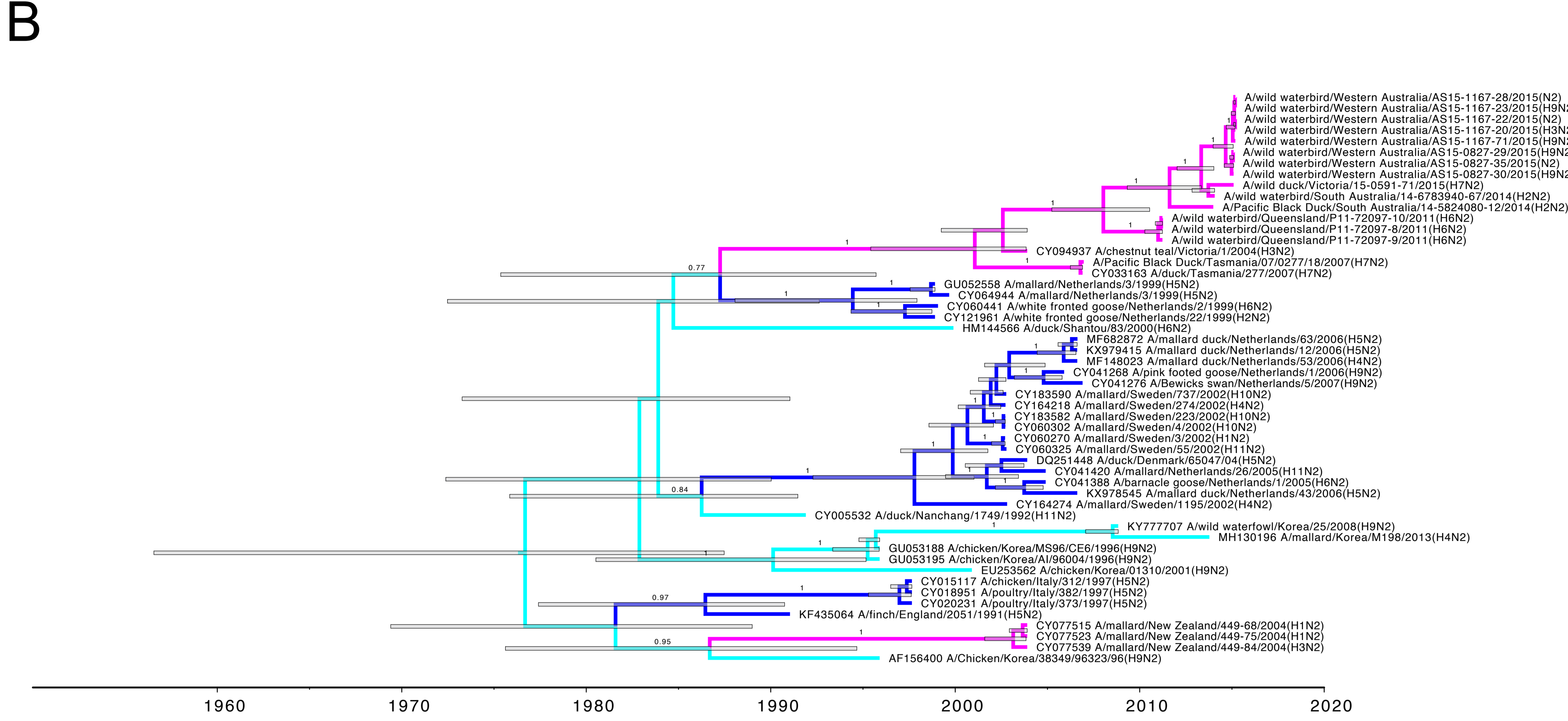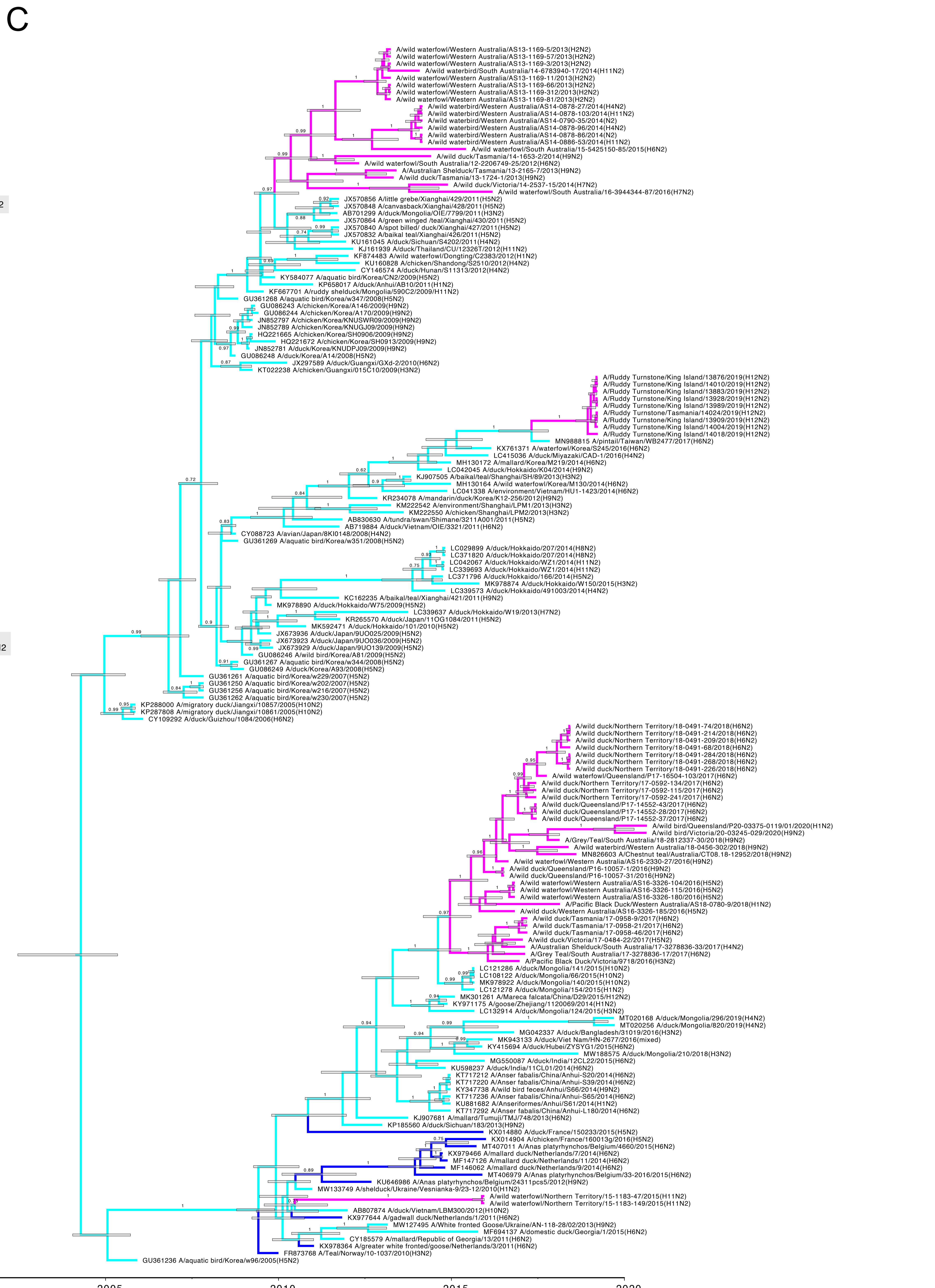

Supplement: S17 Fig — (A) Maximum likelihood tree of the sequences generated in this study, all sequences from Oceania in GenBank and reference sequences from Europe, Asia and North America. Lineages from Oceania are highlighted in grey boxes and virus names are provided. (B,C) Time structured phylogenetic tree comprising contemporary clades present in Australia. Node bars correspond to the 95% highest posterior density (HDP) of node height. Branches are coloured based on geography as indicated on the legend (PDF) [file ppat.1010150.s017.pdf]

A

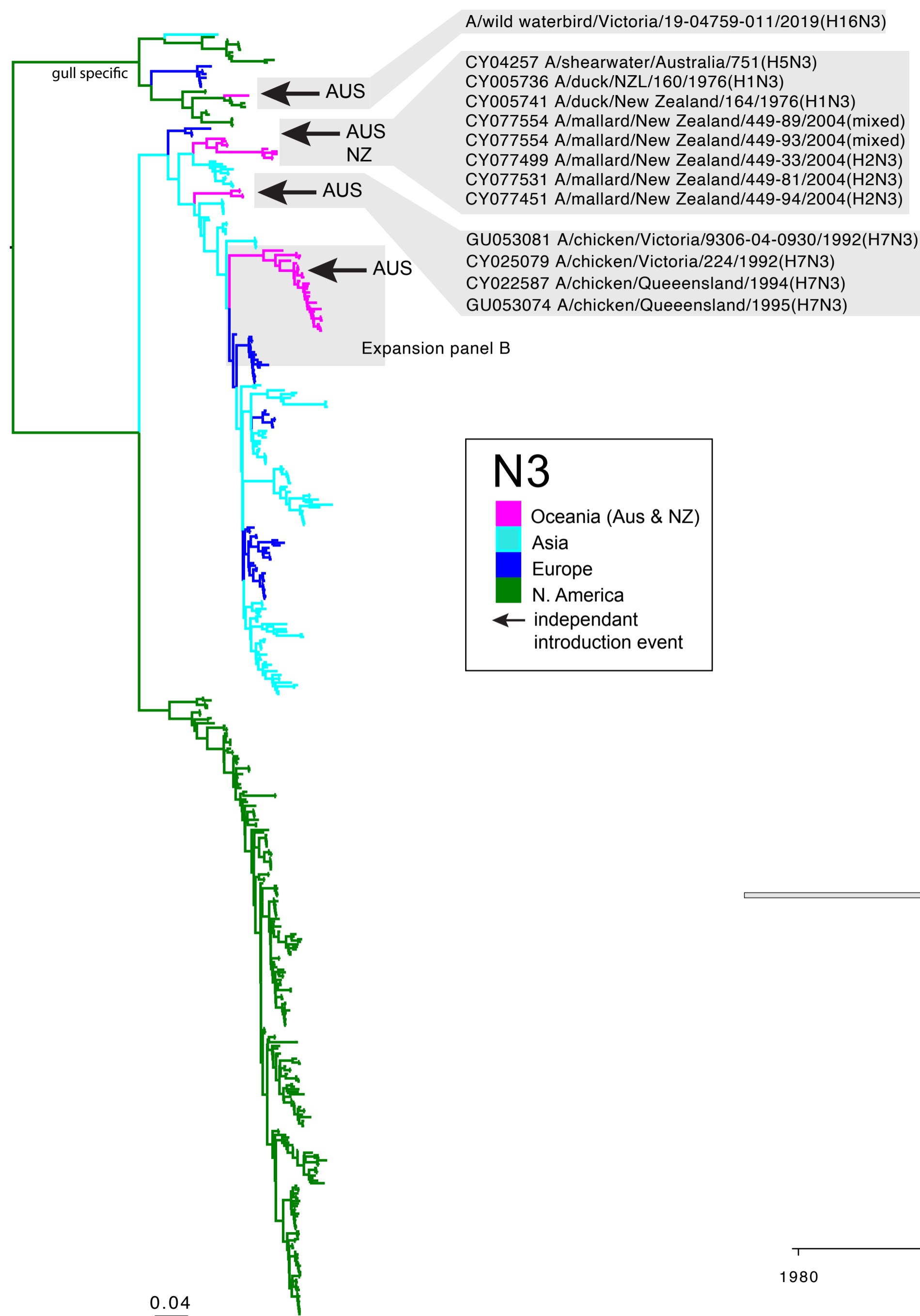

B

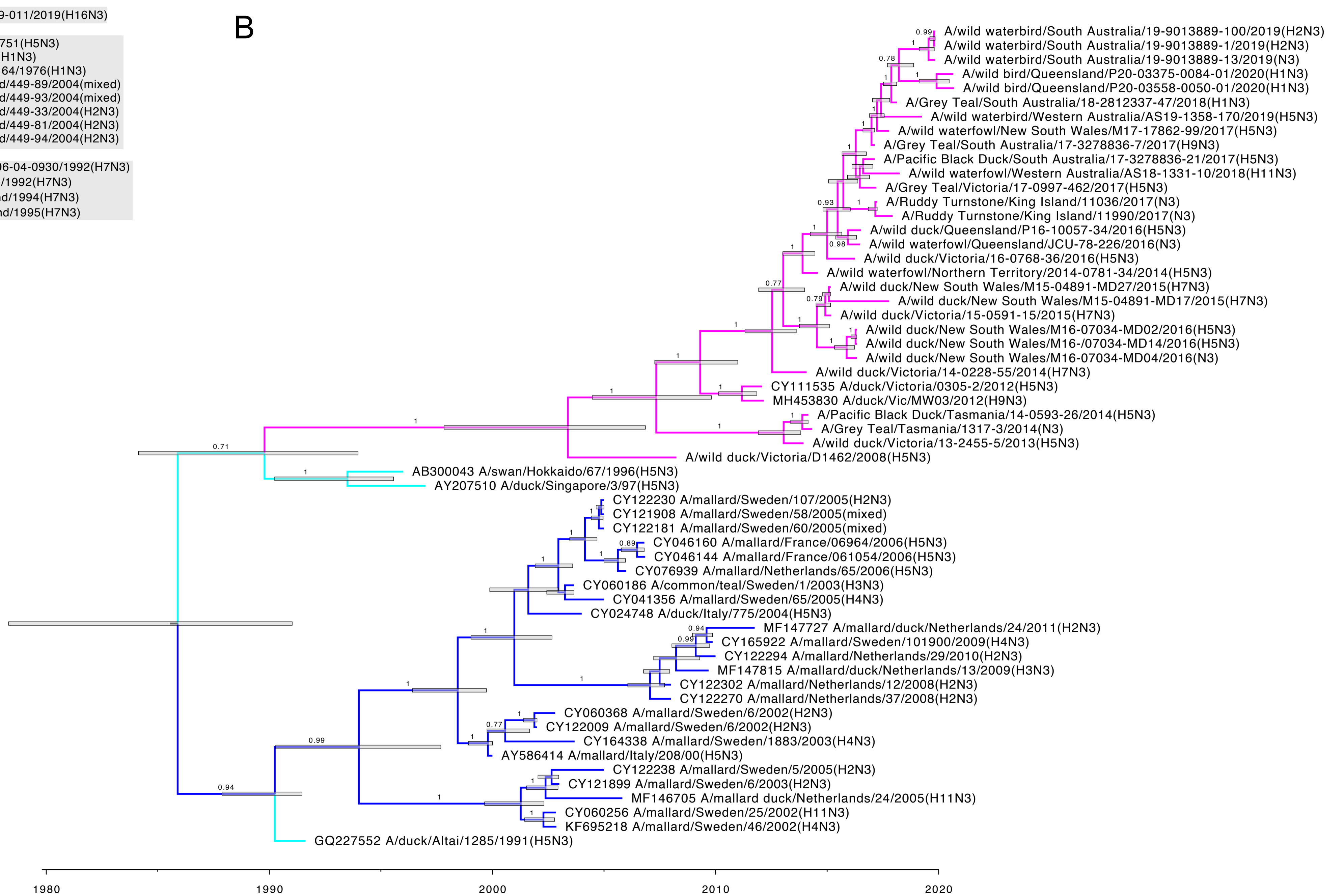

Supplement: S18 Fig — (A) Maximum likelihood tree of the sequences generated in this study, all sequences from Oceania in GenBank and reference sequences from Europe, Asia and North America. Lineages from Oceania are highlighted in grey boxes and virus names are provided. (B) Time structured phylogenetic tree comprising contemporary clades present in Australia. Node bars correspond to the 95% highest posterior density (HDP) of node height. Branches are coloured based on geography as indicated on the legend (PDF) [file ppat.1010150.s018.pdf]

MK978866 A/duck/Hokkaido/18/2000(H10N4)

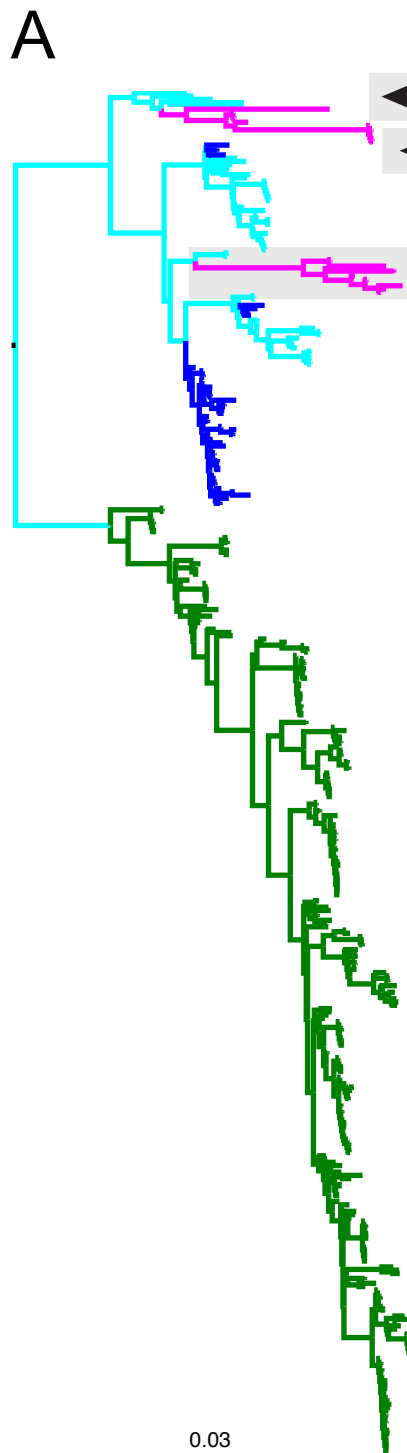

Supplement: S19 Fig — (A) Maximum likelihood tree of the sequences generated in this study, all sequences from Oceania in GenBank and reference sequences from Europe, Asia and North America. Lineages from Oceania are highlighted in grey boxes and virus names are provided. (B) Time structured phylogenetic tree comprising contemporary clades present in Australia. Node bars correspond to the 95% highest posterior density (HDP) of node height. Branches are coloured based on geography as indicated on the legend (PDF) [file ppat.1010150.s019.pdf]

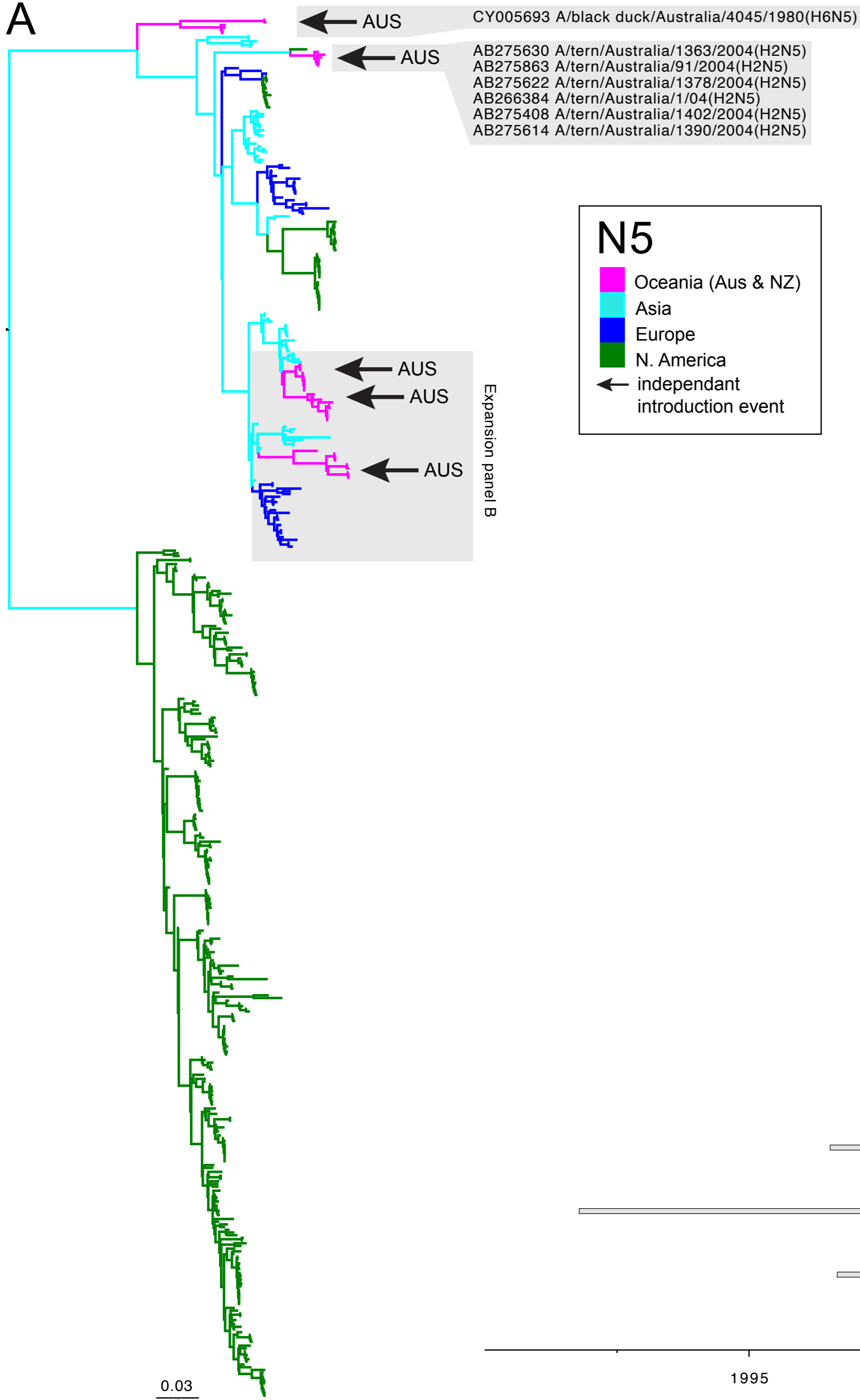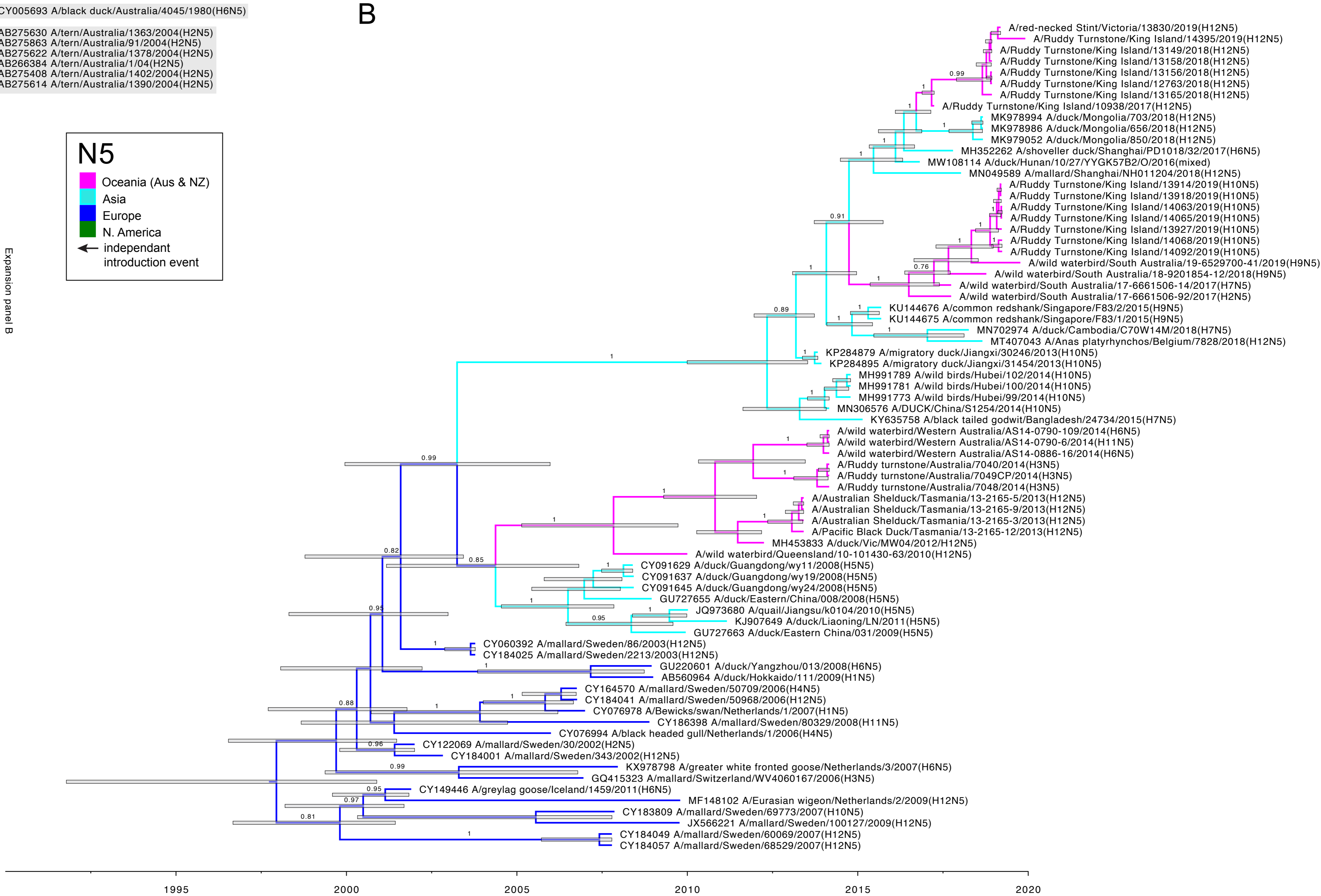

Supplement: S20 Fig — (A) Maximum likelihood tree of the sequences generated in this study, all sequences from Oceania in GenBank and reference sequences from Europe, Asia and North America. Lineages from Oceania are highlighted in grey boxes and virus names are provided. (B) Time structured phylogenetic tree comprising contemporary clades present in Australia. Node bars correspond to the 95% highest posterior density (HDP) of node height. Branches are coloured based on geography as indicated on the legend (PDF) [file ppat.1010150.s020.pdf]

A

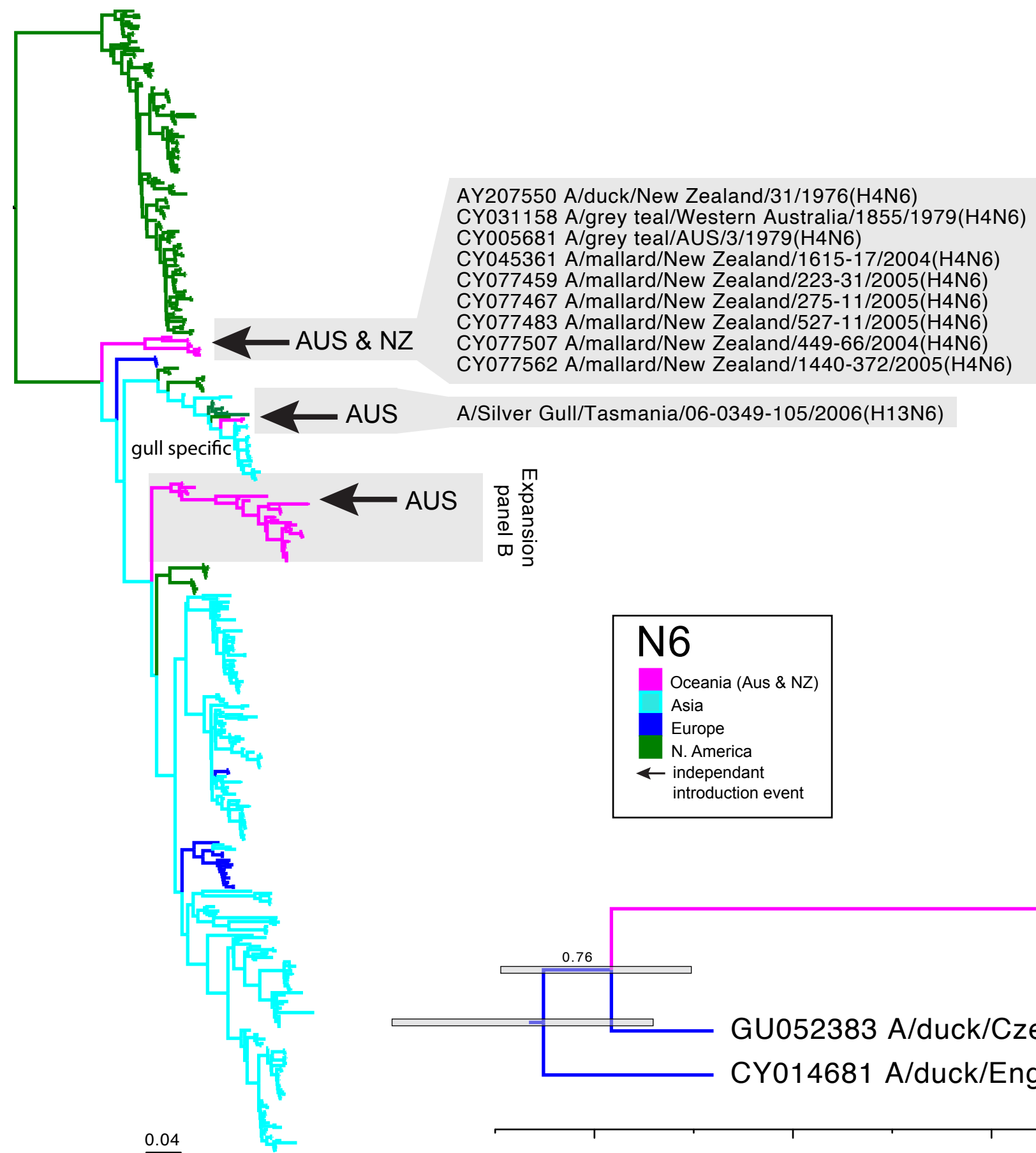

# B

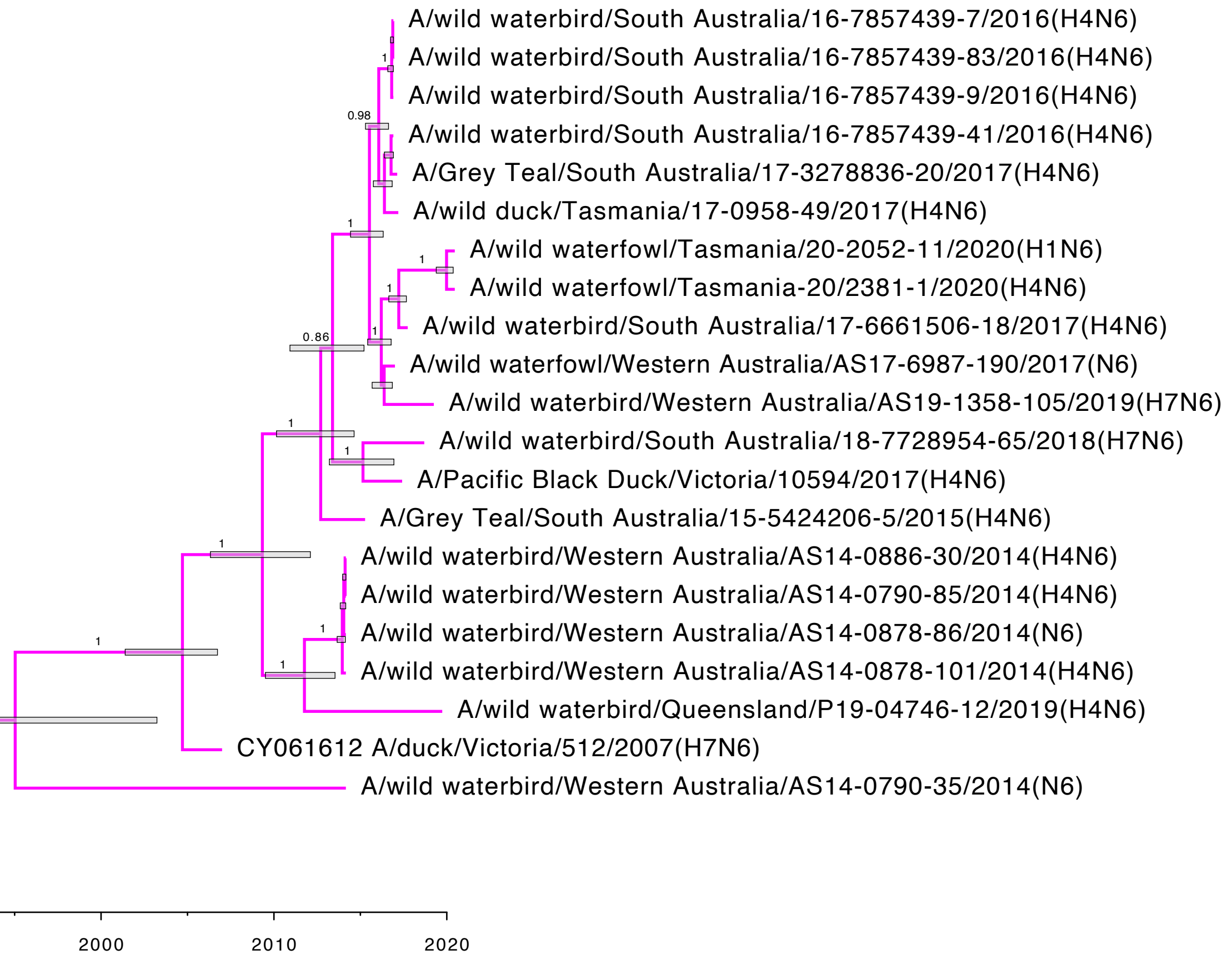

Supplement: S21 Fig — (A) Maximum likelihood tree of the sequences generated in this study, all sequences from Oceania in GenBank and reference sequences from Europe, Asia and North America. Lineages from Oceania are highlighted in grey boxes and virus names are provided. (B) Time structured phylogenetic tree comprising contemporary clades present in Australia. Node bars correspond to the 95% highest posterior density (HDP) of node height. Branches are coloured based on geography as indicated on the legend (PDF) [file ppat.1010150.s021.pdf]

A

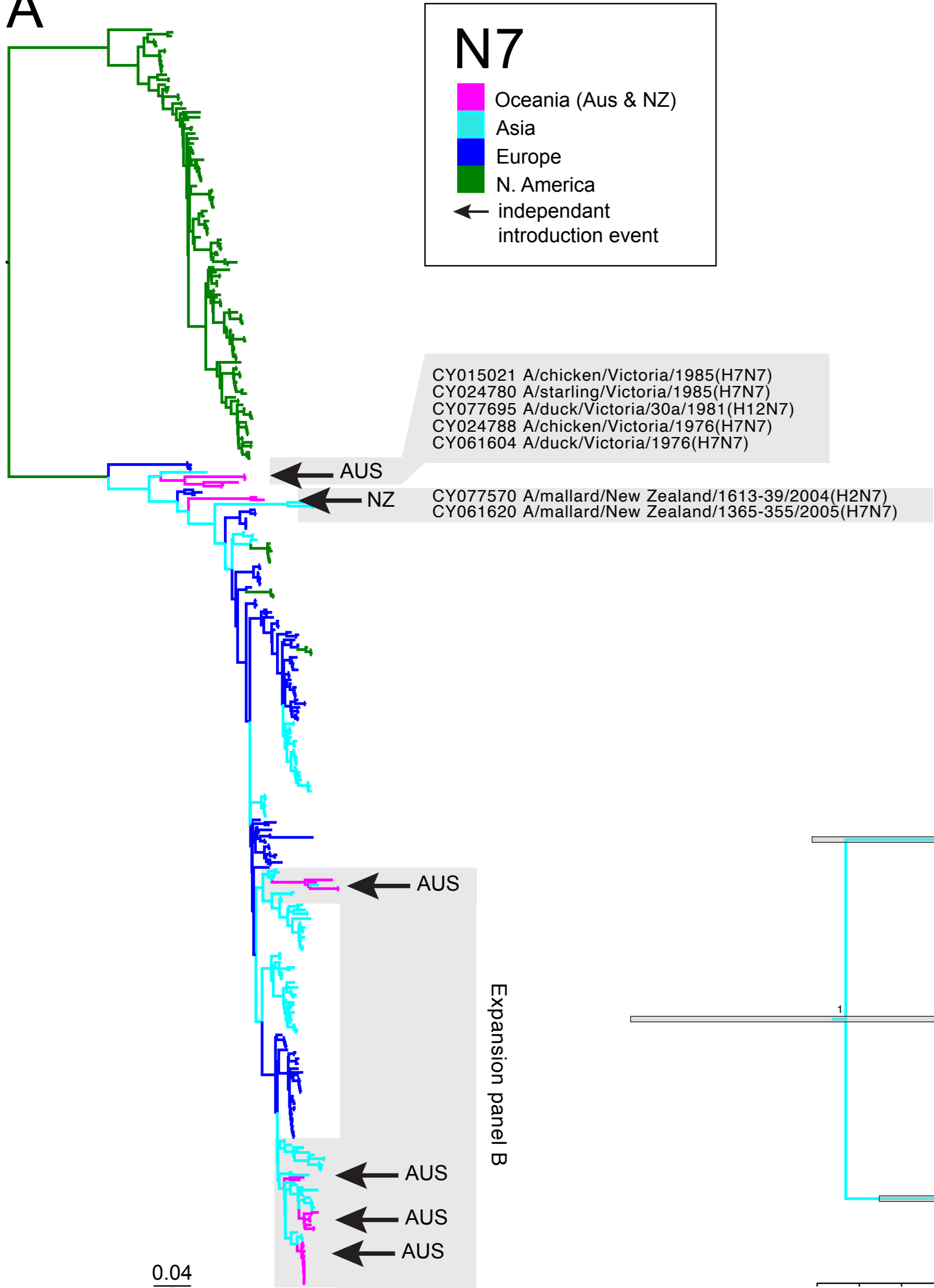

B

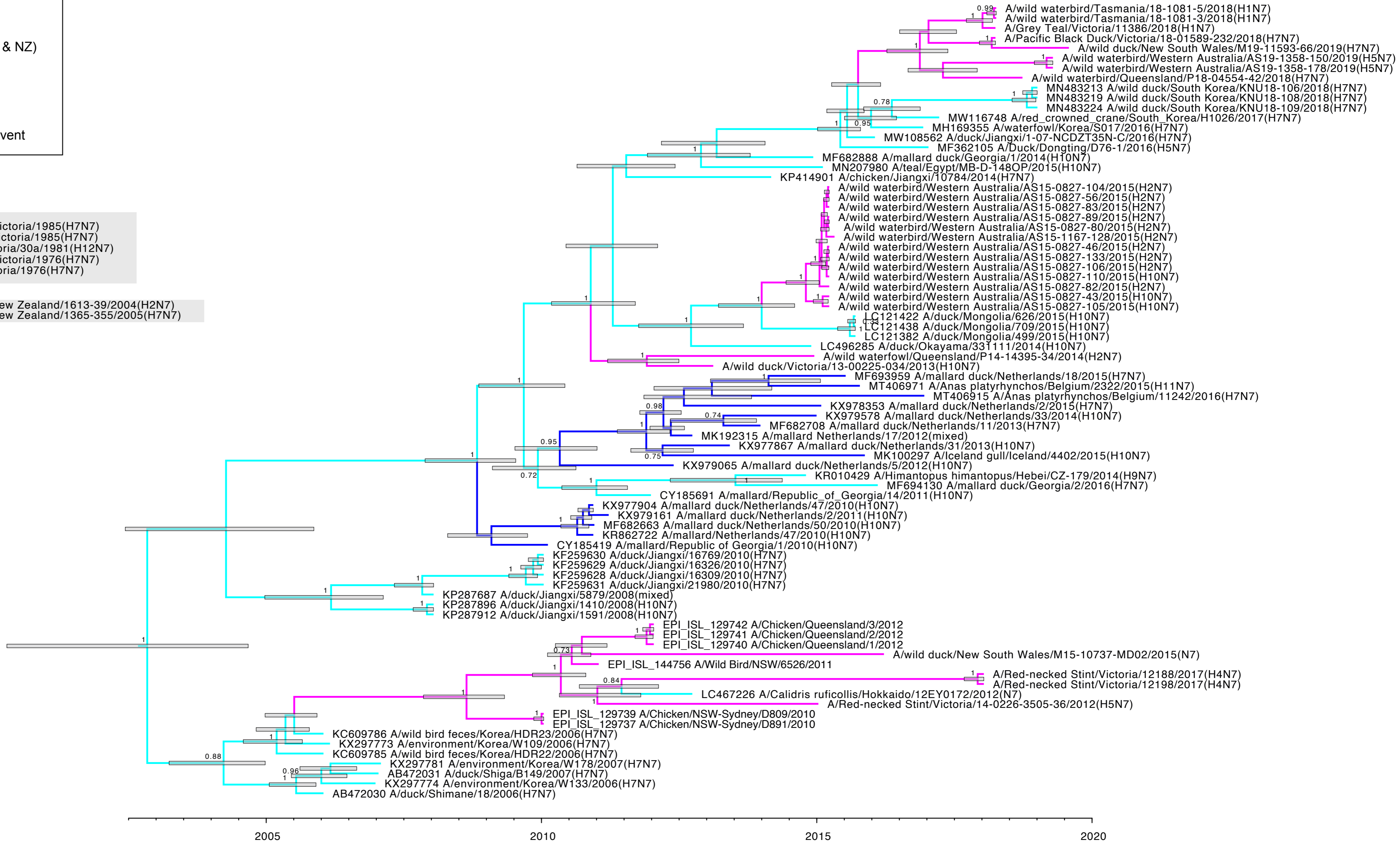

Supplement: S22 Fig — (A) Maximum likelihood tree of the sequences generated in this study, all sequences from Oceania in GenBank and reference sequences from Europe, Asia and North America. Lineages from Oceania are highlighted in grey boxes and virus names are provided. (B) Time structured phylogenetic tree comprising contemporary clades present in Australia. Node bars correspond to the 95% highest posterior density (HDP) of node height. Branches are coloured based on geography as indicated on the legend (PDF) [file ppat.1010150.s022.pdf]

A

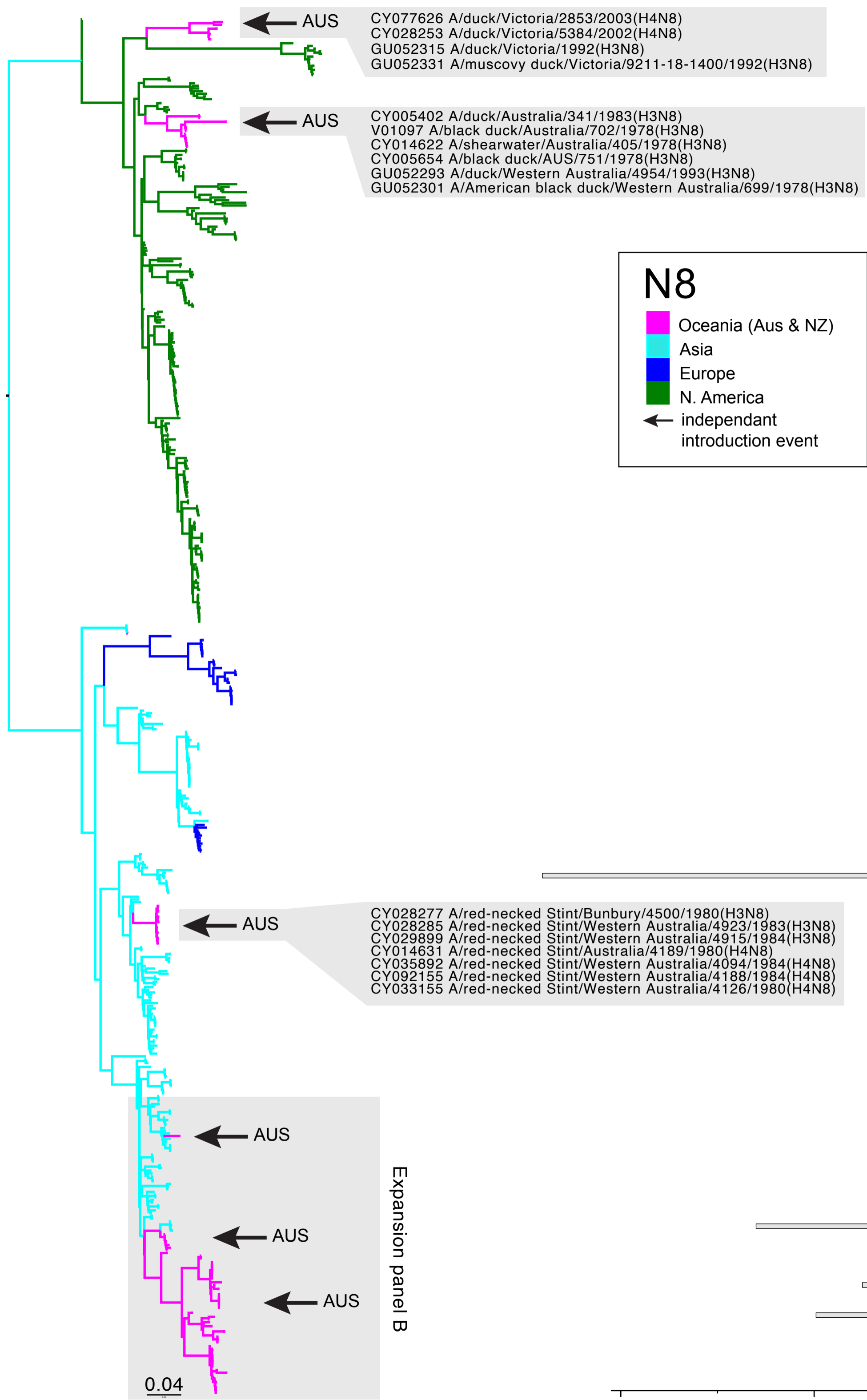

B

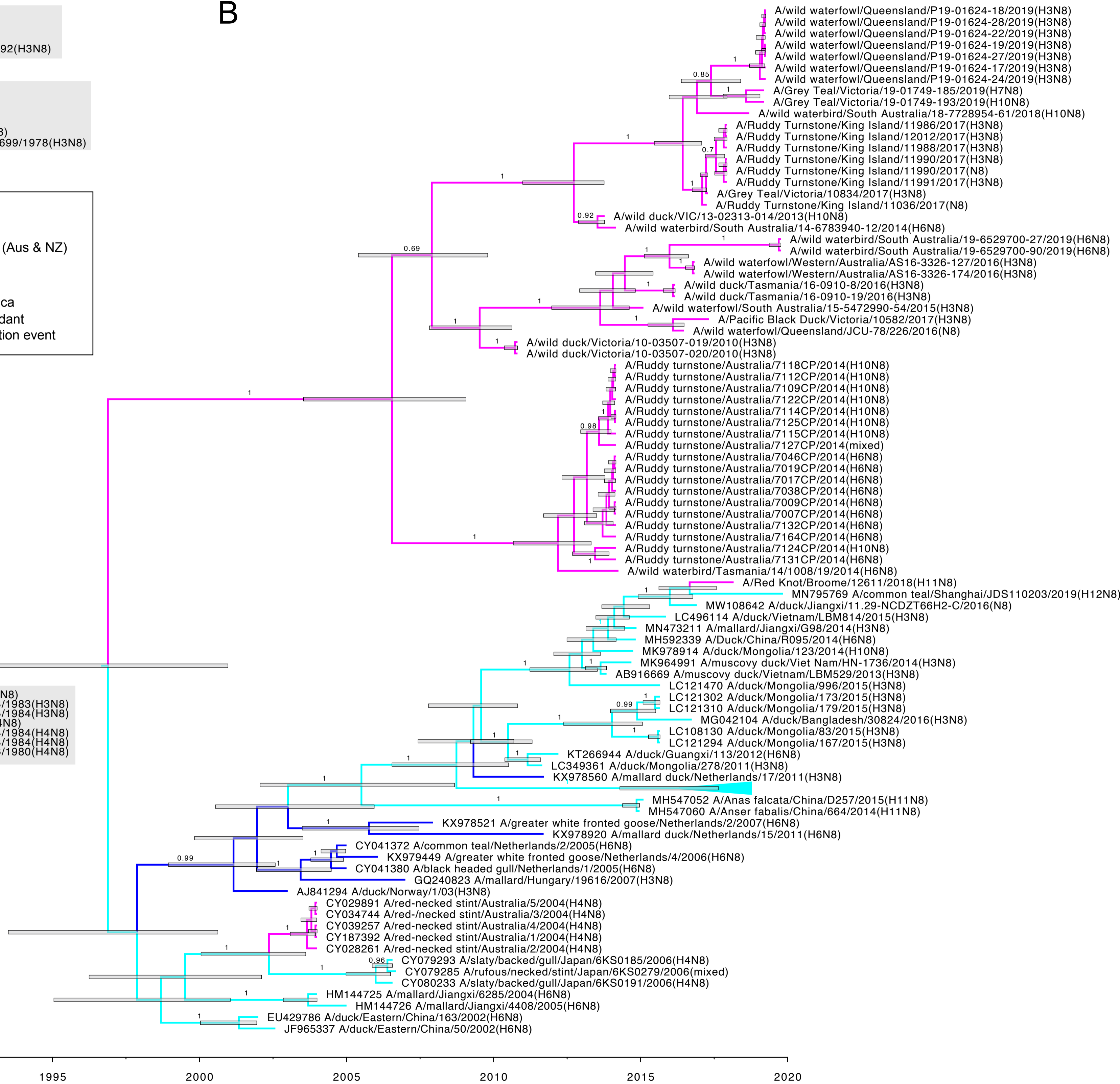

Supplement: S23 Fig — (A) Maximum likelihood tree of the sequences generated in this study, all sequences from Oceania in GenBank and reference sequences from Europe, Asia and North America. Lineages from Oceania are highlighted in grey boxes and virus names are provided. (B) Time structured phylogenetic tree comprising contemporary clades present in Australia. Node bars correspond to the 95% highest posterior density (HDP) of node height. Branches are coloured based on geography as indicated on the legend (PDF) [file ppat.1010150.s023.pdf]

A

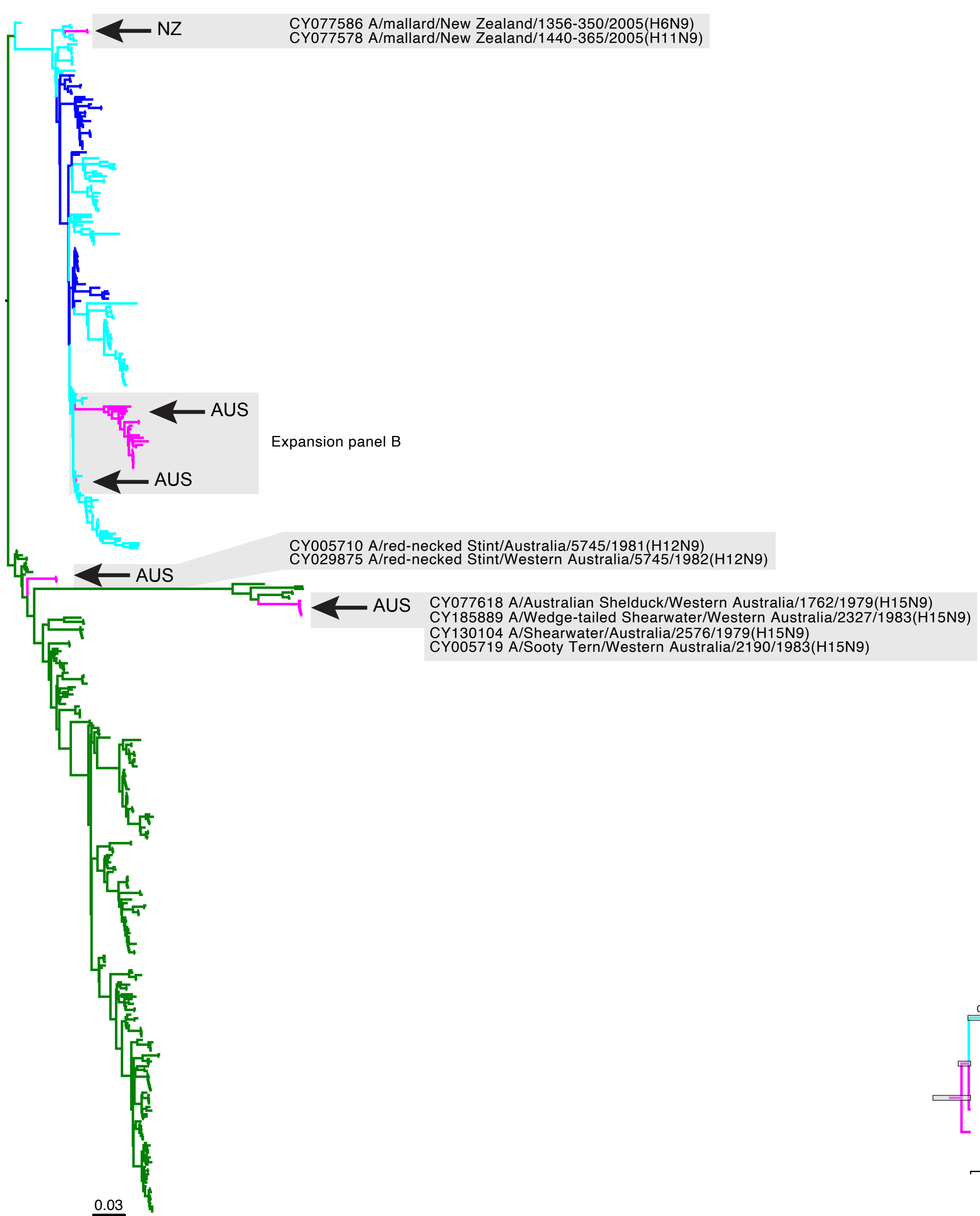

B

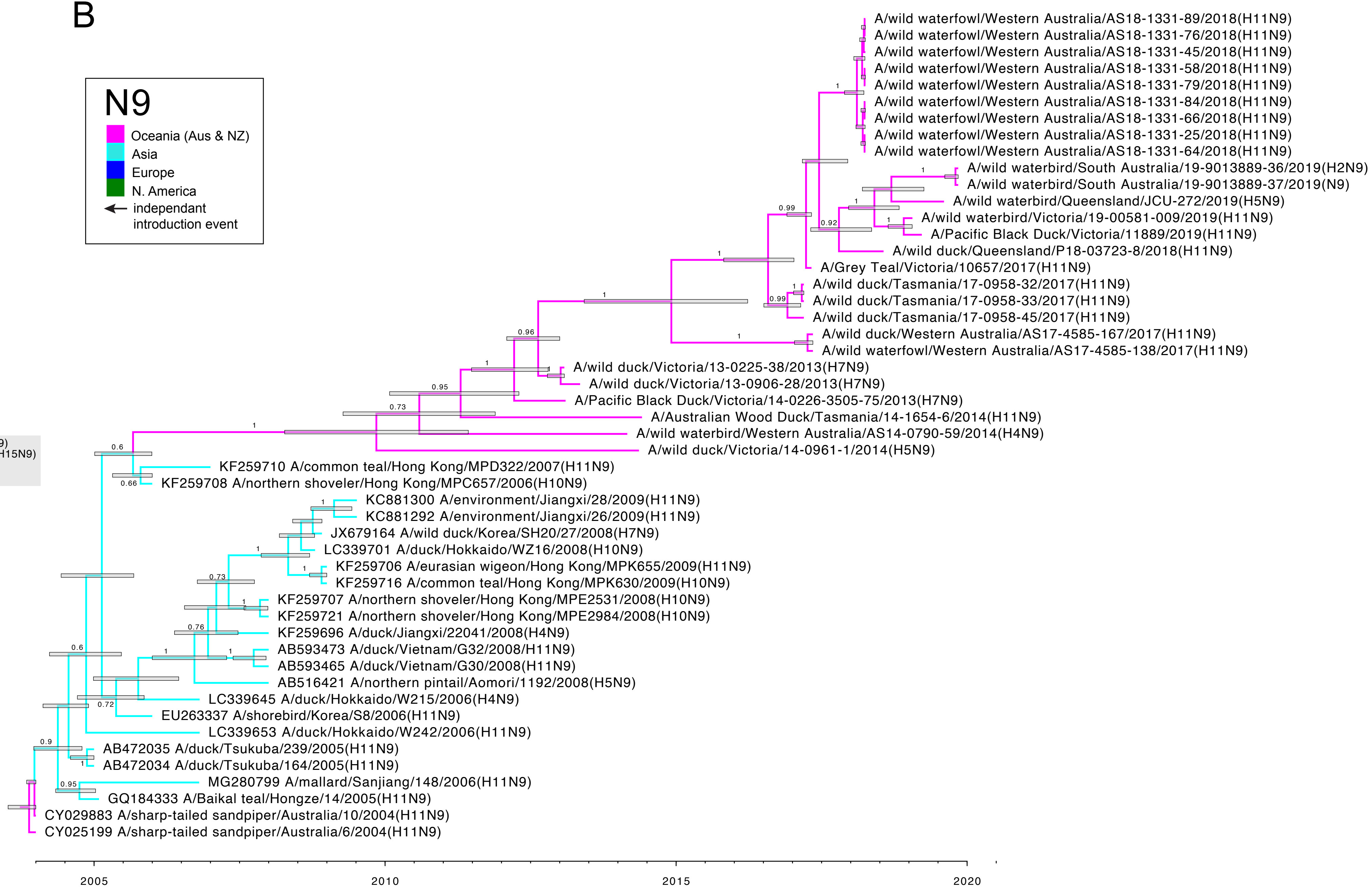

Supplement: S24 Fig — (A) Maximum likelihood tree of the sequences generated in this study, all sequences from Oceania in GenBank and reference sequences from Europe, Asia and North America. Lineages from Oceania are highlighted in grey boxes and virus names are provided. (B) Time structured phylogenetic tree comprising contemporary clades present in Australia. Node bars correspond to the 95% highest posterior density (HDP) of node height. Branches are coloured based on geography as indicated on the legend (PDF) [file ppat.1010150.s024.pdf]

PB2

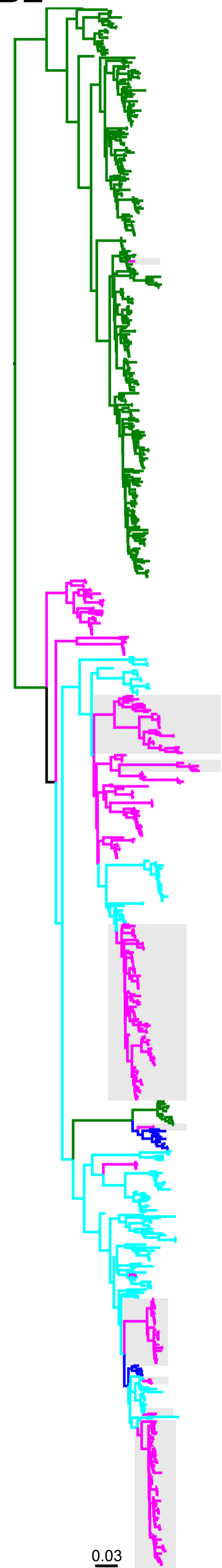

PB1

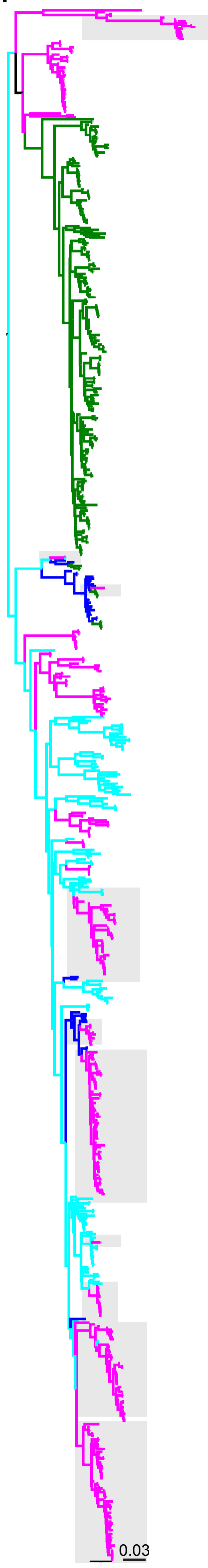

PA

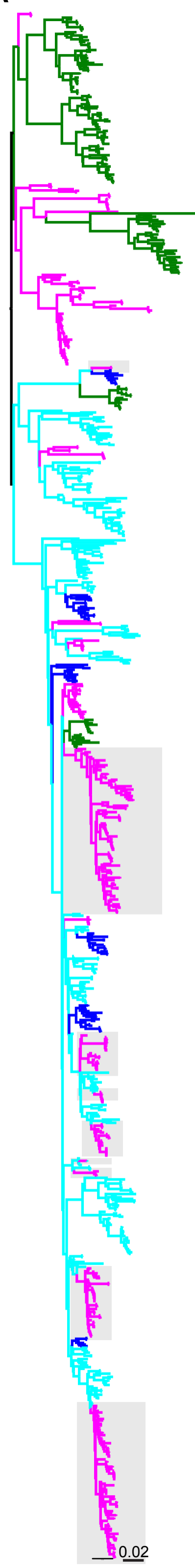

NP

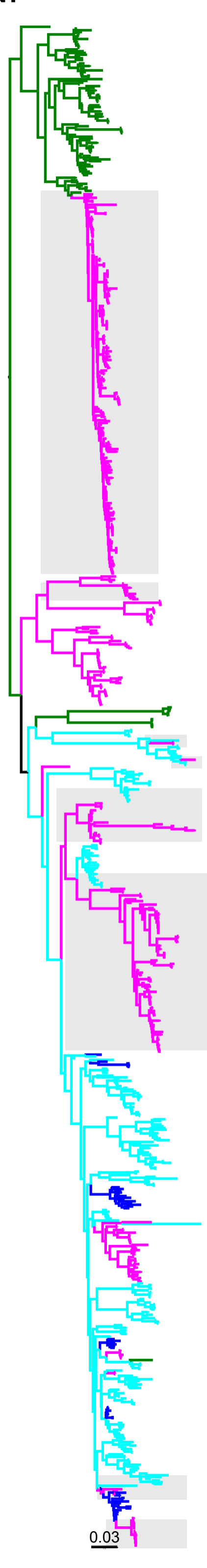

M

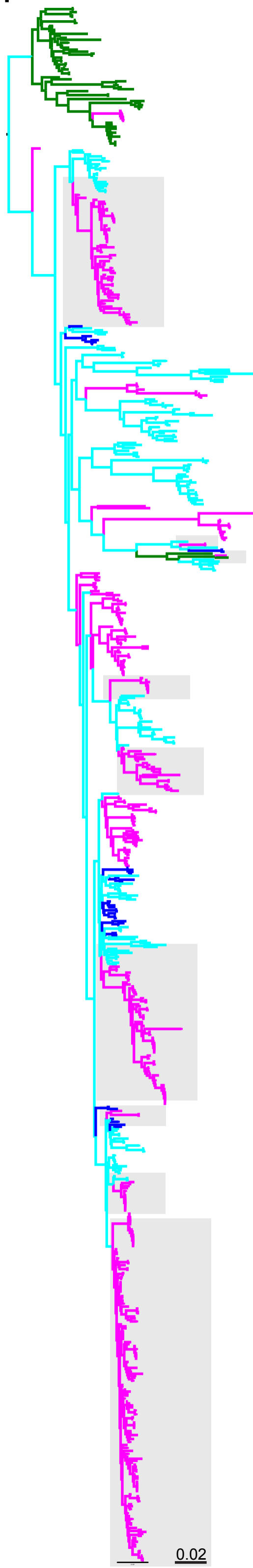

NS, A allele

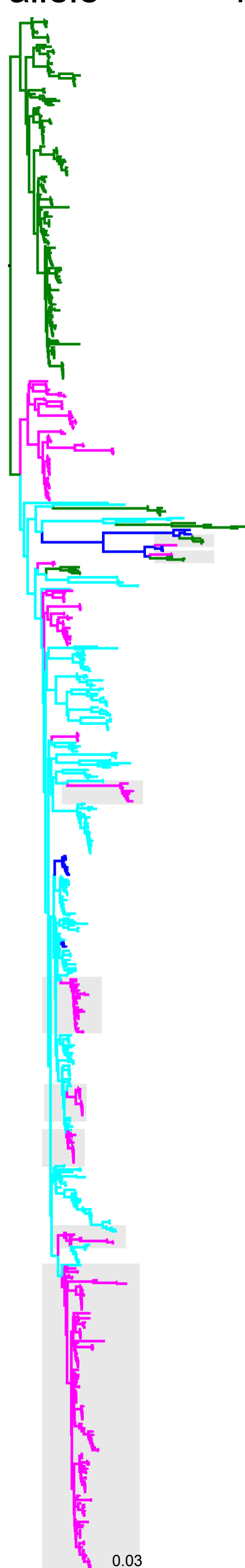

NS, B allele

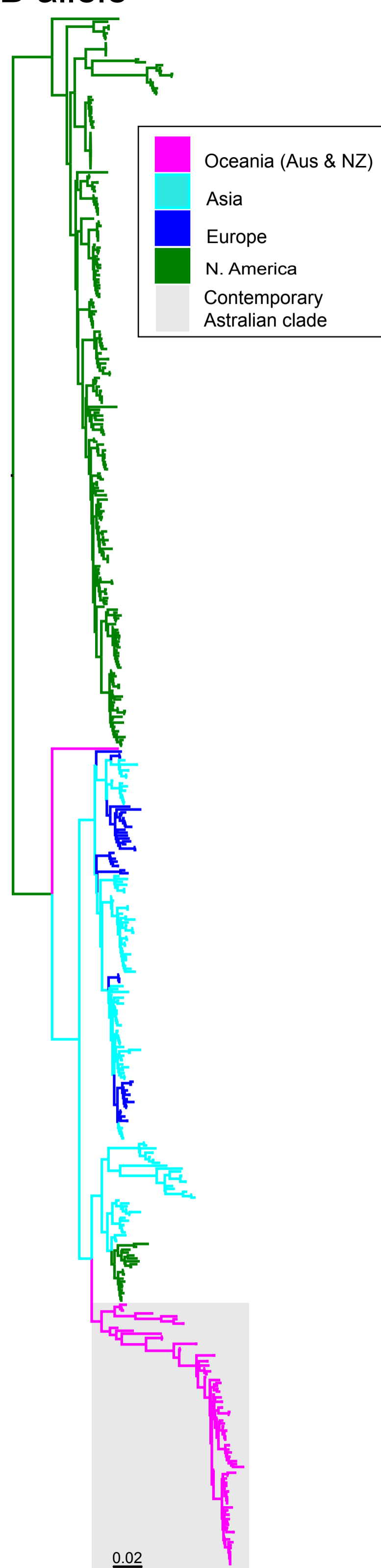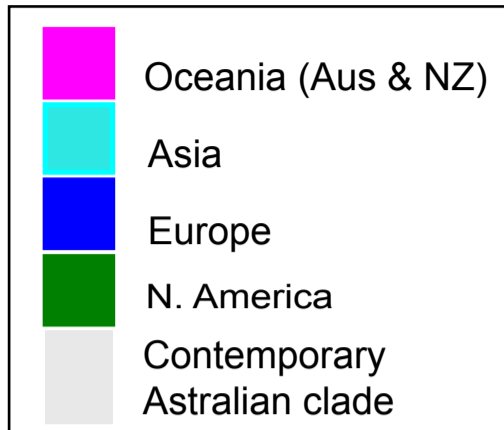

Supplement: S25 Fig — Trees are arranged by segment length–PB2, PB1, PA, NP, M and NS. Due to the large evolutionary distance between the two alleles of NS, these trees have been presented separately. Branches are coloured by continent. Shaded boxes indicate lineages currently circulating in Australia–pink lineages without grey boxes are extinct in Oceania. Trees have been rooted according to the evolutionary division between North America and Eurasia origin. Scale bar indicates the number of substitutions per site. A zoomed in version of NP is available in S25 Fig, in addition to time scale phylogenies of all currently circulating clades (PDF) [file ppat.1010150.s025.pdf]

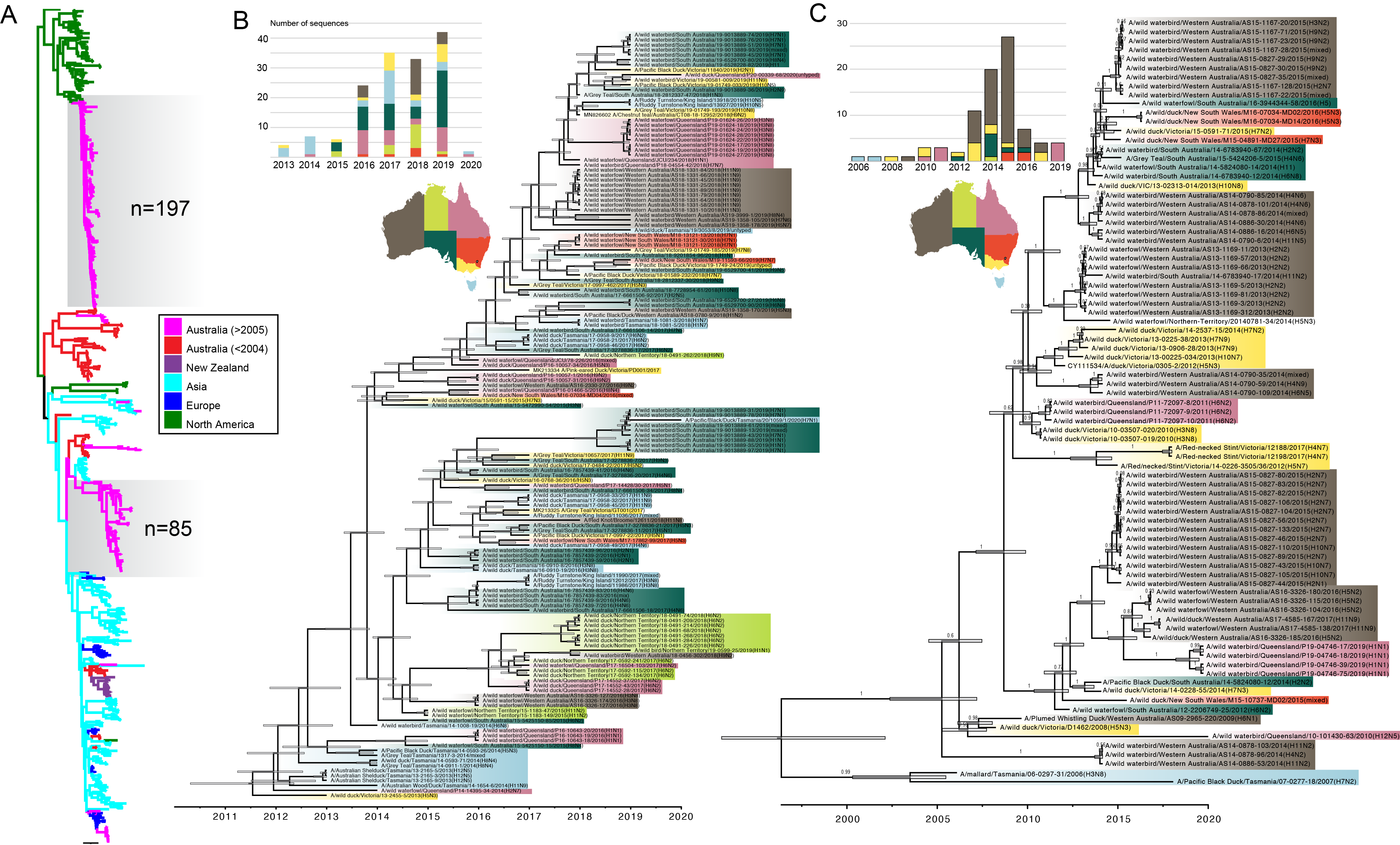

Supplement: S26 Fig — (A) Global maximum likelihood tree of the sequences generated in this study, all sequences from Oceania in GenBank and reference sequences from Europe, Asia and North America. (B, C) are time structured phylogenetic trees of the two largest clades present in Australia. Node bars correspond to the 95% highest posterior density (HDP) of node height. Number of sequences from each state are presented as a bar plot. Tips are coloured by Australian state. Map shapefile from mapsvg, distributed under a CC BY 4.0 licence. https://mapsvg.com/maps/australia (PNG) [file ppat.1010150.s026.png]

H5  
n=29

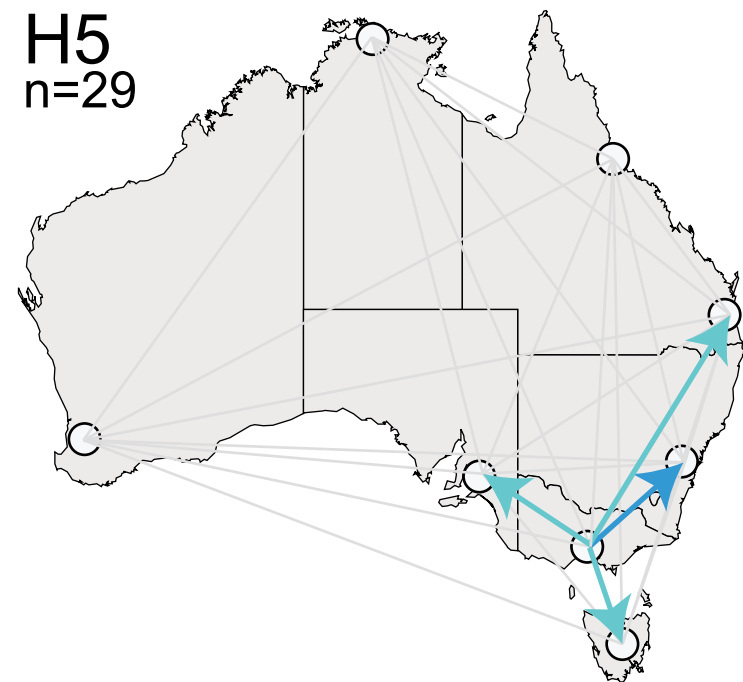

H7  
n=37

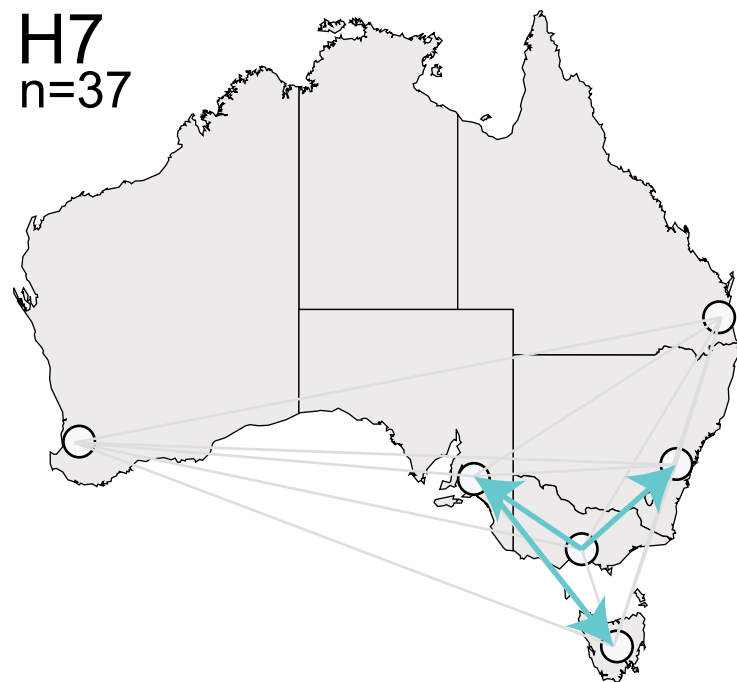

H4  
n=34

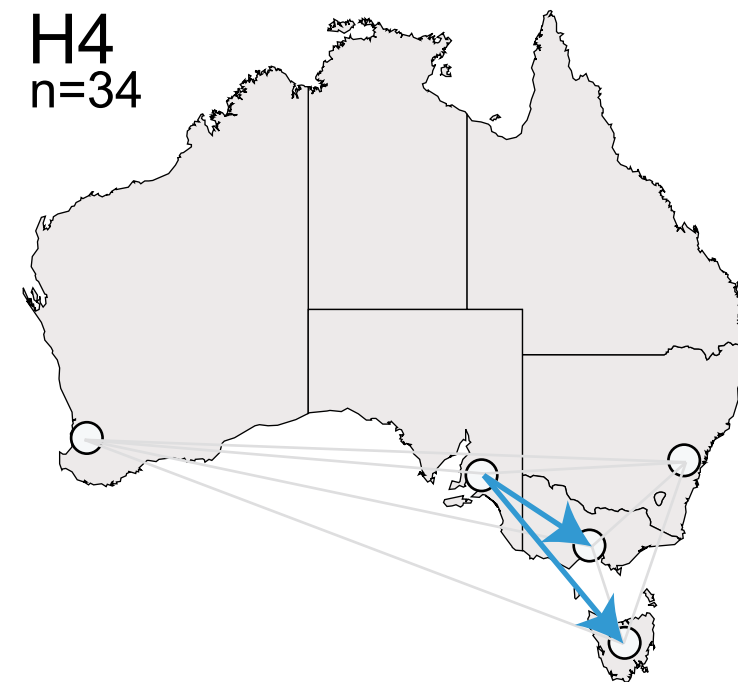

H6  
n=41

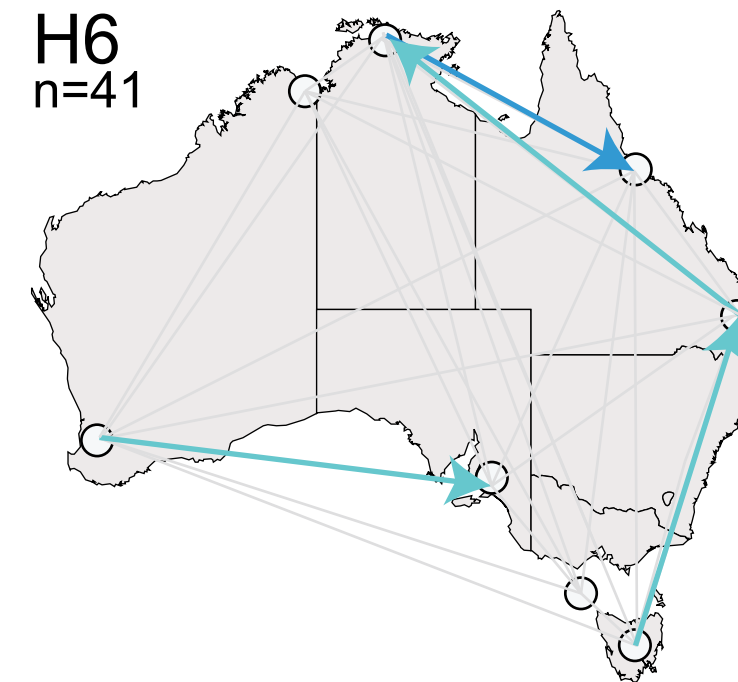

N6  
n=21

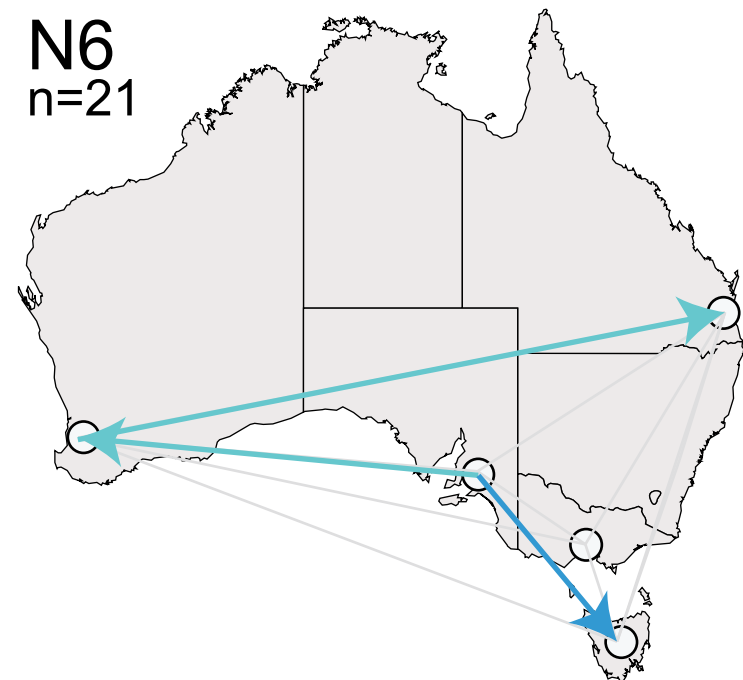

N8  
n=50

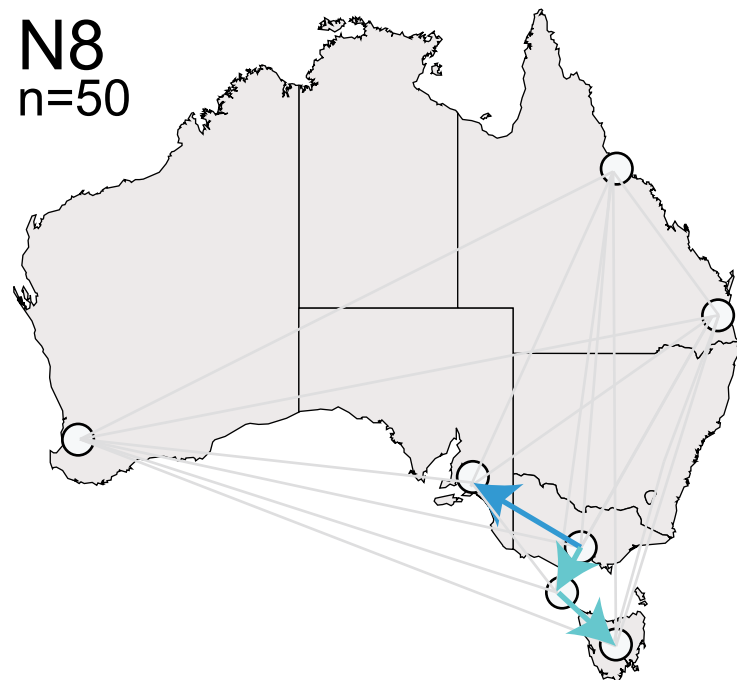

NP (1)  
n=197

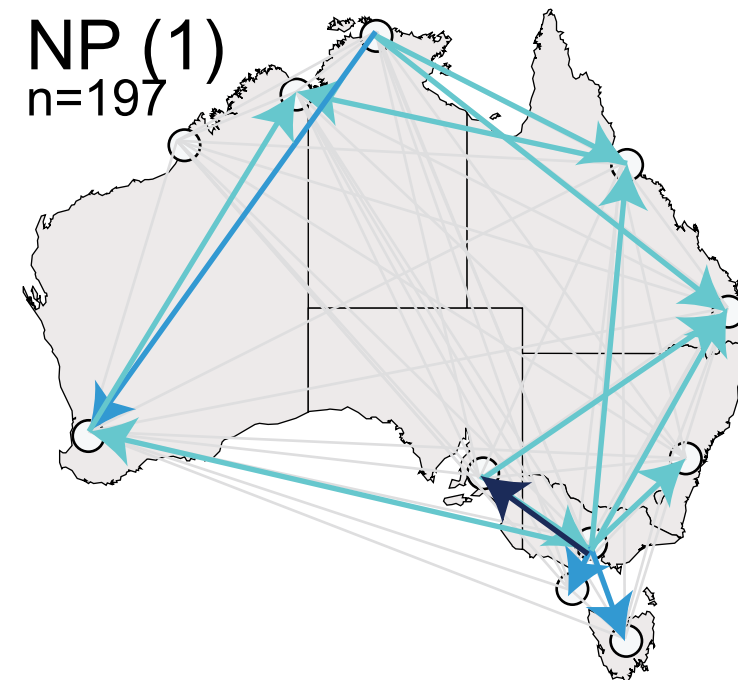

NP (2)  
n=85

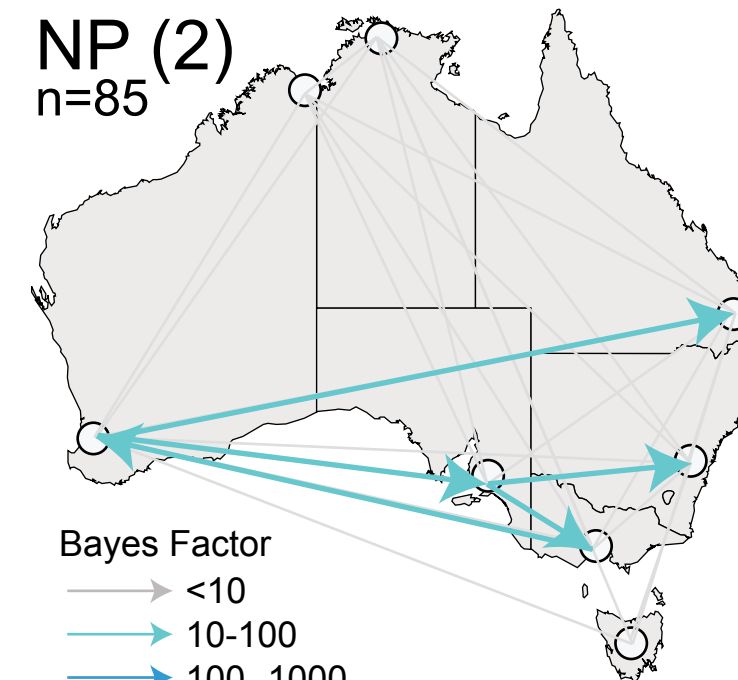

Bayes Factor

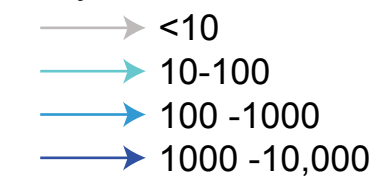

Supplement: S27 Fig — As NP has more than a single discrete Australian clade, we have generated two independent maps reflecting the 2 largest Australian clades of NP. Phylogeny of relevant segments can be found in Fig 3, S7–S9, S21, S23 and S26 Figs. Map shapefile from mapsvg, distributed under a CC BY 4.0 licence. https://mapsvg.com/maps/australia (PDF) [file ppat.1010150.s027.pdf]

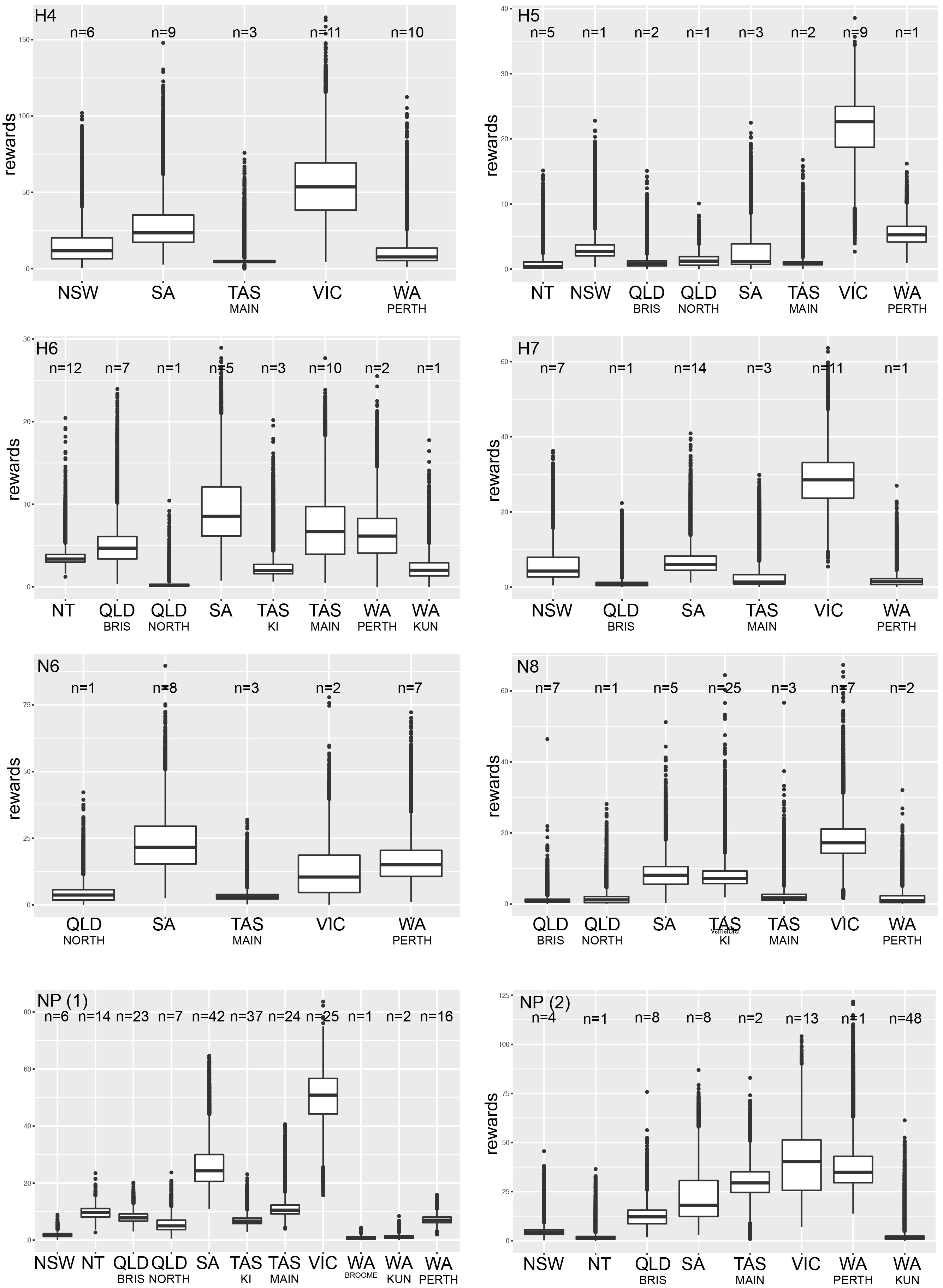

Supplement: S28 Fig — In cases where rewards are high, the state is central to exportation events. If patterns of Markov rewards are entirely explained by sample size (i.e, only those state with high samples sizes have high rewards) we may assume the model is explained by bias in sampling. (PNG) [file ppat.1010150.s028.png]

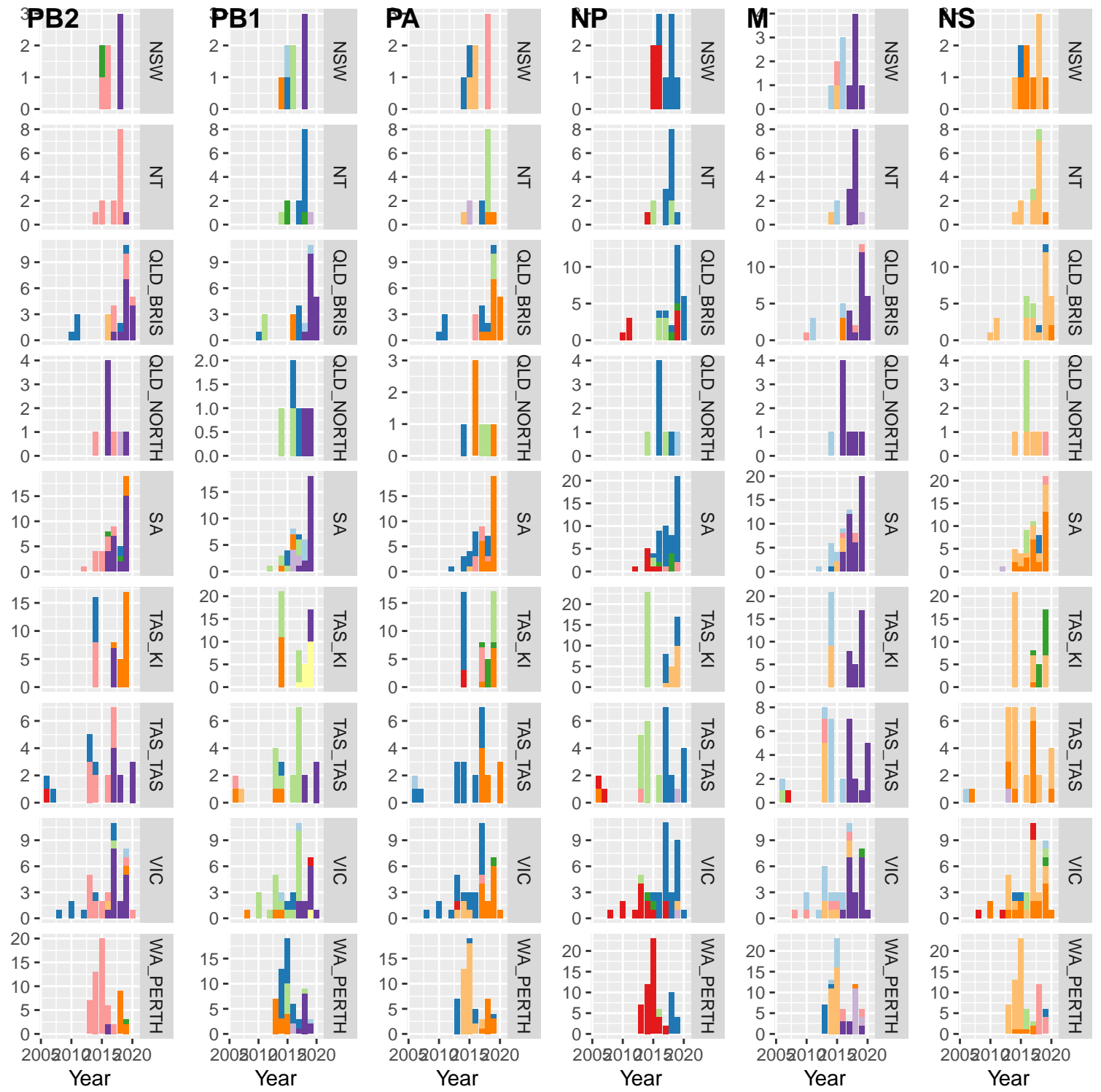

Supplement: S29 Fig — States are presented in rows and segment in columns. Colours refer to different lineages present, although they are presented in no particular order or colour. For most years, there is more than one lineage circulated in each state for all segments. (PDF) [file ppat.1010150.s029.pdf]
