# Supplementary material for: Australia as a global sink for the genetic diversity of avian influenza A virus
Source: PLoS Pathog. 2022 May 10;18(5):e1010150. doi: 10.1371/journal.ppat.1010150 (PMC9089890; doi:10.1371/journal.ppat.1010150)
Supplement: S9 Fig — (A) Maximum likelihood tree of the sequences generated in this study, all sequences from Oceania in GenBank and reference sequences from Europe, Asia and North America. Lineages from Oceania are highlighted in grey boxes and virus names are provided. (B) Time structured phylogenetic tree comprising contemporary clades present in Australia. Node bars correspond to the 95% highest posterior density (HDP) of node height. Branches are coloured based on geography as indicated on the legend (PDF) [file ppat.1010150.s009.pdf]

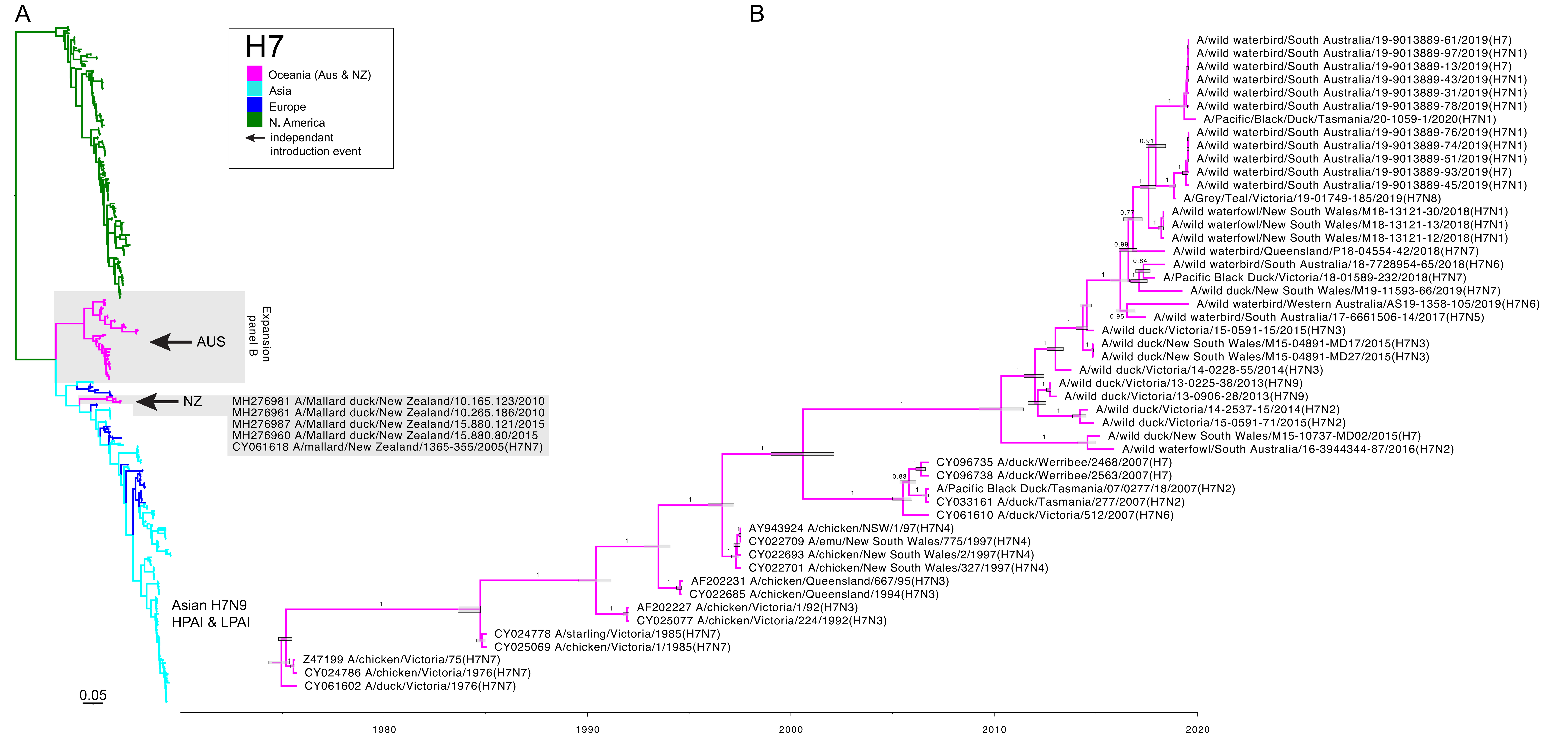
A
H7
Oceania (Aus & NZ)
Asia
Europe
N. America
independant introduction event
Expansion panel B
AUS
NZ
MH276981 A/Mallard duck/New Zealand/10.165.123/2010
MH276961 A/Mallard duck/New Zealand/10.265.186/2010
MH276987 A/Mallard duck/New Zealand/15.880.121/2015
MH276960 A/Mallard duck/New Zealand/15.880.80/2015
CY061618 A/mallard/New Zealand/1365-355/2005(H7N7)
Asian H7N9
HPAI & LPAI
0.05
B
A/wild waterbird/South Australia/19-9013889-61/2019(H7)
A/wild waterbird/South Australia/19-9013889-97/2019(H7N1)
A/wild waterbird/South Australia/19-9013889-13/2019(H7)
A/wild waterbird/South Australia/19-9013889-43/2019(H7N1)
A/wild waterbird/South Australia/19-9013889-31/2019(H7N1)
A/wild waterbird/South Australia/19-9013889-78/2019(H7N1)
A/Pacific/Black/Duck/Tasmania/20-1059-1/2020(H7N1)
A/wild waterbird/South Australia/19-9013889-76/2019(H7N1)
A/wild waterbird/South Australia/19-9013889-74/2019(H7N1)
A/wild waterbird/South Australia/19-9013889-51/2019(H7N1)
A/wild waterbird/South Australia/19-9013889-93/2019(H7)
A/wild waterbird/South Australia/19-9013889-45/2019(H7N1)
A/Grey/Teal/Victoria/19-01749-185/2019(H7N8)
A/wild waterfowl/New South Wales/M18-13121-30/2018(H7N1)
A/wild waterfowl/New South Wales/M18-13121-13/2018(H7N1)
A/wild waterfowl/New South Wales/M18-13121-12/2018(H7N1)
A/wild waterbird/Queensland/P18-04554-42/2018(H7N7)
A/wild waterbird/South Australia/18-7728954-65/2018(H7N6)
A/Pacific Black Duck/Victoria/18-01589-232/2018(H7N7)
A/wild duck/New South Wales/M19-11593-66/2019(H7N7)
A/wild waterbird/Western Australia/AS19-1358-105/2019(H7N6)
A/wild waterbird/South Australia/17-6661506-14/2017(H7N5)
A/wild duck/Victoria/15-0591-15/2015(H7N3)
A/wild duck/New South Wales/M15-04891-MD17/2015(H7N3)
A/wild duck/New South Wales/M15-04891-MD27/2015(H7N3)
A/wild duck/Victoria/14-0228-55/2014(H7N3)
A/wild duck/Victoria/13-0225-38/2013(H7N9)
A/wild duck/Victoria/13-0906-28/2013(H7N9)
A/wild duck/Victoria/14-2537-15/2014(H7N2)
A/wild duck/Victoria/15-0591-71/2015(H7N2)
A/wild duck/New South Wales/M15-10737-MD02/2015(H7)
A/wild waterfowl/South Australia/16-3944344-87/2016(H7N2)
CY096735 A/duck/Werribee/2468/2007(H7)
CY096738 A/duck/Werribee/2563/2007(H7)
A/Pacific Black Duck/Tasmania/07/0277/18/2007(H7N2)
CY033161 A/duck/Tasmania/277/2007(H7N2)
CY061610 A/duck/Victoria/512/2007(H7N6)
AY943924 A/chicken/NSW/1/97(H7N4)
CY022709 A/emu/New South Wales/775/1997(H7N4)
CY022693 A/chicken/New South Wales/2/1997(H7N4)
CY022701 A/chicken/New South Wales/327/1997(H7N4)
AF202231 A/chicken/Queensland/667/95(H7N3)
CY022685 A/chicken/Queensland/1994(H7N3)
AF202227 A/chicken/Victoria/1/92(H7N3)
CY025077 A/chicken/Victoria/224/1992(H7N3)
CY024778 A/starling/Victoria/1985(H7N7)
CY025069 A/chicken/Victoria/1/1985(H7N7)
Z47199 A/chicken/Victoria/75(H7N7)
CY024786 A/chicken/Victoria/1976(H7N7)
CY061602 A/duck/Victoria/1976(H7N7)
1980
1990
2000
2010
2020
