# Supplementary material for: Australia as a global sink for the genetic diversity of avian influenza A virus
Source: PLoS Pathog. 2022 May 10;18(5):e1010150. doi: 10.1371/journal.ppat.1010150 (PMC9089890; doi:10.1371/journal.ppat.1010150)
Supplement: S2 Table — (DOCX) [file ppat.1010150.s031.docx]

***S2 Table.*** *Details of mixed viruses detected in this study*

| **Organism name** | **State** | **HA** | **NA** | **Passage History** | **Sample type** | **Other viruses sequenced from same capture event** |
| --- | --- | --- | --- | --- | --- | --- |
| A/wild duck/New South Wales/M15-10737-MD02/2015(mixed) | NSW | H7 | N7,N1 | original | faecal swab | none |
| A/wild waterbird/Western Australia/AS15-0827-35/2015(mixed) | WA | H9 | N1,N2 | original | faecal swab | Event 0827: H2N7, H10N7 |
| A/Ruddy Turnstone/King Island/11036/2017(mixed) | Tas | H9,H3 | N3,N8 | original | Combined cloacal and oropharyngeal swab | H12N5, H3N8 |
| A/Ruddy Turnstone/King Island/11990/2017(mixed) | Tas | H9,H3 | N3,N8 | original | Combined cloacal and oropharyngeal swab | H12N5, H3N8 |
| A/wild waterbird/South Australia/19-9013889-61/2019(mixed) | SA | H7,H1 | N1 | original | pooled faecal swabs | Event: 9013889: H2N3, H7N1, H1N1, H2N9 |
| A/wild waterbird/South Australia/19-9013889-93/2019(mixed) | SA | H7,H2 | N1 | original | pooled faecal swabs | Event: 9013889: H2N3, H7N1, H1N1, H2N9 |
| A/wild waterbird/South Australia/19-9013889-13/2019(mixed) | SA | H7 | N3,N1 | original | pooled faecal swabs | Event: 9013889: H2N3, H7N1, H1N1, H2N9 |
| A/wild waterbird/Western Australia/AS14-0790-35/2014(mixed) | WA | H4 | N6,N2 | E1 | unspecified swab | Event: 0790: H11N5, H4N9, H4N6, H6N5 |
| A/wild waterbird/Western Australia/AS14-0878-86/2014(mixed) | WA | H4 | N6,N2 | lyophilised AF | pooled unspecified swabs | Event: 0878: H11N2, H4N6 |
| A/wild waterbird/Western Australia/AS15-1167-22/2015(mixed) | WA | H3 | N1,N2 | E1 | pooled faecal swabs | Event 1167: H3N2, H9N2, H2N7 |
| A/wild waterbird/Western Australia/AS15-1167-28/2015(mixed) | WA | H3 | N1,N2 | E2 | pooled faecal swabs | Event 1167: H3N2, H9N2, H2N7 |
| A/wild waterfowl/Queensland/JCU-78-226/2016(mixed) | QLD | H3 | N3,N8 | E1 | faecal swab | none |
| A/Ruddy turnstone/Australia/7127CP/2014(H6/H10N8) | TAS | H6, H10 | N8 | E1 | Combined cloacal and oropharyngeal swab | H3N5, H6N8, H10N8 |
